# Sequence similarity governs generalizability of *de novo* deep learning models for RNA secondary structure prediction

**DOI:** 10.1371/journal.pcbi.1011047

**Published:** 2023-04-17

**Authors:** Xiangyun Qiu

**Affiliations:** Department of Physics, George Washington University, Washington DC, United States of America; University of Rochester, UNITED STATES

## Abstract

Making no use of physical laws or co-evolutionary information, *de novo* deep learning (DL) models for RNA secondary structure prediction have achieved far superior performances than traditional algorithms. However, their statistical underpinning raises the crucial question of generalizability. We present a quantitative study of the performance and generalizability of a series of *de novo* DL models, with a minimal two-module architecture and no post-processing, under varied similarities between seen and unseen sequences. Our models demonstrate excellent expressive capacities and outperform existing methods on common benchmark datasets. However, model generalizability, i.e., the performance gap between the seen and unseen sets, degrades rapidly as the sequence similarity decreases. The same trends are observed from several recent DL and machine learning models. And an inverse correlation between performance and generalizability is revealed collectively across all learning-based models with wide-ranging architectures and sizes. We further quantitate how generalizability depends on sequence and structure identity scores via pairwise alignment, providing unique quantitative insights into the limitations of statistical learning. Generalizability thus poses a major hurdle for deploying *de novo* DL models in practice and various pathways for future advances are discussed.

## Author summary

Learning-based *de novo* models of RNA secondary structures critically rely on training data to associate sequences with structures. The practical utility of such models thus hinges on not only training performances but generalizations over unseen sequences. Model generalizability, however, remains poorly understood. By delineating sequence similarity at three distinct levels, we develop a series of DL models and evaluate their performance and generalizability, as well as several current DL and machine learning models. First establishing the decisive role of sequence similarity in generalizability, we further quantitate their dependencies via pairwise sequence and structure alignment. The gained quantitative insights make valuable guidelines for deploying DL models in practice and advocate RNA secondary structure as a unique platform for developing generalizable learning-based models.

## Introduction

As a linear chain of nucleotides capable of base pairing, an RNA molecule readily forms various secondary structure motifs such as stems and loops, regardless of the foldability of tertiary structures [1–3]. Particularly for the diverse families of non-coding RNAs [4], their secondary structures are more conserved than sequences and provide important cues for their biological functions [5,6]. Even messenger RNAs possess key secondary structure motifs for translation regulation [7–9]. As such, there have been major interests in determining and understanding RNA secondary structures, via both experiment and computation [10–12]. In recent years, with the emergence of sizeable RNA structure databases and the accessibility of powerful artificial neural networks, data-centric deep-learning-based models, the subject of this study, have been successfully developed for RNA secondary structure prediction [13].

RNA secondary structures can be defined at the individual base or base-pair level. The base-level descriptions include the pairing probability and the structure motif of each base, as well as solvent accessibility and other structural or biochemical features. Here we focus on the pair-level description identifying all base pairs in terms of binary classification. As any two nucleotides (i.e., AUCG) can pair up in theory [14], the native set of base pairs represents the single optimal state among all possible configurations. It should be noted that multiple, or even a myriad of, sets of base pairs can have comparable likelihoods and are better treated as an ensemble, which however is beyond the scope of this study. Such an optimal set is best inferred from the covariance patterns of homologous sequences which however can be costly or impossible to obtain [10,15]. In lieu of co-evolutionary information, traditional *de novo* approaches generally represent the secondary structure as a graph with nucleotides as nodes and base pairs as edges (or similarly a parse tree with lone and paired leaves). A score is then computed according to pre-defined structural elements of the graph (or tree), using parameters derived from measurements of thermodynamic energies [16], data mining of known structures, or a combination of both [17]. The onus is on the algorithms to optimize the score by searching the entire structure space, which grows exponentially with the sequence length. To reduce the computational complexity, various rules of RNA secondary structures have been introduced, such as non-nested base pairing (i.e., no pseudoknots), canonical base pairs only (i.e., AU, GC, and GU), and no sharp turns. Efficient dynamic programming and related techniques [18] have also been introduced along with improved scoring parameters [16,17]. However, traditional algorithms have struggled to make significant gains in performance in the recent decades [13].

A major advance in prediction performance comes from recent applications of deep learning (DL). Instead of the graph or tree search, DL models represent the secondary structure as a 2D pairing probability matrix (PPM) and directly predict every PPM_*ij*_ (*i* and *j* are nucleotide indices) in parallel. In doing so, DL models often employ many abstraction layers enlisting millions of parameters that must be learned via training on known structures. As existing large RNA secondary structure datasets are curated largely via comparative sequence analysis [19,20], this study focuses on the class of single-sequence-based DL models, referred to as *de novo* DL models. A number of highly successful *de novo* DL models have been reported, such as 2dRNA [21], ATTfold [22], DMfold [23], E2Efold [24], MXfold2 [25], SPOT-RNA [26], and Ufold [27], among others [28–31]. These DL models markedly outperform traditional algorithms, with even close-to-perfect predictions in some cases, though questions on the training vs. test similarity have been raised [32,33] and discussed below. It is worth noting that DL models have also been developed for base-level prediction tasks such as the pairing probability of each base [34]. Notwithstanding, *de novo* DL models have emerged as a promising powerful solution to the RNA secondary structure problem.

Despite the successes, key questions remain as to the practical utility of *de novo* DL models. One is that DL models have yet to reach decent performances on all known datasets. For example, the best test F1 score for the largest dataset, bpRNA [19], remains low, ~0.65, by all DL and traditional models. Arguably more critical, another issue concerns the generalizability of *de novo* DL models, i.e., how models perform on an unseen/test set of sequences compared with the seen/training set. Substantial performance drops would indicate poor generalizability to which learning-based models are highly susceptible. To enhance the generalizability of models, various regularization techniques have been utilized during the training stage, including L1/L2 regularization and dropouts, as well as ensemble learning and the integration of DL-predicted folding scores with thermodynamic regulation, as seen in SPOT-RNA and MXfold2, respectively. These techniques require knowledge of training data and modify the values of trainable parameters, with the exception of plain model averaging. We refer to these techniques as “model regularization”, which distinguishes from the post-training operations on secondary structures by certain learning-based models, which are referred to as “post-processing” in this study. An example of post-processing is the iterative refinement of predicted PPMs to enforce structure constraints, as demonstrated in E2Efold and Ufold. Despite common uses of regularization techniques, the reported performances of DL models are strongly dataset-dependent; models can only be benchmarked with the same pair of training and test datasets. Crucially, poor generalizability is generally observed for test sequences with structures that are out of distribution with respect to the training data, often referred to family-fold, inter-family, or family-wise validation. Since comparative sequence analysis is the method of choice for intra-family sequences, such *de novo* DL models would be of little practical utility should the issue of generalizability remain unresolved.

The subject of generalizability of DL models has also been the focus of two recent studies. Szikszai *et al*. [32] first demonstrated that base-level pseudo-free energies predicted by a basic 1D convolutional neural network, when coupled with traditional dynamic programming (RNAstructure [35]), suffice to attain excellent performances on test data in the same RNA families as the training data. However, markedly worse performances were observed for test sequences in different families as the training set, and retraining several current DL models led to similar observations. In the other study [33], Flamm *et al*. explored the learning and generalization capacities of various neural networks with synthetic RNA sequences folded into secondary structures by a consistent thermodynamic model (RNAfold [36]), as well as inverse-folded sequences from true structures in the bpRNA dataset. Their approach circumvents the unbalanced distributions and potential errors of available datasets and further allows engineered biases in RNA sequence or structure. Effectively learning from a thermodynamic model, neural networks were shown to fail to generalize over sequences of different lengths and, more importantly, over sequences whose structures are not in the training data. In all, these non-data-agnostic behaviors stand as a major hurdle for deploying *de novo* DL models. However, model generalizability remains poorly understood at the practical and systematic level, especially its quantitative dependence on sequence distributions for end-to-end DL models trained on commonly used databases.

To this end, we investigate the performance and generalizability of a series of *de novo* DL models of different sizes under varied sequence distributions. Compared with the two recent studies [32,33], we focus on the development and analysis of end-to-end DL models that can match or outperform the state-of-the-art *de novo* DL models when trained on the same public datasets at well-defined similarity levels, and we take advantage of RNA alignment tools to elicit model characteristics quantitatively. Specifically, we chose a minimal two-module architecture without post-processing so as to probe intrinsic model characteristics. We found that a small DL model of 16K parameters can achieve decent performances on a medium-sized dataset and that medium-sized models with less than 1M parameters can attain excellent performances and surpass existing DL and traditional models. However, model generalization deteriorates as the sequence similarity between the seen and unseen datasets decreases. To gain quantitative insights, we determined how model generalizability depends on sequence and structure similarities via pairwise sequence and structure alignment. Our observations affirm that *de novo* DL models are largely statistical learners of RNA sequence vs. structure correlations and we last discuss various pathways to improve the generalizability of *de novo* DL models.

## Results

### Overview of our study design

**Fig 1:**
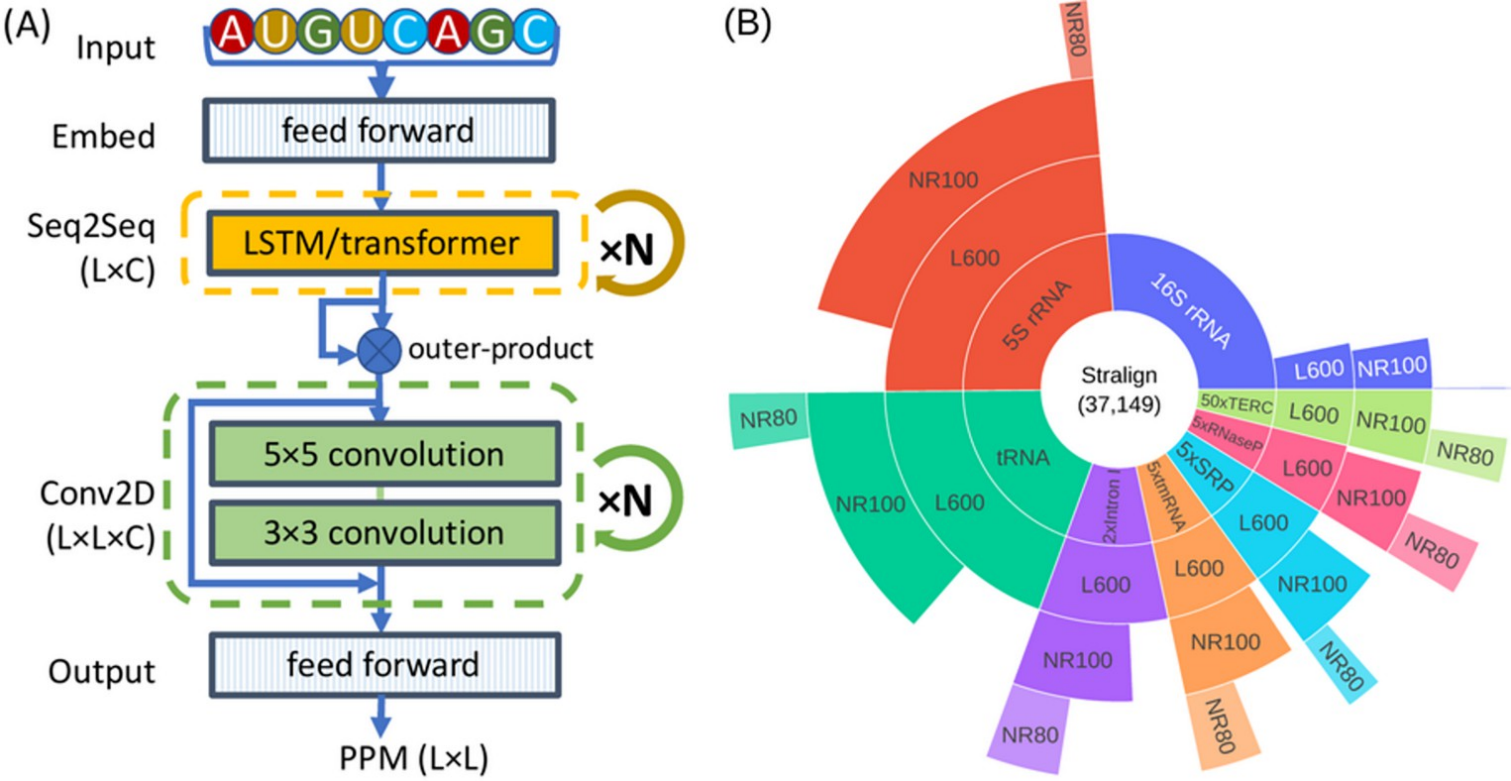
Illustrations of the SeqFold2D network and the Stralign dataset. (A) The two-module architecture of the SeqFold2D models. An input RNA sequence of length L is first embedded via one-hot encoding and feed-forward layers to yield an L×C tensor. The first module consists of N blocks of either bidirectional Long-Short-Term-Memory (LSTM) or transformer encoders. The resultant L×C tensor is then transformed into the L×L×C pair representation via outer-product, before being fed to the second module of N blocks of residual 2D convolutional layers. The output block is made up of three feed-forward layers and predicts the PPM of dimension L×L. (B) The population distributions of eight RNA families at different sequence similarity levels for the Stralign dataset. The abbreviations are, rRNA: ribosomal RNA, tRNA: transfer RNA, Intron I: group I intron, tmRNA: transfer messenger RNA, SRP: signal recognition particle, and TERC: telomerase RNA component. The innermost ring shows the original Stralign dataset with a total of 37,149 sequences, noting that the five under-represented families (counter-clockwise from Intron I to TERC) are scaled up for visibility and the multiplier N is shown as “N×” in the label (see Fig A in S1 Text for the unscaled version). The L600 ring is after removing sequences longer than 600; the NR100 ring shows the cross-sequence level; and the NR80 ring shows the cross-cluster level. Note that the 16S rRNA NR80 has only 50 sequences and is barely visible.

Two key ingredients of our study are the types of DL network and the distributions of the seen and unseen datasets. We reason that, given an RNA sequence, each nucleotide first explores all its intra-molecular contexts and then engages in the dynamic process of local paring and unpairing before arriving at the most stable configuration. A two-module architecture is thus chosen to capture both interactions at the sequence and pair levels. As shown in Fig 1A, our network, named SeqFold2D, mainly comprises a Seq2Seq and a Conv2D module flanked by the input and output blocks. To delineate sequence distributions, we devise three levels of sequence similarity between the seen and unseen datasets as follows. The first level only requires no identical sequences between the seen and unseen sets, i.e., the cross-sequence level. The second level further stipulates that all sequences are below 80% in identity (filtered with CD-HIT-EST [37]), referred to as the cross-cluster level. The third level is the most stringent by having the seen and unseen sequences from different RNA families, named as the cross-family level. Stralign [38] and ArchiveII [39] with RNA family information readily available are the main datasets for this study and bpRNA is also used for benchmarking. Fig 1B shows the distributions at different similarity levels of the Stralign dataset, noting vast redundancy in the original dataset that must be considered when developing learning-based models.

Sequences for each level of similarity comprise three subsets: training (TR), validation (VL), and test (TS) sets. The TR set is used to train model parameters and the VL set is used to optimize hyperparameters such as learning rate. Both TR and VL sets are thus the seen set and TS is the unseen. Model performances on the subsets can all be different, usually with the best for TR and the worst for TS. To take a closer look into model behaviors, we further distinguish two different performance gaps. The first is between the TR and VL sets that are always randomly split from the same distribution in this study. As such, if a model is learning the true distribution represented by the TR set, the same performance is expected on the VL set. Conversely, if a model learns additional spurious patterns of the TR set, TR-VL variances would emerge. TR-VL variances are thus indicators for model overfitting. The second gap is between the TR and TS sets that may or may not have the same sequence distributions. TR-TS variances, also known as generalization gaps, thus reflect model generalizability at the specific sequence similarity level.

### Cross-sequence study: Excellent capacity and generalizability of *de novo* DL models

**Fig 2:**
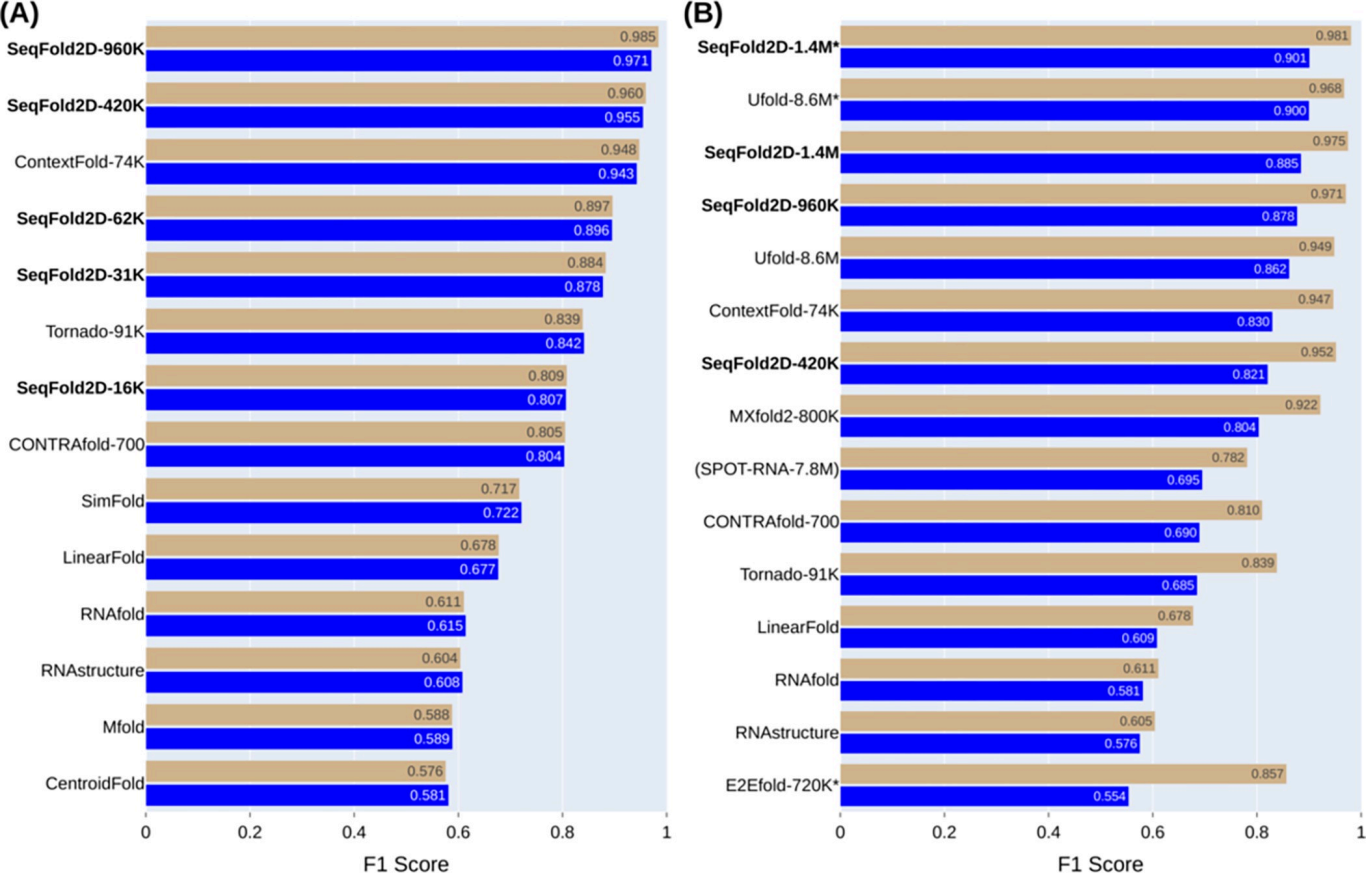
The mean F1 scores on the TR (tan) and TS (blue) sets by SeqFold2D and selected DL and traditional models in two different dataset setups. (A) Both TR and TS sets are from Stralign NR100. (B) TR from Stralign NR100 and TS from ArchiveII NR100.The models are sorted by their TS F1 scores. The names for the learning-based models are appended with the number of parameters and the trailing asterisk indicates the use of post-processing. At the end of each bar shows the F1 value. All learning-based models except SPOT-RNA are re-trained.

We first use the Stralign NR100 dataset (Stral-NR100 in short) to train and test SeqFold2D models of various sizes at the cross-sequence level. The TR, VL, and TS sets are randomly split from Stral-NR100 at the ratios of 70%, 15%, and 15%, respectively. By varying the number of blocks (N) and the channel size (C) shown in Fig 1A, we gradually increase the number of parameters from ~16K (N = 1, C = 16) to ~960K (N = 4, C = 64). Fig 2A shows the F1 scores on TR and TS from five SeqFold2D models and selected traditional models. Evident from Fig 2A, the performances of the SeqFold2D models increase steadily for both TR and TS as the model size increases. The smallest SeqFold2D-16K model achieves F1 scores ~0.8 and the largest SeqFold2D-960K attains nearly perfect performances (F1~0.985). Physics-based models give much lower but still decent scores in comparison. Three traditional machine learning (ML) models (ContextFold [40], CONTRAfold [41], and Tornado [42]) are shown with re-trained parameters that outperform their default values, noting that non-canonical base pairs and pseudoknots are removed from all datasets for the ML models, as well as for MXfold2 and physics-based models throughout this work. Our re-training efforts however failed to reproduce the original performances for existing *de novo* DL models and we will only discuss them when meaningful comparisons can be made. Interestingly, the ML models perform comparably with SeqFold2D models of similar sizes, indicating the general ease for learning-based models presented by the cross-sequence level.

Moreover, the TR-TS generalization gaps are negligible for the SeqFold2D models of sizes up to 420K parameters, as well as the three traditional ML models. A slight drop (~1.5%) in the F1 score can be spotted for the SeqFold2D-960K model (the top bars in Fig 2A). As the F1-score distributions are non-Gaussian, we apply the Kolmogorov-Smirnov (KS) test and find the TR-TS variance to be indeed statistically significant for the 960K model (P-value around 3e-6) but insignificant for all other SeqFold2D models (P-values greater than 0.1). Nonetheless, the TR-TS gaps are small and the TR-VL variances are verified to show similar behaviors (Fig H in S1 Text). Altogether, the SeqFold2D models demonstrate excellent learning capacity and generalization power at the cross-sequence level (e.g., F1~0.97 on the TS set by SeqFold2D-960K).

In order to compare with other DL models, we next follow the same dataset setup as used by MXfold2 and Ufold: Stral-NR100 as the TR and VL sets and ArchiveII-NR100 (Archi-NR100 in short) as TS. Fig 2B shows the TR and TS F1 scores from selected DL and traditional models, noting that the only available SPOT-RNA model was trained with the bpRNA dataset and is shown for reference only. The DL models, SeqFold2D included, are the best performers by rather large margins. The traditional ML models, while inferior to the DL models, show very competitive performances. Notably, both E2Efold and Ufold further post-process predictions by excluding non-canonical base pairs and sharp turns and enforcing sparsity through iterative refinement. We examined the effectiveness of such post-processing by comparing Ufold with and without post-processing, shown as Ufold-8.6M* and Ufold-8.6M, respectively. The post-processing indeed leads to considerable gains, ~2% for Stral-NR100 (TR) and ~4% for Archi-NR100 (TS). We then experimented with the same post-processing for the SeqFold2D-1.4M* model and realized similar gains, e.g., yielding the best F1 scores for both Stral-NR100 (0.981) and Archi-NR100 (0.901) among all models. However, such post-processing is subject to biases that may be inconsistent with the training and test data or true secondary structures. We therefore did not use post-processing for all other SeqFold2D models. Overall, the SeqFold2D models show to compare favorably against other *de novo* DL models for both datasets, albeit with fewer parameters.

Another marked trend in Fig 2B is that all DL and ML models, with or without post-processing, exhibit significant TR-TS generalization gaps with falloffs ranging from 8% to nearly 40%. However, we observed very little TR-VL variances for the SeqFold2D models (Fig I in S1 Text) and also carried out five-fold cross-validation to rule out the likelihood of fortuitous TR-VL splitting. Therefore, at least SeqFold2D models are not overfitting TR over VL but faithfully describing the entire Stral-NR100 distribution. Different distributions between Stral-NR100 and Archi-NR100 are thus left as the most plausible cause for the observed TR-TS gaps. On the whole, Stral-NR100 and Archi-NR100 have nearly identical RNA families (the only exception is 23S rRNA in Archi-NR100 only, but at a mere 0.6%), though with very different population shares of RNA families (Fig E in S1 Text). This motivated us to examine RNA family-specific performances, reasoning that Archi-NR100 may happen to have higher fractions of more difficult RNA families.

**Fig 3:**
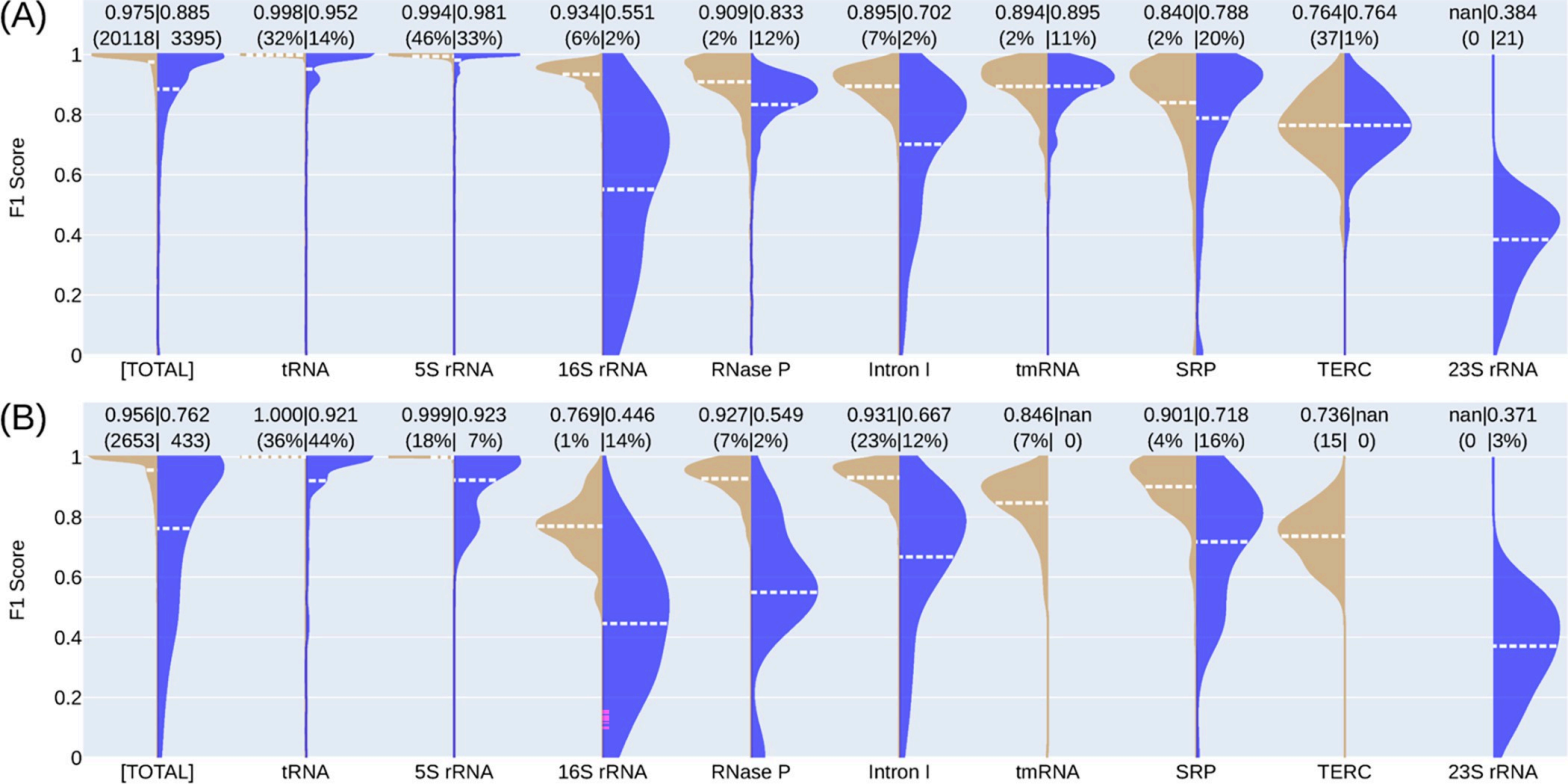
Illustrations of the TR-TS gaps of the SeqFold2D-1.4M model. (A) Stral-NR100 as TR and Archi-NR100 as TS. (B) Stral-NR80 as TR and Archi-Stral-NR80 as TS. The first pair of violins shows the F1 scores for the entire TR (left, tan) and TS (right, blue) set and the following pairs show the scores for each RNA family. Averaged scores are shown as dashed lines (white) and at the very top. The parentheses above show the sequence counts in numbers (for the entire set or families with <1% share) or in percentages (for families with >1% share). The families existing in one set only are shown as “nan” for the other set, e.g., 23S rRNA in Archi-NR100 only.

Fig 3A shows the TR vs. TS F1 scores per RNA family for the SeqFold2D-1.4M model. We indeed observe wide-ranging family-specific performances, for example, F1~0.998 for tRNA and F1~0.764 for telomerase RNA (TERC). The general trend is the higher scores for the more populous families. Contrary to the expectation of generalizable intra-family performances, large TR-TS gaps are observed for most families, with the largest ~41% for 16S rRNA and a significant ~5% for tRNA. It should be noted that tmRNA and TERC show almost no differences because their sequences are highly redundant between Stral-NR100 and Archi-NR100. We further verified that another DL model (Ufold) manifests the same qualitative family-wise performance gaps with or without post-processing (Fig J in S1 Text). Again, no significant TR-VL variances are observed (Fig K in S1 Text), ruling out the possibility that some RNA families are overfit and some are not. Altogether, these lead to a somewhat surprising observation that the *de novo* DL models are not guaranteed to generalize within the same RNA family type that is supposedly made up of closely related sequences.

### Cross-cluster study: Degraded performance and generalizability

The distributions within the same family can still be highly uneven when only identical sequences are removed at the cross-sequence level. One way of mitigation is to cluster similar sequences and remove all but one redundant sequence from each cluster. We applied this procedure using CD-HIT-EST with the identity cutoff of 80%, the lowest allowed, and obtained non-redundant Stral-NR80 (3,122 RNAs) and Archi-Stral-NR80 (433 RNAs) sets for this cross-cluster study. Out of curiosity, we verified that all inter-family sequences are below 80% identity. With Stral-NR80 as the seen (TR and VL) and Archi-Stral-NR80 as the unseen (TS), several SeqFold2D models of sizes 400K-1.4M were trained. As expected, model performances exhibit broad changes at both ensemble and family-specific levels. On the whole, all SeqFold2D models yield noticeably lower F1 scores on both seen and unseen datasets (Figs 3B and S1 Text) compared with the cross-sequence study (Fig 3A). This can be attributed to the fact that the removed sequences are redundant and presumably well-fitted. For each RNA family, its performance gain/loss is generally correlated with the increase/decrease of its population share in the dataset (Fig 3A vs. 3B), consistent with the effects of observation bias on learning-based models. Given that the similarity-based de-redundancy via CD-HIT-EST dramatically reduces the data size (84% reduction from Stral-NR100 to Stral-NR80), the drops in model performances may also be explained solely by the loss of training data. We then trained the SeqFold2D-420K model across a broad range of data sizes by random sampling down to 520 sequences (2.6% of Stral-NR100). Indeed, the random downsizing decreases both TR and VL performances (Fig I in S1 Text), as expected from the removal of similar sequences. When compared at the same data size of 3,122 total, the random-sampling approach gives significantly better performances—0.956 vs. 0.929 for TR and 0.939 vs. 0.905 for VL—than the similarity-based de-redundancy method. We attribute this to the fact that the latter is more effective in removing similar sequences that are more likely well-fitted.

Crucially, the TR-TS generalization gaps increase at both ensemble and family-specific levels for all SeqFold2D models, as shown in Figs 3B and L and M in S1 Text. Retraining five other DL and ML models with the same datasets reveals similar trends in both performances and generalizability (Fig N in S1 Text). Compared with the cross-sequence study with Stral-NR100, the DL or ML models with fewer parameters typically suffer greater drops in absolute performance, while the larger DL or ML models suffer greater drops in TR-TS generalization, signifying an inverse correlation between performance and generalization. We like to note that several strategies have been explored to improve the generalizability of SeqFold2D models, but to rather limited effects. We mainly experimented with regularization methods such as dropout and weight decay rates; see Fig O in S1 Text for a scan of dropout rates. SeqFold2D models are trained until the VL F1 score starts decreasing. One alternative is to stop training as soon as the TR-VL variance increases, which however leads to rather unsatisfactory performances (e.g., F1 score below 0.8). Above all, DL and ML models exhibit consistent behaviors, establishing that learning-based models struggle to attain both superior performance and generalizability at the cross-cluster level.

To further verify that the observations are not dataset-specific, we carry out another cross-cluster study with the bpRNA TR0, VL0, and TS0 datasets (all below 80% identity filtered with CD-HIT), for which SPOT-RNA, MXfold2, and Ufold provided model benchmarks or pre-trained parameters. Unlike two separate sources for Stral-NR80 and Archi-Stral-NR80, the three bpRNA subsets are random splits of the same parent dataset and offer a more standard evaluation, though without readily available RNA family information. Overall, as shown in Fig P in S1 Text, all models (DL, ML, and physics-based) exhibit much poorer performances than with Stral-NR80, which may be attributed to the much larger size of the bpRNA subsets (totaling 13,419 vs. 3,122 for Stral-NR80) presumably with much broader distributions. Consistent with the dichotomy between performance and generalizability, significant TR0-TS0 gaps are only observed for the high performing models achieving TR0 and TS0 F1 scores over 0.6. Among all models, the SeqFold2D model with 3.5M parameters attains the best F1 scores of 0.903 and 0.665 for TR0 and TS0, respectively, both the highest reported to date. However, SeqFold2D-3.5M also gives the largest TR0-TS0 gap. We also experimented with various regularization methods to reduce the generalization gap but met similar difficulties as in the case of Stral-NR80; see Fig Q in S1 Text for the optimization of dropout and related discussion. Altogether, we observe that the low TS0 scores are not caused by the learning capacity of DL models (e.g., TR0 F1~0.903 for SeqFold2d-3.5M) but by the poor generalizability at the cross-cluster level.

### Cross-family study: Inability to generalize over unseen RNA families

**Fig 4:**
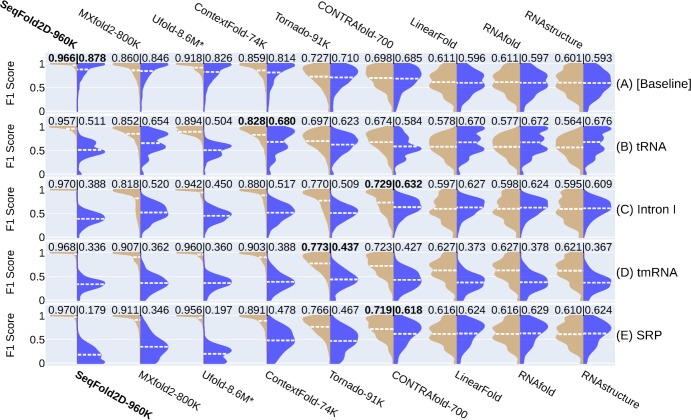
Illustrations of the TR (left, tan) vs. TS (right, blue) performances for selected learning and physics-based models at the cross-family level with the Strive-NR80 dataset. For each cross-family study, one RNA family is held out as the TS set and the rest eight families are used for model development (TR and VL). Each panel/row here shows one such study labelled by the TS family name (B-E), while the first panel, (A) [Baseline], shows a baseline study with randomly splits of all families for the TR, VL, and TS subsets. Panel A thus is *de facto* a cross-cluster study with all subsets derived from the same parent dataset. For each panel, the average TR and TS scores are shown at the top and highlighted for the learning-based model with the highest TS score (physics-based models excluded). All learning-based models are retrained with the numbers of parameters shown after names. It should be noted that, despite our best re-training efforts, the scores of MXfold2 and Ufold should be viewed as guides only as we are unable to match their reported performances when using the same datasets. Still, given the inverse correlation between TR and TS performances, their TR-TS gaps are expected to be under-estimates.

An even more stringent examination of generalizability is for the seen and unseen sets to share no RNA families in common, i.e., a cross-family test. All learning-based models are expected to show even worse generalization than in the cross-cluster test. One way is to choose a single RNA family as the unseen and all other families as the seen. A very recent study reported a similar cross-family survey of the generalizability with the smaller ArchiveII set without removing redundant sequences [32]. To increase coverage and mitigate redundancy-related biases, here we combine the Stralign and ArchiveII datasets into one Strive dataset with a total of nine families (Fig E in S1 Text) and further obtain its non-redundant set below 80% identity, Strive-NR80. By excluding one RNA family at a time, Strive-NR80 provides nine cross-family datasets used to train SeqFold2D-960K and five other DL or ML models. Fig 4 compares the TR vs. TS scores from several cross-family studies (see Fig R in S1 Text for all nine). The cross-cluster study with Strive-NR80 (i.e., random splits of all families to get TR, VL, and TS) is also shown in Fig 4A, where we observe excellent performances (e.g., TR and TS scores >0.8) and modest generalizability. However, for all nine cross-family studies, all DL and ML models show poor TS performances and substantial TR-TS gaps, establishing the inability of learning-based models to generalize over unseen RNA families.

**Fig 5:**
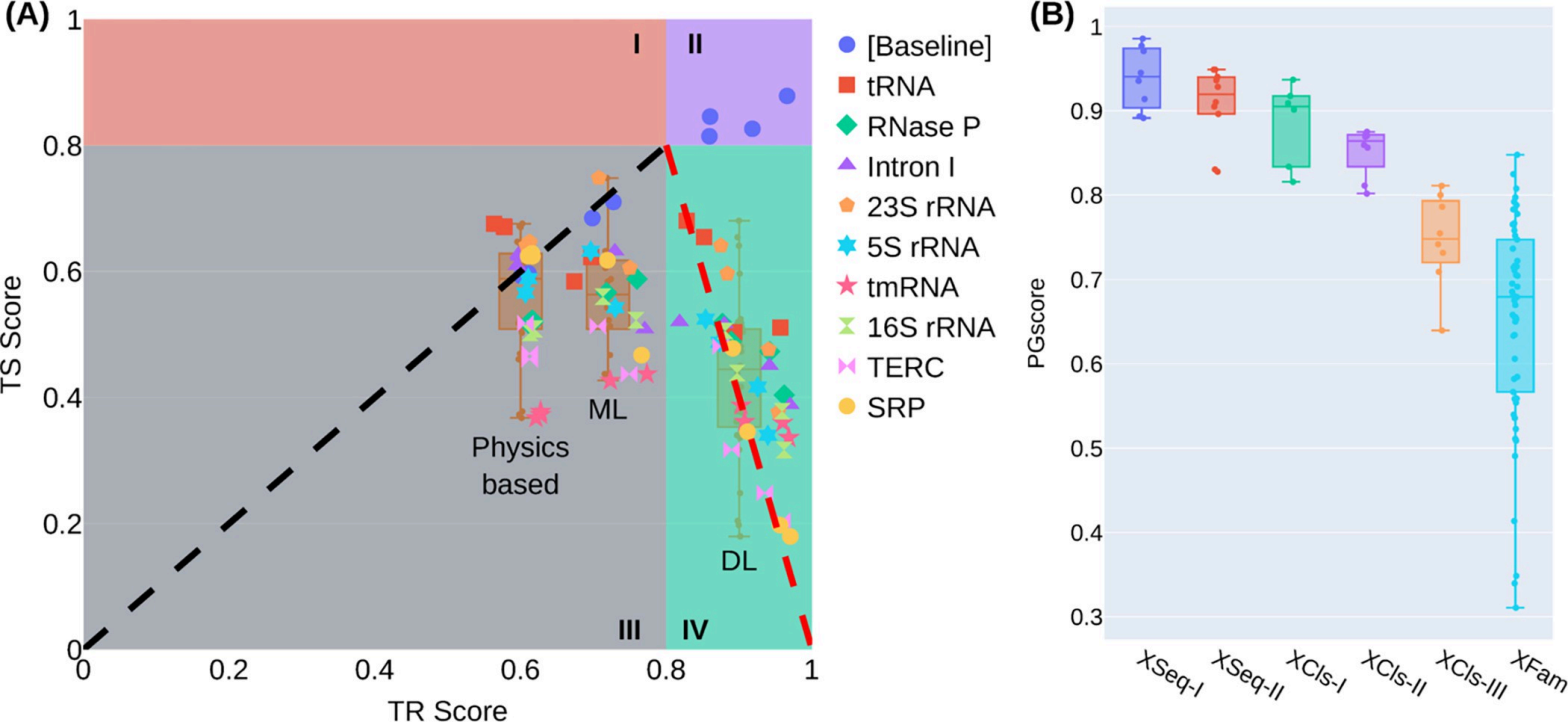
Illustrations of the cross-family F1 scores and the PGscore distributions for all studies. (A) The TS vs. TR F1 scores of the baseline cross-cluster study ([Baseline]) and all nine cross-family studies (labelled by the TS family name) with Strive-NR80. Four zones (I-IV) are delineated for easy reference. The diagonal line in zone III denotes the line of zero TR-TS gap, i.e., TR = TS. The dash line in zone IV is a guide to the eye only. The cross-family TS scores of the three groups of models (physics-based, ML, and DL) are shown in three respective boxplots as annotated. (B) Boxplots of the PGscores from all learning-based models for each study at the specific TR-TS similarity level. The studies are, XSeq-I: the cross-sequence study with Stral-NR100, XSeq-II: cross-sequence with Stral-NR100 and Archi-NR100, XCls-I: cross-cluster with Strive-NR80, XCls-II: cross-cluster with Stral-NR80 and Archi-Stral-NR80, XCls-III: cross-cluster with bpRNA, XFam: all nine cross-family studies with Strive-NR80. The learning-based models included for each study are shown in Figs T-U in S1 Text.

Comparisons between all cross-family TR and TS performances, shown in Fig 5A, reveal clustering of the three model groups: physics-based, ML, and DL. The physics-based models typically show not only the least TR-TS gaps but also the highest TS scores, emerging as the best group at the cross-family level. The ML models show significantly higher TR scores and slightly lower TS scores than the physics-based models, while the DL models generally give the highest TR and lowest TS scores and the largest TR-TS gaps. We further examine a more extreme case of the cross-family test, in which one family is used to train and all the rest as test. As shown in Fig S in S1 Text for the case of tRNA as TR, the SeqFold2D-400K model fails completely on all other RNA families, yielding TS F1 scores ranging from 0.03 to 0.1. Hence no further experimentation was carried out. All taken together, the cross-family studies offer salient examples of the limitations of learning-based models that lead to poor and erratic performances over out-of-distribution sequences, bringing dire uncertainties to their real-world applications.

### Dichotomy between training performance and generalizability

Our studies so far indicate that, for all learning-based models, training performance is primarily determined by model capacity and, to a lesser extent, sequence redundancy in the TR set. TR-TS generalization gap depends on the sequence similarity between TR and TS datasets and, evidently, on training performance as well. The latter is especially acute at the cross-family level. As summarized in Fig 5A, it is remarkably robust that the performances on the TR set inversely correlate with that on the TS set across DL and ML models, for each cross-family study or as a whole. The general trend is that larger models attain better TR performances, which however lead to worse TS performances and larger generalization gaps. The TS falloff is the steepest for the DL models (zone IV) despite the common uses of model regularization. Therefore, training performance appears to be antagonistic to generalizability at low sequence similarity levels, attesting to the challenges of data-driven statistical learning.

Consequently, neither (training) performance nor generalizability is a reliable measure of prediction quality for new sequences with unknown distributions. Superior performance is preferred if the new sequences are similar to the seen set, while generalizability is favored for out-of-distribution sequences. Determining the similarity with clustering or sequence alignment tools would defeat the purpose of *de novo* prediction without homology search. This creates a conundrum for developing and evaluating *de novo* models. To this end, we devise a benchmark score, PGscore, by combining performance (P) and generalizability (G). PGscore is defined as PGscore = 2PG/(P+G), where P is the TR F1 score and G is the ratio between the TS and TR scores. Notably, P or G can take on any value between zero and one (G can be greater than one in theory but neglected herein). For example, P would be virtually zero for a random predictor and one for a perfect memorizer; G would be nearly one and zero, correspondingly. As such, learning-based models can be developed to give any P or G value individually, but not any specific P and G pair. Hence, PGscore provides a balanced measure for both metrics. The PGscores for all studies in this work are shown in Fig 5B where a strong dependence on the seen vs. unseen sequence similarity is observed. Practically, PGscore can be used to rank models. As illustrated in Figs T-U in S1 Text, DL models always perform the best at the cross-sequence and cross-cluster levels if the TR, VL, TS sets are derived from the same parent distribution, while ML and physics-based models outperform DL models at the cross-family level. Such rankings can be useful for selecting the best model for a specific task, especially when cross-family sequence/structures are among the prediction targets.

Nonetheless, the challenge for developing data-agonistic *de novo* models persists. One practical approach is to output the confidence levels of model predictions, like the pTMscore by AlphaFold2 [43]. We have first exploited the fact that each predicted PPM_ij_ is a probability itself and has an apparent variance of ${PPM}_{ij}(1-{PPM}_{ij})$ under the two-state assumption. One can derive the variance of the resultant soft F1 score which is a function of the predicted PPM and the ground truth. One example of such estimated variances vs. the actual F1 scores is given in Fig V in S1 Text. While clear negative correlations are observed when the F1 scores are greater than 0.8, the variances show no or even reversed correlations when the F1 scores fall below 0.8, rendering it completely uninformative of model confidence. Additionally, we experimented with the addition of a third module to predict the F1 score which however shows poor cross-family generalization despite excellent training performances. Generalizability again appears to be a major roadblock and more efforts are needed.

### Pairwise alignments quantify the roles of sequence and structure similarity in generalizability

Here we first take advantage of the quite unique facility offered by biological sequences to obtain sequence similarity via pairwise sequence alignment (PSA), enabling us to quantify the correlation between sequence similarity and generalizability. To have a broad F1 score distribution of the TR and TS sets, we analyze the SeqFold2D-960K model with Stral-NR80 as the seen and Archi-Stral-NR80 as the unseen datasets, as detailed below.

For every unseen sequence, it is first aligned against every sequence in the seen set (3122 in total) to produce 3122 PSAs. A percentage sequence identity (PSI) score is then calculated from each PSA as the number of identical aligning nucleotides divided by the average length of the sequence pair. Note that we experimented with alternative PSI definitions (e.g., using the shorter length of the sequence pair) and observed qualitatively consistent results. As the seen set consists of highly diverse sequences across eight RNA families, the 3122 PSI scores are broadly distributed. Now with the pairwise PSI values of the unseen sequence to the entire seen set, the question we next ask is, can we use the subset of seen sequences above a certain PSI value to inform the model performance for the unseen sequence? One extreme case would be setting the PSI threshold to 1.0 (i.e., using a seen sequence identical to the unseen), where the same performances are granted. Lowering the PSI threshold gradually includes more dissimilar sequences from the seen set and the informative power is expected to decrease. As such, the dependence of the informative power on the PSI threshold may provide quantitative insights into the model generalizability. Specifically, for the subset of seen sequences above a given PSI threshold, we average their F1 scores weighted by the PSI values to obtain the F1-seen score as the surrogate of their informative power. The F1-seen score is then compared with the actual F1 score for the unseen sequence (F1-unseen) at different PSI thresholds.

**Fig 6:**
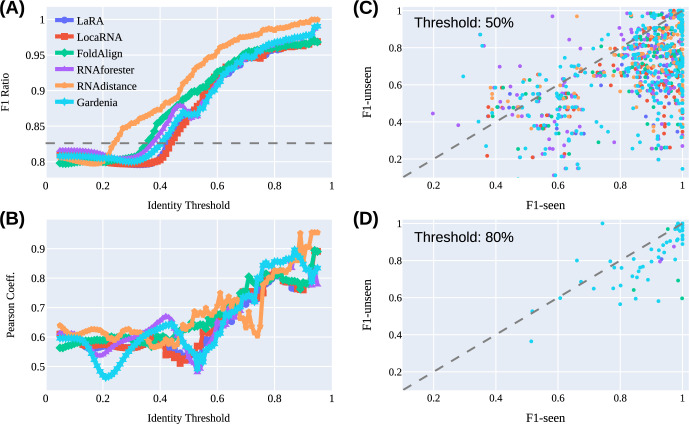
Illustrations of the F1-unseen vs. F1-seen correlations of the SeqFold2D-960K model. **Each PSA or PSSA program is shown in the same color in all panels.** (A) The F1-unseen over F1-seen ratio as a function of the PSI or PSSI threshold. The horizontal dashed line marks the F1 ratio between the entire unseen and seen datasets. (B) The PCC value as a function of the identity threshold. (C) The distributions of the F1-unseen and F1-seen scores at the nominal PSI or PSSI threshold of 50%. (D) The distributions of the F1-unseen and F1-seen scores at the identity threshold of 80%. It is common to find no seen sequences above high thresholds for an unseen sequence, leading to many null F1-seen values that are absent in (D).

The entire process described above is repeated for all sequences in the Archi-Stral-NR80 dataset (433 total). As a result, at each PSI threshold, we obtain 433 pairs of F1-seen and F1-unseen values and examine their statistical correlations with rubrics such as the Pearson correlation coefficient (PCC). In order to rule out coincidental statistics, three different PSA programs were chosen largely for speed considerations: Foldalign [44], LaRA2 [45], and LocaRNA [46], among others such as Dynalign [47] and RNAmountAlign [48]. Note that all three PSA programs carry out *de facto* sequence-structure alignments via simultaneous folding and aligning and that we categorize them as PSA programs because the ground-truth secondary structures are not provided as inputs. Fig 6A and 6B show the F1-unseen/F1-seen ratios and PCC values as a function of the PSI threshold. Representative distributions of F1-seen and F1-unseen values are shown in Fig 6C and 6D. It is worth noting that all three programs find seen sequences with PSI higher than 0.8, somewhat unexpected because of the 80% redundancy removal by CD-HIT. We speculate that this is caused by algorithmic differences as CD-HIT employs a greedy incremental clustering method that first estimates sequence similarity via short-word counting (word length of five used). This may lead to missed matches by CD-HIT as the PSA programs always carry out actual alignment. It is thus suggested that additional de-redundancy steps (e.g., with BlastN [49] and Infernal [50]) may be warranted for situations where sequence non-redundancies are critical. Our analysis here is not significantly affected by low levels of highly similar sequences and it is in fact reassuring that the F1 ratio and PCC both approach the asymptotic value of 1.0 as the PSI threshold approaches 1.0. No further de-redundancy was thus carried out.

The main observation in Fig 6 is the rapid declines of both F1 ratio and PCC with the decrease of sequence similarity, consistently shown by all three PSA programs. In the direction of decreasing PSI thresholds, both F1 ratio and PCC start out in the high 90%s, affirming the excellent generalizability over non-identical but similar sequences observed in the cross-sequence study. F1 ratio and PCC then quickly drop as the PSI threshold is lowered, indicating a fast decay of generalizability over increasingly dissimilar sequences. Continuing down, both values appear to plateau: the F1 ratio falls slightly below its ensemble average around PSI~0.4 while PCC flattens out as early as PSI ~0.6. The same analysis is done for the five other DL and ML models that show qualitatively consistent behaviors (Figs W-AA in S1 Text), particularly the rather robust plateauing PSI thresholds (0.4 for F1 ratio and 0.6 for PCC). Interestingly, the physics-based models (Figs BB-DD in S1 Text) exhibit similar PCC trends, suggesting that the PCC curve largely reflects the intrinsic RNA sequence-secondary structure conservation, i.e., little structure resemblance below PSI~0.6. Therefore, we consider the F1 ratio more informative of model generalizability which quickly decreases upon lowering PSI and diminishes completely at PSI ~0.4. While the exact PSI transition point depends on the dataset, the PSI definition, etc., our analysis provides the first quantitative insights into how the generalizability of *de novo* models depends on the sequence similarity between the seen and unseen datasets.

Yet another unique facility offered by RNA is the ability to obtain structure similarity by aligning secondary structures. This raises an interesting question whether *de novo* DL and ML models learn, besides sequence-structure correlations, some specific patterns of the structure space as well, e.g., PPM is always very sparse with stems depicted as cross-diagonal lines. We then carried out pairwise secondary structure alignment (PSSA) between the seen and unseen sets with three PSSA programs (RNAforester [36], RNAdistance [36], and Gardenia [51]) and obtain the percentage secondary structure identity (PSSI) (identical aligning bases are counted). The results are also shown in Figs 6 and W-DD in S1 Text, revealing qualitative agreements with the PSA analysis, as well as some quantitative differences noted below.

RNAdistance shows to yield PSSI values carrying the most informative power by giving the highest F1 ratios, though this is only observed for the larger DL models (SeqFold2D and Ufold). It is also the only program that uses secondary structures only for alignment (i.e., no sequence inputs), suggesting that the DL models likely learn some patterns of the structure space. This observation motivates us to investigate further the dependencies of model performance on the similarities in RNA sequence and structure. Taking FoldAlign vs. RNAdistance as an example, we first examine their respective PSI and PSSI scores for all RNA pairs between the unseen and seen datasets. Compared to FoldAlign with an average score of 0.126, RNAdistance obtains a higher average of 0.171, slightly lower scores for highly similar pairs, and higher scores for dissimilar pairs, as shown in Fig EE in S1 Text. Since the performance on an unseen sequence is largely influenced by its most similar sequences in the seen set, we extract only the maximum PSI and PSSI scores for each unseen sequence. We then divide the unseen set into two groups of comparable sizes based on the ratio between the PSI and PSSI scores of each molecule. One group has PSI/PSSI ratios above 1.08 and the other has ratios below 1.08. As shown in Fig EE-B in S1 Text, the two groups span nearly the same sequence similarities, but have lower and higher structure similarities, respectively. As expected, the SeqFold2D-960K model performs substantially better on the group with higher structure similarity, at any sequence similarity level (Fig EE-C in S1 Text) and in aggregate (Fig EE-D in S1 Text). Similar performance gaps are observed from other learning-based models (see Figs FF-HH in S1 Text for Ufold, MXfold2, and ContextFold, respectively), whereas CONTRAfold again exhibits the smallest gap concomitant with the lowest F1 scores (Fig II in S1 Text). Non-negligible gaps are also observed from physics-based models (Figs JJ-LL in S1 Text), likely resulting from the limited size and unbalanced distribution of the datasets, though these gaps are much smaller. In all, the results suggest that learning-based models tend to memorize, to some extent, the structural space in training and exhibit observation-bias-like behaviors during inference.

It is however difficult to draw quantitative conclusions on the relative importance between sequence and structure similarities, as the two factors are highly correlated by the sequence-structure relationship. Furthermore, such analysis is inevitably influenced by the size, distribution, and consistency of the datasets used, exemplified by the noticeable gaps from the physics-based models aforementioned. Given the scarce and unbalanced nature of existing datasets, the approach of Flamm et al. [33] provides a very useful avenue to interrogate model behaviors in a comprehensive and consistent manner, in which training and test datasets of arbitrary sequences are generated randomly and their structures are predicted with a thermodynamic model, allowing precise diagnosis of model characteristics. Nonetheless, unless the training data maps all sequences into a single secondary structure, DL models are expected to learn features of both RNA sequences and structures, whose relative weights would depend on the dataset, model architecture, and training method.

Meanwhile, RNAforester and Gardenia show behaviors very similar to the PSA programs except for a hump in the F1 ratios around PSSI of 0.46. This hump however exists for all learning and physics-based models, hinting roots unrelated to learning. Examination of the unseen sequences with F1-unseen > F1-seen (i.e., above the diagonal line in Fig 6C) indicate diverse family distributions. As shown in Figs MM-NN in S1 Text for the examples of FoldAlign vs. RNAforester and FoldAlign vs. RNAdistance, the main difference between the PSA and PSSA programs appears to be their identity scores in the order of RNAforester ~ Gardenia > RNAdistance > all PSA programs. As F1-unseen values are invariant the likely cause of the hump is thus that the broader inclusion of seen sequences that are marginally distributed and have low F1 scores. It is worth noting that the mean inter-family PSI/PSSI values are typically less than 0.2 (Figs MM-NN in S1 Text), well below the observed threshold of 0.4 for significant informative power, corroborating the inability of learning-based models to generalize at the cross-family level. Thereupon, the PSSA analysis reinforces the PSA study and augments new quantitative perspectives of the relation between sequence/structure similarity and generalizability.

## Discussion

We set out to study the performance and generalizability of *de novo* DL models under varied sequence distributions. To this end, we design a series of SeqFold2D models of different sizes with a minimal two-module architecture. The SeqFold2D models exhibit excellent learning capacity by outperforming other DL, ML, and physics-based models on all training sets, despite often with much fewer parameters and without post-processing. Model generalizability, however, strongly depends on sequence similarity between the training and test sets. At the cross-sequence and cross-cluster levels, the SeqFold2D models show decent generalization and are the top performers on all test sets; the DL and ML models rank higher than physics-based models in general. On the other hand, the cross-family level presents a steep challenge for all learning-based models exhibiting worse test performances than physics-based models. Hence a dichotomy between (training) performance and generalization is observed, revealing the nature of statistical learning of *de novo* DL and ML models. Enabled by the abilities to align sequences and structures, we last quantify the relation between sequence/structure similarity and model generalizability, gaining unique insights into the inner workings of data-driven learning. Notably, structure similarity, though unknown for new sequences *a priori*, is poised to play a more important role than sequence similarity.

Meanwhile, our study can be extended in several directions including dataset coverage, performance analysis, and network training. The datasets used here only include non-coding RNA, patently missing coding RNA for which the majority of existing data are 1D base attributes rather than base pairing. It will be a valuable future addition. Conversely it would be useful to focus on high-resolution structures only (e.g., from the PDB database), though the number of such sequences is still small. Synthetic RNA sequences and structures utilized by Flamm et al. [33] would further provide useful channels to disentangle the roles of sequence and structure similarities. For analysis, the ensemble-averaged F1 score is used as the sole metric and it would be informative to examine the details of incorrect predictions and learn from them. Separating sequences by length or base pairs by distance would also provide new angles, particularly to examine the capacity to predict RNA sequences much longer than the training set. Moreover, network training can be further tuned to better balance performance and generalizability, for example, the PGscore may be used as the criterion for early stopping with VL used in place of TS. Lastly, it would also be highly desirable to understand how the *de novo* DL models predict secondary structures, using various techniques from the machine learning field [52].

Accurate, data-agnostic *de novo* models are still far from being attained. For example, no models have achieved decent performances on the bpRNA TS0 set, the highest F1 is ~0.665 by SeqFold2D-3.5M despite F1~0.903 on the TR0 set. The poor generalizability, as suggested by this work, is most likely caused by the spare sequence distribution of bpRNA (e.g., distant clusters of sequences). One solution is thus to increase the coverage and density of training structures. Experimental determinations of RNA secondary structures are however slow and costly [53] and high-throughput measurements such as PARS [54] and SHAPE [55] usually yield nucleotide-level activity profiles rather than base-pairing partners. Given that comparative sequence analysis is the main method of curation for large databases, renewed efforts with more sophisticated pipelines such as RNAcmap [56] and rMSA [57] could be viable. On the other hand, wide ranges of resources and techniques in the fields of biology and machine learning may be exploited, some of which are discussed below.

### Input enrichment

Inputs may be enriched beyond one-hot identifiers. Chemical and physical properties of the nucleotides can be embedded to capture their intrinsic features. Moreover, unsupervised pre-training of known RNA sequences with natural language models such as BERT [58] may offer a powerful pathway for representation learning, e.g., RNAcentral sequences [59] can be used to learn from all non-coding RNAs.

### Model design

Mechanisms of RNA structure and folding [3] can be better infused into the algorithms. First, one may quickly introduce learning biases in the form of auxiliary loss functions similar to the Physics-Informed Neural Networks (PINN) [60]. For example, the structural constraint of no base multiples can be enforced by adding a loss term $ReLU(\sum_{j=1}^{L}ThresholdedReLU({PPM}_{ij},0.5)-1.0)$ and sharp turns can be penalized through a masked loss of the diagonal elements. Another important aspect of RNA secondary structures is their topology [61]. One *posteriori* way is to increase the weights of loop opening and closing in the loss function. Lastly, DL networks can take up more sophisticated designs such as the EvoFormer module in Alphafold2 [43] that facilitates iterative exchanges between sequence and pair representations. First demonstrated by DeepFoldRNA [62], several recent works adopted EvoFormer for RNA tertiary structure prediction and reported state-of-the-art performances. However, no DL models are in the top four performers at the CASP15 RNA prediction contest that ended in Dec. 2022 (https://predictioncenter.org/casp15/zscores_RNA.cgi). One probable cause is the limited generalizability of DL models for out-of-distribution sequences even with advanced architectures and homologous sequences.

### Multi-task learning

Model outputs can go beyond base-pairing probability matrices. Additional structural properties can be predicted at the nucleotide level, such as nucleotide-wise solvent accessibility or activity profiles. Physical quantities at the sequence level such as free energies and melting temperatures may also be predicted. Moreover, it would be highly desirable for DL models to output an ensemble of competing structures in a meaningful way to recapitulate alternative structures of functional importance for RNA (and protein). No current DL models are designed for this task and the emergent generative DL may offer a path forward. In the absence of abundant structure data of this kind, the structure ensembles generated by physics-based models can serve as a testing ground.

In closing, RNA secondary structures confer important biochemical and physical features to all RNAs, coding or non-coding, and they often play critical roles in the biological functions of RNA molecules. The challenges of predicting RNA secondary structures *de novo* have inspired the development of numerous DL models that demonstrate unprecedented expressive power as well as stern reliance on broadly distributed training data. This study provides quantitative analyses of the model performance and generalizability in the context of different sequence similarities. And various pathways for future advances are discussed so as to catalyze the development of next-generation *de novo* DL models for RNA secondary structure prediction. Conversely, the sequence and structure alignment tools afforded by biological molecules may present unique opportunities for gaining deep insights into deep learning algorithms.

## Materials and methods

### Datasets

With the three sequence similarity levels in mind, we choose to primarily work with two recent datasets, RNA Stralign curated in 2017 [38] and ArchiveII curated in 2016 [39]. Both are medium-sized, comprehensive databases developed for secondary structure predictions. Compared with the only other larger collection (bpRNA curated in 2018 [19], also used in this study), Stralign and ArchiveII make family types readily accessible, facilitating intra- and inter-family examinations. Both datasets have been used by other DL models (e.g., E2Efold, MXfold2, and Ufold) with pre-trained parameters available. RNA sequences longer than 600 bases are excluded for consistency with other DL models. For the cross-sequence level, only duplicated sequences are deleted, yielding Stralign NR100 and ArchiveII NR100 with 20,118 and 3,395 sequences, respectively. CD-HIT is then used to remove sequences with over 80% identity, yielding the Stralign NR80 and ArchiveII NR80 datasets for the cross-cluster study. The ArchiveII NR80 sequences with over 80% identity to Stralign NR80 are further removed to give the ArchiveII-Stralign NR80 dataset. For the cross-family study, the Stralign and ArchiveII datasets are combined into the Strive dataset, which is then processed similarly to obtain its NR100 and NR80 subsets. More information on all datasets used is given in section 1, S1 Text.

### Network architecture

As shown in Fig 1A, our DL neural network, named SeqFold2D, comprises two main modules flanked by the input and output blocks. As the only input, each sequence of length L is first one-hot-encoded as an L×4 tensor and then stacked into its k-mer (k = 3) representation of L×12. The input block mixes the twelve channels into an L×C tensor with two feed-forward layers. The channel size C is then kept constant unless noted otherwise. The first main learning module is made up of N repeated blocks of bidirectional Long-Short-Term-Memory (LSTM) or transformer encoder layers to learn richer sequence representations, which are then transformed into 2D pair representations (L×L×C) via outer-product. The second learning module consists of the same number (N) of residual 2D convolutional layers. The output block consists of three feed-forward layers with the channel size C = 2 for the final layer. Softmax is then used to yield the continuous PPM of shape L×L. Non-linear activations (LeakyReLU or Swish) are applied before every operation with weights and biases, followed by layer normalizations and dropouts (0.2–0.42). The size of a specific SeqFold2D net is thus determined by two design variables, N (the number of blocks) and C (the channel size). For example, N = 1 and C = 16 gives ~16K parameters and N = 4 and C = 64 gives ~960K parameters.

### Performance metrics and benchmarking

We use the F1 score as the main metric for evaluating model performances [63]. It is the harmonic mean of Precision and Recall and defined as $\frac{2\times Precision\times Recall}{Precison+Recall}=\frac{2\times TP}{2\times TP+FP+FN}=\frac{2\times TP}{L^{2}+TP-TN}$, where Precision is TP/(TP+FP), Recall TP/(TP+FN), TP the number of true positives, FP false positives, FN false negatives, and TN true negatives. The predicted and ground-truth PPMs for a sequence are evaluated element by element for the confusion matrix without allowance for the displacement of base pairs by one nucleotide. The mean F1 score of a dataset is calculated by treating every sequence’s F1 score equally rather than averaging the mean F1 scores of constituent families. In addition to several *de novo* DL models, we further benchmark the SeqFold2D models against the following traditional algorithms: Mfold/UNAfold [64], RNAfold [36], RNAstructure [35], LinearFold [65], SimFold [66], CONTRAfold [41], ContextFold [40], and Centroidfold [67]. Note that we categorize machine-learning models as traditional algorithms for wording simplicity.

### Model development and evaluation of overfitting and generalizability

All SeqFold2D models were implemented with the Paddle framework (https://github.com/PaddlePaddle/Paddle) and trained with the AdamW optimizer in two stages. The first stage uses the binary cross-entropy (CE) loss between the predicted PPM and the ground truth with equal weights for positive and negative labels. Once the CE loss plateaus, the second stage is invoked with the soft F1-score loss used by E2Efold and others. For hyperparameter tuning, we carried out limited manual searches for the learning rate, batch size, and dropout rate. More details are described in section 2, S1 Text.

## Supporting information

S1 Text **Fig A. The population distributions of RNA families in the Stralign dataset at different sequence redundancy levels.** This is an unscaled version of Fig 1 in the main text and the TERC (telomerase RNA) population is too small and barely visible. The innermost ring shows the original Stralign dataset. The L600 ring is after removing lengths over 600; the NR100 rings shows the cross-sequence level; and the NR80 ring shows the cross-cluster level. **Fig B. The length distribution of each RNA family in the Stralign NR100 (A) and Stralign-NR80 (B) datasets shown in the same order and color as in Fig A in S1 Text.** The number of each family type and its percentage in the parent dataset are shown in the legend. **Fig C. The population distributions of all RNA families of the RNA ArchiveII dataset at different sequence redundancy levels.** Two versions for the same underlying datasets are shown, the unscaled version (A) and the scaled version (B) for visibility of the underrepresented families that are scaled up by the multiplier N shown in the label. With the same notations as used in Fig A in S1 Text, the innermost ring shows the relative populations of the RNA families in the original ArchiveII dataset. The L600 rings are after removing lengths over 600; the NR100 rings show the cross-sequence levels; and the NR80 rings show the cross-cluster levels. Note that group II intron, labelled as Intron II, all have lengths longer than 600 and are thus absent in the NR100 and NR80 datasets. **Fig D. The length distributions of all RNA families in the ArchiveII NR100 (A) and NR80 (B) datasets.** The order of the RNA families shown follows that of the Stralign datasets in Fig B in S1 Text to facilitate comparison, rather than by the order of population as in Fig C in S1 Text. **Fig E. The distributions of RNA families in Strive NR100 (A) and Strive NR80 (B).** Together shown are the corresponding Stralign and ArchiveII sets for comparison. The order of RNA families is sorted by the family abundance summing over all three datasets. **Fig F. The population distributions (left panel) and length distributions (right panel) of the sequences grouped by their sources in the bpRNA TR0 and VL0 datasets.** The x-axis range is shown up to 600 nucleotides for easy comparisons with Figs B and D in S1 Text, while the actual sequences are all shorter than 500 nucleotides. The TS0 set has essentially the same source and length distributions and thus not shown separately. **Fig G. The length distribution of the bpRNA-NEW dataset.** It is qualitatively similar to the RFAM length distributions of the bpRNA TR0 and VL0 sets shown in Fig F in S1 Text. **Fig H. The F1 scores of the training (left, tan) and validation (right, violet) sets for several SeqFold2D models developed with the Stralign NR100 (Stral-NR100) dataset randomly split into three subsets: training (TR), validation (VL), and test (TS).** The averaged F1 scores are shown at the top and also as dashed lines (white) within the corresponding violin plots (often too narrow to be spotted). Very little TR-VL variances are observed, indicating that the SeqFold2D models are learning the distribution of the entire Stral-NR100 dataset while being trained on the TR subset of the distribution. Note that the F1 scores were saved during training and all dropout layers were active for the TR set but not for the VL set. These make the F1 scores shown here slightly lower than the values computed without dropout. **Fig I. The F1 scores of the training (left, tan) and validation (right, violet) sets for the SeqFold2D models developed with the Stralign NR100 (Stral-NR100) dataset randomly split into two subsets only: training (TR) and validation (VL).** (A) The performances of SeqFold2D models of different sizes as labelled. Here the entire Stral-NR100 dataset (20,118 sequences) are used for TR and VL. The test set is the ArchiveII NR100 dataset as presented in the main text. The main difference between this set of SeqFold2D models and those in Fig H in S1 Text (with Stral-NR100 split into the TR, VL, and TS sets) is the slightly larger TR set used here, while the training hyperparameters are kept the same for models with the same size. Somewhat surprisingly, this set of models show slightly lower F1 scores for the TR set compared with those shown in Fig H in S1 Text. We do not have good explanations for the drops and did not further investigate the causes as the F1 scores for the VL set are very close. The SeqFold2D-1.4M* model was trained following the similar choices made by E2Efold and Ufold, specially with the cross-entropy loss function only and a weight of 300 for positive labels. As the shown TR and VL F1 scores were saved during training without post-processing, the scores from the SeqFold2D-1.4M* model are significantly lower than that after post-processing. For example, the averaged F1 score for the TR set increases from 0.898 to 0.981 with post processing for SeqFold2D-1.4M*. (B) The dependence of model performance (SeqFold2D-420K) on the size of the seen dataset (TR and VL) denoted in the x axis labels. Random sampling of the parent dataset (Stral-NR100) is used here, in contrast with the similarity-based de-redundancy method with CD-HIT-EST. A gradual decrease of model performance is observed as the data size decreases. **Fig J. The F1 scores of the TR+VL set (Stralign NR100, left in tan) and the TS set (ArchiveII NR100, right in blue) for the Ufold model with (A) and without (B) post-processing.** The leftmost pair of violins show the F1 scores for the entire sets and the following violin pairs show each constituent RNA family. Averaged scores are shown at the very top and also as dashed lines (white) within the violins. The values in the parentheses above are the sequence counts in actual numbers (for the whole set or families with <1% shares) or in percentages (for families with >1% shares). Note that 23S rRNA only exists in ArchiveII NR100. **Fig K. The F1 scores of the TR (left, tan) and VL (right, violet) sets for SeqFold2D-1.4M developed with the Stralign NR100 (Stral-NR100) dataset randomly split into TR and VL sets.** It is the same SeqFold2D-1.4M model shown in Fig I in S1 Text. No significant TR-VL variances (i.e., overfitting) are observed for the whole set or individual RNA families. **Fig L. The F1 scores of the TR (top, tan), VL (middle, violet), and TS (bottom, blue) sets for the SeqFold2D models developed with Stral-NR80 as TR and VL and Archi-Stral-NR80 as TS.** All SeqFold2D models exhibit significant TR-VL variances (i.e., overfitting), while still attaining decent performances over the TS set. The two smallest models (400K and 420K) have design variables of (N = 3, C = 48) and (N = 7, C = 32), respectively. It is worth noting that increasing the number of parameters from 960K to 1.4M did not increase the performances on the TR and VL sets but resulted in slightly better performances on the TS set. **Fig M. The F1 scores of the TR (tan, left), VL (violet, middle), and TS (blue, right) sets on the entire ([TOTAL]) and individual RNA families for the same SeqFold2D-1.4M model as shown in Fig L in S1 Text.** The order along the *x* axis follows the F1 scores of the TR set. Note that the TS set does not have tmRNA or TERC sequences after removing sequences with above 80% similarity with the Stral-NR80 dataset. The main observation is that large TR-VL and TR-TS variances are observed for all RNA families and that the TR-TS variance is usually much larger than the corresponding TR-VL variance except for the SRP family. **Fig N. Visualization of the TR (Stral-NR80) vs. TS (Archi-Stral-NR80) gaps for SeqFold2D and selected DL, ML, and physics-based models.** The models are ordered by the TS F1 score. We retrained five models (Ufold, MXfold2, ContextFold, Tornado, and ContraFold) but failed to retrain SPOT-RNA. It should be noted that we were unable to reproduce the same levels of performance for the DL models (Ufold and MXfold2) as their published parameters when using the same datasets (Stral-NR100 or bpRNA). As such, the performances of the DL models shown here do not represent their true capabilities and should be considered as for reference only. Note that the physics-based LinearFold-C is used in this study, while the LinearFold-C is based on the ContraFold parameters and thus expected to perform similarly to Contrafold if retrained. **Fig O. Illustration of the effect of dropout rates on the performance and generalization of the SeqFold2D-960K model with the TR and VL sets derived from Stral-NR80 and Archi-Stral-NR80 as TS.** Shown for each dropout rate are the F1 scores of the TR (left, tan), VL (middle, violet), and TS (right, blue) sets. In terms of performance, the TR F1 score steadily decreases with increasing dropout rate and, interestingly, the VL and TS F1 scores peak around the same rate between 0.2 and 0.3 (as adopted by the final SeqFold2D models). As for generalization, zero dropout leads to largest TR-VL and TR-TS variances and the dropout rates above 0.5 reduce both to zero. While regularization can indeed tune both performance and generalization, the two metrics are conflicting with each other and one has to balance them in accord to the needs. Note that the optimal dropout is expected to depend on the exact sequence distributions, as well as other model parameters. **Fig P. The F1 scores of the training (top, tan), validation (middle, violet), and test (bottom, blue) sets for selected DL, ML, and physics-based models.** Here the training, validation, and test sets are the bpRNA TR0, VL0, and TS0 datasets compiled by the SPOT-RNA team, respectively. The three datasets are expected to have independent, identical distributions, which are reflected by their comparable prediction performances by traditional algorithms. As discussed in the main text, the SeqFold2D models were trained to optimize the performance on the validation set, regardless of the magnitude of the train-validation variances. Ufold does not provide the saved model parameters trained with the bpRNA dataset, and thus only the value for the bpRNA TS0 set is available from the Ufold article [10]. Notably, rather decent F1 scores can be achieved on the bpRNA TR0 set, rapidly improving from 0.711 to 0.840 to 0.903 for the SeqFold2D-960K, 1.4M, and 3.5M models, respectively, but this results in rather small gains on the TS0 set (0.625, 0.642, and 0.665, correspondingly). The generalization gap can be reduced by model regularization which again fails to achieve both performance and generalization as shown in Fig Q in S1 Text for the case of dropout rate. We further note that the SeqFold2D models show even worse generalizability for the bpRNA-NEW dataset and we plan to use data augmentations techniques demonstrated by Ufold to improve generalizability in future work. **Fig Q. The scan of dropout rates for the SeqFold2D-960K model with the bpRNA TR0, VL0, and TS0 datasets.** The observations are in qualitative agreement with the dropout scan with the Stral-NR80 and Archi-Stral-NR80 datasets shown in Figs O in S1 Text. The training set (TR0) F1 score decreases monotonically with the dropout rate; the validation and test scores peak around relatively low dropout rates ~0.10. The TR0-TS0 gap does decrease with the increase of dropout but high dropout rates lead to very low absolute performances. The rightmost set (0.23*) shows the final SeqFold2D-960K model after additional optimizations of performance and generalizability tradeoffs. **Fig R. Illustrations of the TR (left, tan) vs. TS (right, blue) performances at the cross-family level with the Strive-NR80 dataset.** This is an extended plot of Fig 4 in the main text by showing all nine cross-family studies. Detailed captioning follows that of Fig 4 as well. **Fig S. The cross-family study with tRNA as the TR and VL sets and all other families as the TS set.** The DL model is SeqFold2D-400K and the parent dataset is Strive-NR80. Note that model training was stopped when the TR-VL variance became significant for this study. While the model displays excellent performances over the seen sequences (the first violin), the performances over other family types fail completely. **Fig T. Illustration of the PGscores of all cross-sequence and cross-cluster studies presented in this work.** Each row shows one study as labelled to the right. The models are sorted by the PGscore in descending order from left to right. For each model, the pair of violins show the F1 score distributions of TR (left, tan) and TS (right, blue) with its PGscore shown above. The names of the studies follow that in Fig 5B. Specifically, (A) XSeq-I: the cross-sequence study with Stral-NR100 only, (B) XSeq-II: cross-sequence with Stral-NR100 and Archi-NR100, (C) XCls-I: cross-cluster with Strive-NR80 only, (D) XCls-II: cross-cluster with Stral-NR80 and Archi-Stral-NR80, (E) XCls-III: cross-cluster with bpRNA. The cross-family studies are shown in Fig U in S1 Text. **Fig U. Illustration of the PGscores of all cross-family studies presented in this work. Each row shows one study as labelled to the right.** The first row is the base-line cross-cluster study with Strive-NR80 (the same as (C) XCls-I in Fig T in S1 Text). For each model, the pair of violins show the F1 score distributions of TR (left, tan) and TS (right, blue) with its PGscore shown above. The highest PGscore among the learning-based models (the first six models) is shown in bold. Fig V. The correlations between the estimated variances and the actual values of the F1 scores on the training (A, bpRNA TS0), validation (B, bpRNA VL0), test (C, bpRNA TS0), and another independent test (D, bpRNA-New) datasets for the SeqFold2D-960K model. Fig W. Illustrations of the correlation between the F1-unseen and F1-seen scores of the Ufold-8.6M* model shown in Fig N in S1 Text. Captioning follows that of Fig 6 in the main text. Fig X. Illustrations of the correlation between the F1-unseen and F1-seen scores of the MXfold2-800K model shown in Fig N in S1 Text. Captioning follows that of Fig 6 in the main text. Note that we were only able to re-train MXfold2 on Stral-NR80 to attain the F1 score of 0.797 (Fig N in S1 Text), far below the F1~0.922 for Stral-NR100 attained by the published model (Fig B in S1 Text). Thus the shown MXfold2 model appears under-retrained, leading to poor performances and excellent generalization. **Fig Y. Illustrations of the correlation between the F1-unseen and F1-seen scores of the ContextFold-74K model shown in Fig N in S1 Text.** Captioning follows that of Fig 6 in the main text. **Fig Z. Illustrations of the correlation between the F1-unseen and F1-seen scores of the Tornado-91K model shown in Fig N in S1 Text.** Captioning follows that of Fig 6 in the main text. **Fig AA. Illustrations of the correlation between the F1-unseen and F1-seen scores of the CONTRAfold-700 model shown in Fig N in S1 Text.** Captioning follows that of Fig 6 in the main text. **Fig BB. Illustrations of the correlation between the F1-unseen and F1-seen scores of the LinearFold model shown in Fig N in S1 Text.** Captioning follows that of Fig 6 in the main text. **Fig CC. Illustrations of the correlation between the F1-unseen and F1-seen scores of the RNAstructure model shown in Fig N in S1 Text.** Captioning follows that of Fig 6 in the main text. **Fig DD. Illustrations of the correlation between the F1-unseen and F1-seen scores of the RNAfold model shown in Fig N in S1 Text.** Captioning follows that of Fig 6 in the main text. **Fig EE. Comparisons of the pairwise PSI (FoldAlign) and PSSI (RNAdistance) scores and the dependences of SeqFold2D-960K performances on the similarities in RNA sequence and structure.** Both PSI and PSSI scores are from the pairwise alignments between the unseen set (Archi-Stral-NR80, 433 RNAs) and the seen set (Stral-NR80, 3122 RNAs). (A) Scatter plot of the PSI vs. PSSI score for each unseen-seen pair (1,351,826 total, down-sampled by a factor of 10). (B) Scatter plot of the maximum PSI vs. maximum PSSI score for each unseen RNA molecule. Note that the maximum PSI and PSSI scores may be obtained from a different seen sequence/structure. The unseen sequences are divided into two groups (blue and red) of comparable sizes by the PSI/PSSI score ratio. One group (blue circles) has PSI/PSSI ratios > 1.08, representing the low structure similarity population, while the other (red squares) has ratios < 1.08, representing the high structure similarity population. (C) The F1 score of the unseen sequence shown against its maximum PSI score, grouped by low (blue circles) and high (red squares) structure similarities as in (B). The blue dashed line and the red solid line show the average F1 scores as a function of the maximum PSI score for the low and high structure similarity groups, respectively. (D) Violin plot of the F1 score distribution of the low (blue, left) and high (red, right) structure similarity groups. The average F1 score for each group is show at the top with the number of sequences shown beneath. **Fig FF. Comparisons of the pairwise PSI (FoldAlign) and PSSI (RNAdistance) scores and the dependences of MXfold-800K performances on the similarities in RNA sequence and structure. Description of each panel follows that of Fig EE in S1 Text. Note that the model is retrained with the Stral-NR80 dataset by us. Fig GG. Comparisons of the pairwise PSI (FoldAlign) and PSSI (RNAdistance) scores and the dependences of Ufold-8.6M performances on the similarities in RNA sequence and structure. Description of each panel follows that of Fig EE in S1 Text. Note that the model is retrained with the Stral-NR80 dataset by us. Fig HH. Comparisons of the pairwise PSI (FoldAlign) and PSSI (RNAdistance) scores and the dependences of ContextFold-74K performances on the similarities in RNA sequence and structure. Description of each panel follows that of Fig EE in S1 Text. Note that the model is retrained with the Stral-NR80 dataset by us. Fig II. Comparisons of the pairwise PSI (FoldAlign) and PSSI (RNAdistance) scores and the dependences of CONTRAfold-700 performances on the similarities in RNA sequence and structure. Description of each panel follows that of Fig EE in S1 Text. Note that the model is retrained with the Stral-NR80 dataset by us. Fig JJ. Comparisons of the pairwise PSI (FoldAlign) and PSSI (RNAdistance) scores and the dependences of RNAfold performances on the similarities in RNA sequence and structure. Description of each panel follows that of Fig EE in S1 Text. Fig KK. Comparisons of the pairwise PSI (FoldAlign) and PSSI (RNAdistance) scores and the dependences of RNAstructure performances on the similarities in RNA sequence and structure. Description of each panel follows that of Fig EE in S1 Text. Fig LL. Comparisons of the pairwise PSI (FoldAlign) and PSSI (RNAdistance) scores and the dependences of LinearFold performances on the similarities in RNA sequence and structure. Description of each panel follows that of Fig EE in S1 Text. Fig MM. Comparisons of the PSI (by FoldAlign, left, tan) vs. PSSI (by RNAforester, right, blue) score distributions.** Each distribution is generated from pairwise alignments between two datasets, the unseen and seen datasets. Each row/panel shows the results from one unseen set given by the label to the right (A-H). The unseen set for (A) [ALL] is the entire Archi-Stral-NR80 dataset (433 sequences) and the unseen sets for the other panels (B-H) are the labelled RNA families in the Archi-Stral-NR80 dataset. The seen dataset is the entire Stralign NR80 dataset ([TOTAL]) or the specific RNA family in Stralign NR80 given in the x axis label. The average PSI and PSSI values are shown above the violins and the largest PSSI value for each panel is shown in bold. **Fig NN. Comparisons of the PSI (by FoldAlign, left, tan) vs. PSSI (by RNAdistance, right, blue) score distributions.** Captioning follows that of Fig MM in S1 Text.
(PDF)

## References

[1] Higgs PG. RNA secondary structure: physical and computational aspects. Q Rev Biophys. 2000;33(3):199–253. doi: 10.1017/s0033583500003620 11191843

[2] Fallmann J, Will S, Engelhardt J, Gruning B, Backofen R, Stadler PF. Recent advances in RNA folding. J Biotechnol. 2017;261:97–104. doi: 10.1016/j.jbiotec.2017.07.007 28690134

[3] Chen SJ. RNA folding: conformational statistics, folding kinetics, and ion electrostatics. Annu Rev Biophys. 2008;37:197–214. doi: 10.1146/annurev.biophys.37.032807.125957 18573079 PMC2473866

[4] Cech TR, Steitz JA. The noncoding RNA revolution-trashing old rules to forge new ones. Cell. 2014;157(1):77–94. doi: 10.1016/j.cell.2014.03.008 24679528

[5] Diederichs S. The four dimensions of noncoding RNA conservation. Trends Genet. 2014;30(4):121–3. doi: 10.1016/j.tig.2014.01.004 24613441

[6] Bevilacqua PC, Ritchey LE, Su Z, Assmann SM. Genome-Wide Analysis of RNA Secondary Structure. In: Bonini NM, editor. Annual Review of Genetics, Vol 50. Annual Review of Genetics. 502016. p. 235–66. doi: 10.1146/annurev-genet-120215-035034 27648642

[7] Rouskin S, Zubradt M, Washietl S, Kellis M, Weissman JS. Genome-wide probing of RNA structure reveals active unfolding of mRNA structures in vivo. Nature. 2014;505(7485):701–5. doi: 10.1038/nature12894 24336214 PMC3966492

[8] Mauger DM, Cabral BJ, Presnyak V, Su SV, Reid DW, Goodman B, et al. mRNA structure regulates protein expression through changes in functional half-life. Proc Natl Acad Sci U S A. 2019;116(48):24075–83. doi: 10.1073/pnas.1908052116 31712433 PMC6883848

[9] Ermolenko DN, Mathews DH. Making ends meet: new functions of mRNA secondary structure. Wiley Interdisciplinary Reviews: RNA. 2021;12(2):e1611. 32597020 10.1002/wrna.1611 PMC8107001

[10] Seetin MG, Mathews DH. RNA structure prediction: an overview of methods. Methods Mol Biol. 2012;905:99–122. doi: 10.1007/978-1-61779-949-5_8 22736001

[11] Deigan KE, Li TW, Mathews DH, Weeks KM. Accurate SHAPE-directed RNA structure determination. Proc Natl Acad Sci U S A. 2009;106(1):97–102. doi: 10.1073/pnas.0806929106 19109441 PMC2629221

[12] Eddy SR. Computational Analysis of Conserved RNA Secondary Structure in Transcriptomes and Genomes. Annual Review of Biophysics. 2014;43(1):433–56. doi: 10.1146/annurev-biophys-051013-022950 24895857 PMC5541781

[13] Zhao Q, Zhao Z, Fan X, Yuan Z, Mao Q, Yao Y. Review of machine learning methods for RNA secondary structure prediction. PLoS Comput Biol. 2021;17(8):e1009291. doi: 10.1371/journal.pcbi.1009291 34437528 PMC8389396

[14] Leontis NB, Westhof E. Geometric nomenclature and classification of RNA base pairs. RNA. 2001;7(4):499–512. doi: 10.1017/s1355838201002515 11345429 PMC1370104

[15] Gutell RR. Ten lessons with Carl Woese about RNA and comparative analysis. RNA Biol. 2014;11(3):254–72. doi: 10.4161/rna.28718 24713659

[16] Andronescu M, Condon A, Turner DH, Mathews DH. The Determination of RNA Folding Nearest Neighbor Parameters. In: Gorodkin J, Ruzzo WL, editors. RNA Sequence, Structure, and Function: Computational and Bioinformatic Methods. Totowa, NJ: Humana Press; 2014. p. 45–70. 10.1007/978-1-62703-709-9_3 24639154

[17] Rivas E. The four ingredients of single-sequence RNA secondary structure prediction. A unifying perspective. RNA Biol. 2013;10(7):1185–96. doi: 10.4161/rna.24971 23695796 PMC3849167

[18] Zuker M, Stiegler P. Optimal computer folding of large RNA sequences using thermodynamics and auxiliary information. Nucleic Acids Res. 1981;9(1):133–48. doi: 10.1093/nar/9.1.133 6163133 PMC326673

[19] Danaee P, Rouches M, Wiley M, Deng D, Huang L, Hendrix D. bpRNA: large-scale automated annotation and analysis of RNA secondary structure. Nucleic Acids Res. 2018;46(11):5381–94. doi: 10.1093/nar/gky285 29746666 PMC6009582

[20] Gutell RR, Lee JC, Cannone JJ. The accuracy of ribosomal RNA comparative structure models. Curr Opin Struct Biol. 2002;12(3):301–10. doi: 10.1016/s0959-440x(02)00339-1 12127448

[21] Mao K, Wang J, Xiao Y. Prediction of RNA secondary structure with pseudoknots using coupled deep neural networks. Biophysics Reports. 2020;6(4):146–54.

[22] Wang Y, Liu Y, Wang S, Liu Z, Gao Y, Zhang H, et al. ATTfold: RNA Secondary Structure Prediction With Pseudoknots Based on Attention Mechanism. Frontiers in Genetics. 2020;11:612086. doi: 10.3389/fgene.2020.612086 33384721 PMC7770172

[23] Wang L, Liu Y, Zhong X, Liu H, Lu C, Li C, et al. DMfold: A Novel Method to Predict RNA Secondary Structure With Pseudoknots Based on Deep Learning and Improved Base Pair Maximization Principle. Front Genet. 2019;10(143):143. doi: 10.3389/fgene.2019.00143 30886627 PMC6409321

[24] Chen X, Li Y, Umarov R, Gao X, Song L. RNA Secondary Structure Prediction By Learning Unrolled Algorithms 2020 February 01, 2020:[arXiv:2002.05810 p.]. Available from: https://ui.adsabs.harvard.edu/abs/2020arXiv200205810C.

[25] Sato K, Akiyama M, Sakakibara Y. RNA secondary structure prediction using deep learning with thermodynamic integration. Nat Commun. 2021;12(1):941. doi: 10.1038/s41467-021-21194-4 33574226 PMC7878809

[26] Singh J, Hanson J, Paliwal K, Zhou Y. RNA secondary structure prediction using an ensemble of two-dimensional deep neural networks and transfer learning. Nat Commun. 2019;10(1):5407. doi: 10.1038/s41467-019-13395-9 31776342 PMC6881452

[27] Fu L, Cao Y, Wu J, Peng Q, Nie Q, Xie X. UFold: fast and accurate RNA secondary structure prediction with deep learning. Nucleic Acids Res. 2022;50(3):e14. doi: 10.1093/nar/gkab1074 34792173 PMC8860580

[28] Mao K, Wang J, Xiao Y. Length-Dependent Deep Learning Model for RNA Secondary Structure Prediction. Molecules. 2022;27(3):1030. doi: 10.3390/molecules27031030 35164295 PMC8838716

[29] Zhang H, Zhang C, Li Z, Li C, Wei X, Zhang B, et al. A New Method of RNA Secondary Structure Prediction Based on Convolutional Neural Network and Dynamic Programming. Front Genet. 2019;10(467):467. doi: 10.3389/fgene.2019.00467 31191603 PMC6540740

[30] Wu H, Tang Y, Lu W, Chen C, Huang H, Fu Q, editors. RNA Secondary Structure Prediction Based on Long Short-Term Memory Model 2018; Cham: Springer International Publishing.

[31] Lu W, Tang Y, Wu H, Huang H, Fu Q, Qiu J, et al. Predicting RNA secondary structure via adaptive deep recurrent neural networks with energy-based filter. BMC Bioinformatics. 2019;20(Suppl 25):684. doi: 10.1186/s12859-019-3258-7 31874602 PMC6929275

[32] Szikszai M, Wise M, Datta A, Ward M, Mathews DH. Deep learning models for RNA secondary structure prediction (probably) do not generalize across families. Bioinformatics (Oxford, England). 2022;38(16):3892–9. doi: 10.1093/bioinformatics/btac415 35748706 PMC9364374

[33] Flamm C, Wielach J, Wolfinger MT, Badelt S, Lorenz R, Hofacker IL. Caveats to Deep Learning Approaches to RNA Secondary Structure Prediction. Front Bioinform. 2022;2:835422. doi: 10.3389/fbinf.2022.835422 36304289 PMC9580944

[34] Willmott D, Murrugarra D, Ye Q. Improving RNA secondary structure prediction via state inference with deep recurrent neural networks. Computational and Mathematical Biophysics. 2020;8(1):36–50.

[35] Reuter JS, Mathews DH. RNAstructure: software for RNA secondary structure prediction and analysis. BMC Bioinformatics. 2010;11(1):129. doi: 10.1186/1471-2105-11-129 20230624 PMC2984261

[36] Lorenz R, Bernhart SH, Honer Zu Siederdissen C, Tafer H, Flamm C, Stadler PF, et al. ViennaRNA Package 2.0. Algorithms Mol Biol. 2011;6(1):26. doi: 10.1186/1748-7188-6-26 22115189 PMC3319429

[37] Fu L, Niu B, Zhu Z, Wu S, Li W. CD-HIT: accelerated for clustering the next-generation sequencing data. Bioinformatics. 2012;28(23):3150–2. doi: 10.1093/bioinformatics/bts565 23060610 PMC3516142

[38] Tan Z, Fu Y, Sharma G, Mathews DH. TurboFold II: RNA structural alignment and secondary structure prediction informed by multiple homologs. Nucleic Acids Res. 2017;45(20):11570–81. doi: 10.1093/nar/gkx815 29036420 PMC5714223

[39] Sloma MF, Mathews DH. Exact calculation of loop formation probability identifies folding motifs in RNA secondary structures. RNA. 2016;22(12):1808–18. doi: 10.1261/rna.053694.115 27852924 PMC5113201

[40] Zakov S, Goldberg Y, Elhadad M, Ziv-Ukelson M. Rich parameterization improves RNA structure prediction. J Comput Biol. 2011;18(11):1525–42. doi: 10.1089/cmb.2011.0184 22035327

[41] Do CB, Woods DA, Batzoglou S. CONTRAfold: RNA secondary structure prediction without physics-based models. Bioinformatics. 2006;22(14):e90–8. doi: 10.1093/bioinformatics/btl246 16873527

[42] Rivas E, Lang R, Eddy SR. A range of complex probabilistic models for RNA secondary structure prediction that includes the nearest-neighbor model and more. RNA. 2012;18(2):193–212. doi: 10.1261/rna.030049.111 22194308 PMC3264907

[43] Jumper J, Evans R, Pritzel A, Green T, Figurnov M, Ronneberger O, et al. Highly accurate protein structure prediction with AlphaFold. Nature. 2021;596(7873):583–9. doi: 10.1038/s41586-021-03819-2 34265844 PMC8371605

[44] Sundfeld D, Havgaard JH, de Melo ACMA, Gorodkin J. Foldalign 2.5: multithreaded implementation for pairwise structural RNA alignment. Bioinformatics. 2015;32(8):1238–40. doi: 10.1093/bioinformatics/btv748 26704597 PMC4824132

[45] Winkler J, Urgese G, Ficarra E, Reinert K. LaRA 2: parallel and vectorized program for sequence–structure alignment of RNA sequences. BMC Bioinformatics. 2022;23(1):18. doi: 10.1186/s12859-021-04532-7 34991448 PMC8734264

[46] Will S, Joshi T, Hofacker IL, Stadler PF, Backofen R. LocARNA-P: accurate boundary prediction and improved detection of structural RNAs. RNA. 2012;18(5):900–14. doi: 10.1261/rna.029041.111 22450757 PMC3334699

[47] Mathews DH, Turner DH. Dynalign: an algorithm for finding the secondary structure common to two RNA sequences11Edited by I. Tinoco. Journal of Molecular Biology. 2002;317(2):191–203. 11902836 10.1006/jmbi.2001.5351

[48] Bayegan AH, Clote P. RNAmountAlign: Efficient software for local, global, semiglobal pairwise and multiple RNA sequence/structure alignment. Plos One. 2020;15(1):e0227177. doi: 10.1371/journal.pone.0227177 31978147 PMC6980424

[49] Camacho C, Coulouris G, Avagyan V, Ma N, Papadopoulos J, Bealer K, et al. BLAST+: architecture and applications. BMC Bioinformatics. 2009;10(1):421. doi: 10.1186/1471-2105-10-421 20003500 PMC2803857

[50] Nawrocki EP, Kolbe DL, Eddy SR. Infernal 1.0: inference of RNA alignments. Bioinformatics. 2009;25(10):1335–7. doi: 10.1093/bioinformatics/btp157 19307242 PMC2732312

[51] Blin G, Denise A, Dulucq S, Herrbach C, Touzet H. Alignments of RNA Structures. IEEE/ACM Transactions on Computational Biology and Bioinformatics. 2010;7(2):309–22. doi: 10.1109/TCBB.2008.28 20431150

[52] Murdoch WJ, Singh C, Kumbier K, Abbasi-Asl R, Yu B. Definitions, methods, and applications in interpretable machine learning. Proceedings of the National Academy of Sciences. 2019;116(44):22071–80. doi: 10.1073/pnas.1900654116 31619572 PMC6825274

[53] Felden B. RNA structure: experimental analysis. Curr Opin Microbiol. 2007;10(3):286–91. doi: 10.1016/j.mib.2007.05.001 17532253

[54] Kertesz M, Wan Y, Mazor E, Rinn JL, Nutter RC, Chang HY, et al. Genome-wide measurement of RNA secondary structure in yeast. Nature. 2010;467(7311):103–7. doi: 10.1038/nature09322 20811459 PMC3847670

[55] Lucks JB, Mortimer SA, Trapnell C, Luo S, Aviran S, Schroth GP, et al. Multiplexed RNA structure characterization with selective 2′-hydroxyl acylation analyzed by primer extension sequencing (SHAPE-Seq). Proceedings of the National Academy of Sciences. 2011;108(27):11063–8. doi: 10.1073/pnas.1106501108 21642531 PMC3131332

[56] Zhang T, Singh J, Litfin T, Zhan J, Paliwal K, Zhou Y. RNAcmap: a fully automatic pipeline for predicting contact maps of RNAs by evolutionary coupling analysis. Bioinformatics. 2021;37(20):3494–500. doi: 10.1093/bioinformatics/btab391 34021744

[57] Zhang C, Zhang Y, Marie Pyle A. rMSA: a sequence search and alignment algorithm to improve RNA structure modeling. Journal of Molecular Biology. 2022:167904. 10.1016/j.jmb.2022.167904 37356900

[58] Devlin J, Chang M-W, Lee K, Toutanova K. Bert: Pre-training of deep bidirectional transformers for language understanding. arXiv preprint arXiv:181004805. 2018.

[59] The RC, Petrov AI, Kay SJE, Kalvari I, Howe KL, Gray KA, et al. RNAcentral: a comprehensive database of non-coding RNA sequences. Nucleic Acids Res. 2017;45(D1):D128–D34. doi: 10.1093/nar/gkw1008 27794554 PMC5210518

[60] Karniadakis GE, Kevrekidis IG, Lu L, Perdikaris P, Wang S, Yang L. Physics-informed machine learning. Nature Reviews Physics. 2021;3(6):422–40.

[61] Zhao Y, Wang J, Zeng C, Xiao Y. Evaluation of RNA secondary structure prediction for both base-pairing and topology. Biophysics Reports. 2018;4(3):123–32.

[62] Pearce R, Omenn GS, Zhang Y. De Novo RNA Tertiary Structure Prediction at Atomic Resolution Using Geometric Potentials from Deep Learning. bioRxiv. 2022:2022.05.15.491755.

[63] Mathews DH. How to benchmark RNA secondary structure prediction accuracy. Methods. 2019;162–163:60–7. doi: 10.1016/j.ymeth.2019.04.003 30951834 PMC7202366

[64] Zuker M. Mfold web server for nucleic acid folding and hybridization prediction. Nucleic Acids Res. 2003;31(13):3406–15. doi: 10.1093/nar/gkg595 12824337 PMC169194

[65] Huang L, Zhang H, Deng D, Zhao K, Liu K, Hendrix DA, et al. LinearFold: linear-time approximate RNA folding by 5’-to-3’ dynamic programming and beam search. Bioinformatics. 2019;35(14):i295–i304. doi: 10.1093/bioinformatics/btz375 31510672 PMC6681470

[66] Andronescu M, Condon A, Hoos HH, Mathews DH, Murphy KP. Efficient parameter estimation for RNA secondary structure prediction. Bioinformatics. 2007;23(13):i19–28. doi: 10.1093/bioinformatics/btm223 17646296

[67] Sato K, Hamada M, Asai K, Mituyama T. CENTROIDFOLD: a web server for RNA secondary structure prediction. Nucleic acids research. 2009;37(Web Server issue):W277–W80. doi: 10.1093/nar/gkp367 19435882 PMC2703931

