# Supplementary material for: Sequence similarity governs generalizability of de novo deep learning models for RNA secondary structure prediction
Source: PLoS Comput Biol. 2023 Apr 17;19(4):e1011047. doi: 10.1371/journal.pcbi.1011047 (PMC10138783; doi:10.1371/journal.pcbi.1011047)
Supplement: S1 Text — Fig A. The population distributions of RNA families in the Stralign dataset at different sequence redundancy levels. This is an unscaled version of Fig 1 in the main text and the TERC (telomerase RNA) population is too small and barely visible. The innermost ring shows the original Stralign dataset. The L600 ring is after removing lengths over 600; the NR100 rings shows the cross-sequence level; and the NR80 ring shows the cross-cluster level. Fig B. The length distribution of each RNA family in the Stralign NR100 (A) and Stralign-NR80 (B) datasets shown in the same order and color as in Fig A in S1 Text. The number of each family type and its percentage in the parent dataset are shown in the legend. Fig C. The population distributions of all RNA families of the RNA ArchiveII dataset at different sequence redundancy levels. Two versions for the same underlying datasets are shown, the unscaled version (A) and the scaled version (B) for visibility of the underrepresented families that are scaled up by the multiplier N shown in the label. With the same notations as used in Fig A in S1 Text, the innermost ring shows the relative populations of the RNA families in the original ArchiveII dataset. The L600 rings are after removing lengths over 600; the NR100 rings show the cross-sequence levels; and the NR80 rings show the cross-cluster levels. Note that group II intron, labelled as Intron II, all have lengths longer than 600 and are thus absent in the NR100 and NR80 datasets. Fig D. The length distributions of all RNA families in the ArchiveII NR100 (A) and NR80 (B) datasets. The order of the RNA families shown follows that of the Stralign datasets in Fig B in S1 Text to facilitate comparison, rather than by the order of population as in Fig C in S1 Text. Fig E. The distributions of RNA families in Strive NR100 (A) and Strive NR80 (B). Together shown are the corresponding Stralign and ArchiveII sets for comparison. The order of RNA families is sorted by the family abundan [file pcbi.1011047.s001.pdf]

Supplemental Information for “Sequence similarity governs generalizability of *de novo* deep learning models for RNA secondary structure prediction”

Xiangyun Qiu*
Department of Physics, George Washington University, Washington DC 20052

xqiu@gwu.edu

1. Datasets

The RNA Stralign dataset. The RNA Stralign dataset was curated in 2017 (1) for RNA sequence alignment and secondary structure predictions (hence highly redundant). Stralign was noted to have greater sequence diversity than previous datasets (e.g., BRAliBase (2)) and comprises several families longer than 320 nucleotides (up to 1851). The Stralign dataset has a total of 37,149 sequences distributed over eight RNA families as shown in Fig A in S1 Text. For the purpose of deep learning (DL) model development, duplicated sequences are removed and sequence lengths are limited to 600 nucleotides, resulting in 20,118 sequences referred to as the Stralign NR100 (i.e., non-redundancy at 100% identity level) dataset or Stral-NR100 in short. Overall, the sequence distributions in the Stral-NR100 dataset are highly uneven, presenting steep observational biases for training DL models. This is first reflected in the imbalanced representations of different RNA families, e.g., the top two families (5S rRNA and tRNA) account for nearly 80% and the bottom four families for less than 8%. Another is the family-specific, uneven sequence length distributions (shown in Fig B in S1 Text). It is noteworthy that the dataset contains many sub-domains taken out of full-length sequences.

As structure is more conserved than sequence, non-identical sequences can give highly resembling structures, adding another source of observational bias. One common mitigation is to remove similar sequences above certain 80% sequence identity level which has been shown to the inflection point of sequence-structure correlations (3). We consequently obtained such dataset, denoted as Stral-NR80, by reducing the Stral-NR100 dataset with the program CD-HIT to below 80% sequence identity (80% is also the lowest allowed by CD-HIT). As shown in Fig 1A in the main text and Fig A in S1 Text, this led to a dramatic reduction in size for Stral-NR80, with just 3,122 sequences or ~1/7th of the Stral-NR100 dataset. The most populous families (16S and 5S rRNA and tRNA) all have high levels of redundancy as large as 20 folds, whereas the less-represented families typically show less than 3-fold redundancy. Out of curiosity, we also verified that all cross-family sequence pairs are below 80% identity as expected. Therefore, in the context of the entire polynucleotide sequence space, these RNA families can be viewed as distinct clusters with inter-family dissimilarities (or distances) at least 80%, while each cluster itself also spans beyond 80% similarity levels. The exact intra- and inter-family dissimilarities are however unknown. As mentioned, each RNA family further has characteristic length distributions as shown in Fig B in S1 Text. These fundamental differences all present challenges for DL models to generalize over different families.

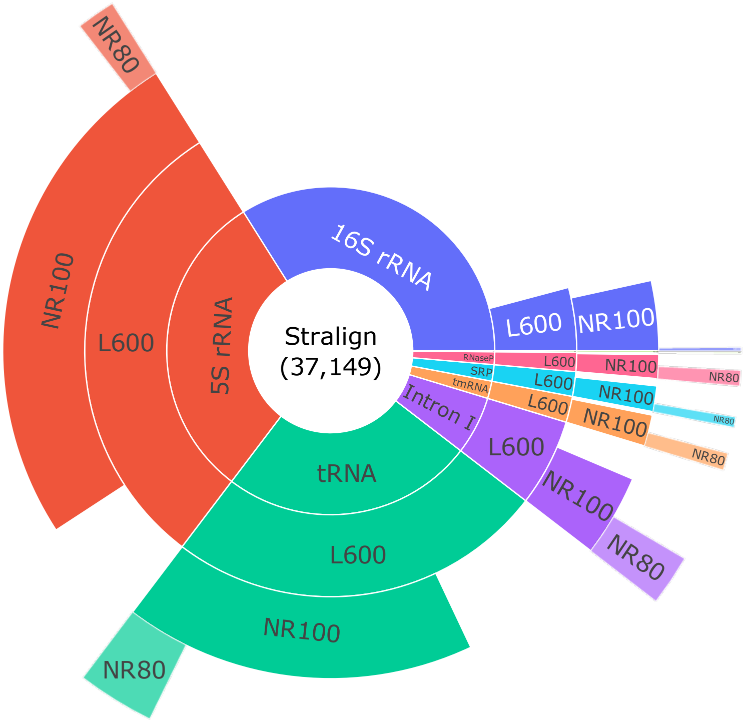

Fig A. The population distributions of RNA families in the Stralign dataset at different sequence redundancy levels. This is an unscaled version of Fig 1 in the main text and the TERC (telomerase RNA) population is too small and barely visible. The innermost ring shows the original Stralign dataset. The L600 ring is after removing lengths over 600; the NR100 rings shows the cross-sequence level; and the NR80 ring shows the cross-cluster level.

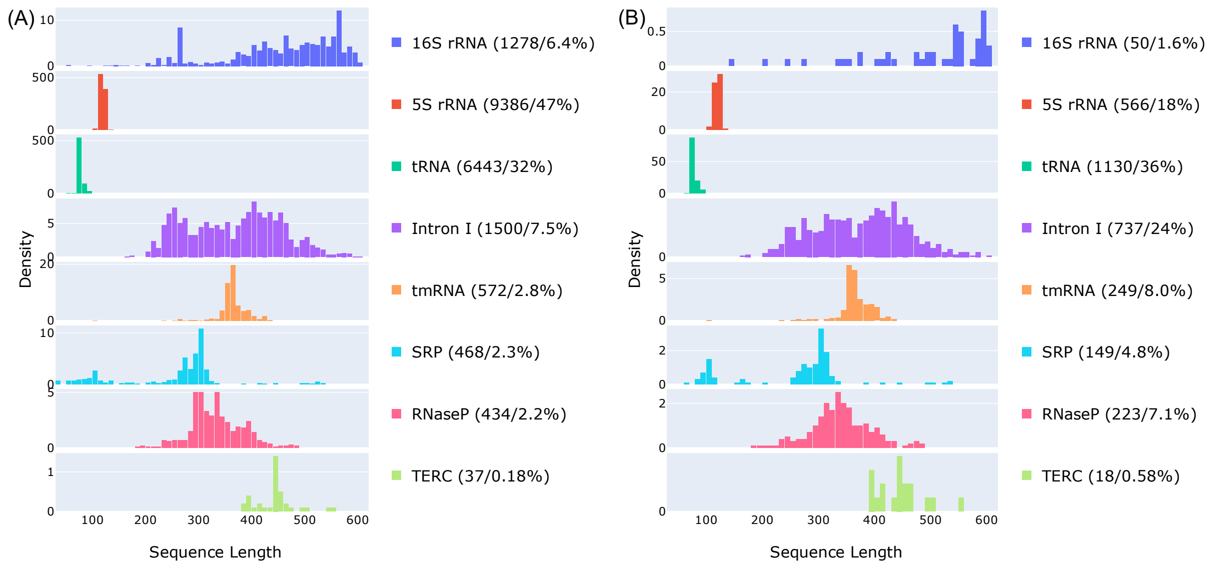

Fig B. The length distribution of each RNA family in the Stralign NR100 (A) and Stralign-NR80 (B) datasets shown in the same order and color as in Fig A in S1 Text. The number of each family type and its percentage in the parent dataset are shown in the legend.

The RNA ArchiveII dataset. The RNA ArchiveII dataset (4) is a collection of benchmarking RNA secondary structures determined by comparative sequence analysis. It is worth noting that the ArchiveII dataset has no non-canonical base pairs and no pseudoknots. Structures with unknown residues were also omitted and long sequences were divided into domains no longer than 700 nucleotides. In addition to the eight RNA families as in the Stralign dataset, ArchiveII includes two more families with longer sequences: 23S rRNA and group II intron, despite its much smaller size of 3,975 sequence in total (~11% of Stralign). As done for Stralign, we obtained ArchiveII NR100 dataset (Archi-NR100, 3395 sequences) by removing duplicates and sequences longer than 600 nucleotides. Note that all group II intron sequences are longer than 600 and thus absent in the Archi-NR100 set. The Archi-NR80 set (1221 sequences) was obtained by removing redundant sequences above 80% sequence identity. The population distributions and length distributions of all RNA families are shown in Figs B and C in S1 Text, respectively. For the cross-cluster study, we further removed the sequences in the Archi-NR80 dataset with above 80% sequence similarity level with the Stral-NR80 dataset, yielding the Archi-Stral-NR80 dataset (433 sequences).

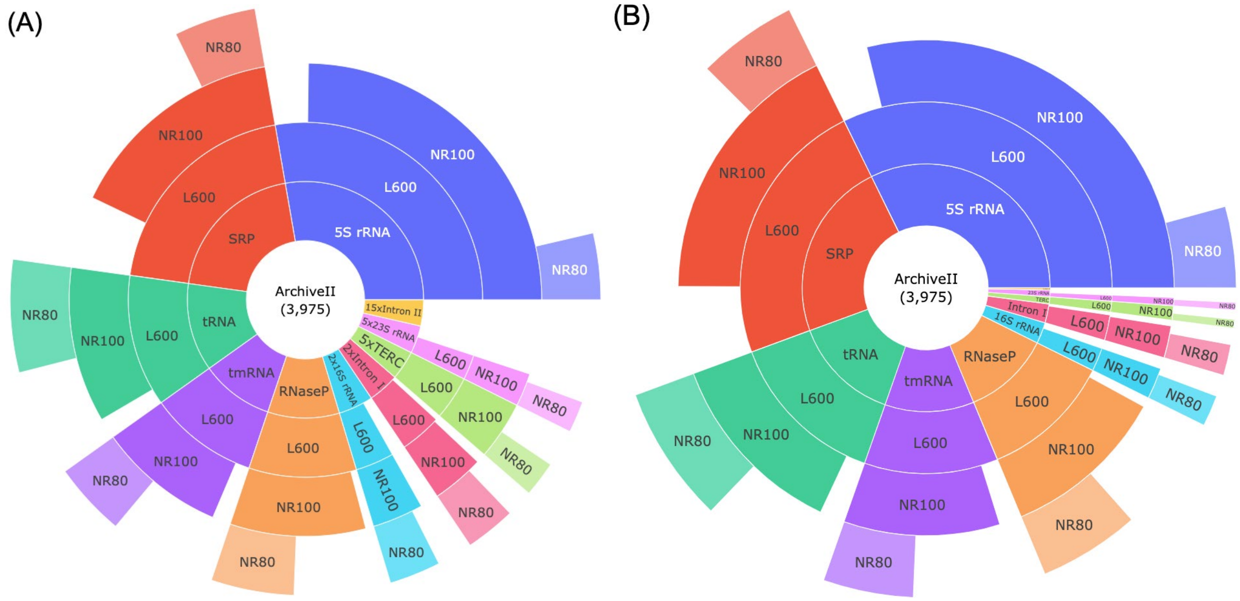

Fig C. The population distributions of all RNA families of the RNA ArchiveII dataset at different sequence redundancy levels. Two versions for the same underlying datasets are shown, the unscaled version (A) and the scaled version (B) for visibility of the underrepresented families that are scaled up by the multiplier N shown in the label. With the same notations as used in Fig A in S1 Text, the innermost ring shows the relative populations of the RNA families in the original ArchiveII dataset. The L600 rings are after removing lengths over 600; the NR100 rings show the cross-sequence levels; and the NR80 rings show the cross-cluster levels. Note that group II intron, labelled as Intron II, all have lengths longer than 600 and are thus absent in the NR100 and NR80 datasets.

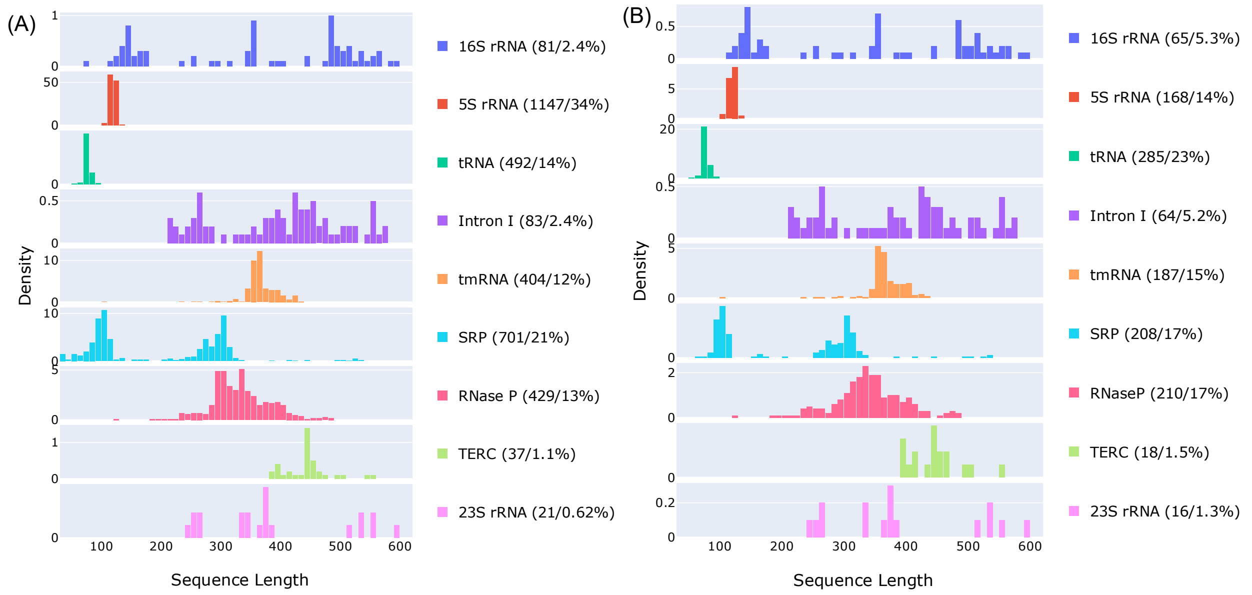

Fig D. The length distributions of all RNA families in the ArchiveII NR100 (A) and NR80 (B) datasets. The order of the RNA families shown follows that of the Stralign datasets in Fig B in S1 Text to facilitate comparison, rather than by the order of population as in Fig C in S1 Text.

The Strive dataset. The Strive dataset is the sum of the RNA Stralign and ArchiveII datasets with duplicated sequences removed. It is compiled mainly for the cross-family study. We followed the same procedure as done for the Stralign and ArchiveII datasets to obtain the Strive NR100 and Strive NR80 datasets, the family distributions of which are shown in Fig E in S1 Text. Specifically, the Strive NR100 contains non-duplicate sequences up to 600 nucleotides and the Strive NR80 further removes sequences above 80% similarity levels with CD-HIT.

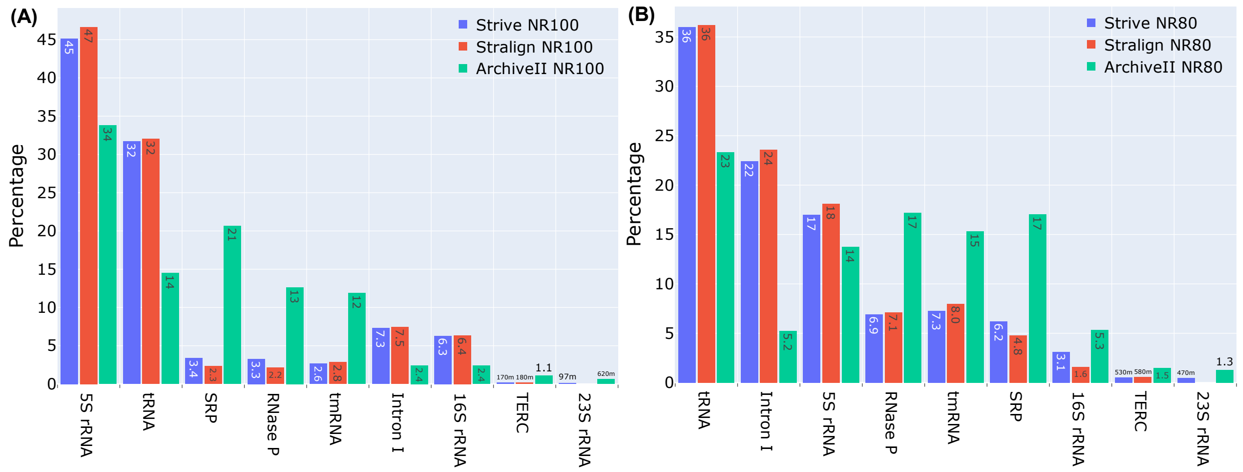

Fig E. The distributions of RNA families in Strive NR100 (A) and Strive NR80 (B). Together shown are the corresponding Stralign and ArchiveII sets for comparison. The order of RNA families is sorted by the family abundance summing over all three datasets.

The bpRNA dataset. The bpRNA-1m dataset (5) is a >100K RNA secondary structure collection (102,348) from seven databases: the comparative RNA web (CRW) site (55,600), tmRNA (728), SRP (959), SPR (623), RNP (466), RFAM (43,273), and PDB (669). Despite being the largest database for RNA secondary structures, its sequence distributions are highly uneven across its member databases. Like its member databases, the vast majority of the secondary structures in the bpRNA dataset are determined by comparative sequence analysis. The downloadable form however only provides the source (e.g., CRW or SRP) rather than the actual RNA family type. We thus chose Stralign and ArchiveII over bpRNA for detailed studies. For the development of DL models, one commonly used bpRNA-derived dataset is the TrainSet0 (TR0, 10,814 sequences), ValidSet0 (VL0, 1300), and TestSet0 (TS0, 1305) compiled by the SPOT-RNA team (6), with a total of 13,419 sequences. Specifically, sequence lengths are limited to be within 30 and 500 nucleotides, the sequence identity is trimmed to 80% with CD-HIT, and all sequences with high similarity levels to the PDB datasets are removed. Fig F in S1 Text shows the source and length distributions of the bpRNA TR0 and VL0 sets. The same dataset choices (i.e., TR0, VL0, TS0) were used by Ufold and MXfold2 with pre-trained parameters available, noting that MXfold2 further removed non-canonical base pairs and pseudoknots.

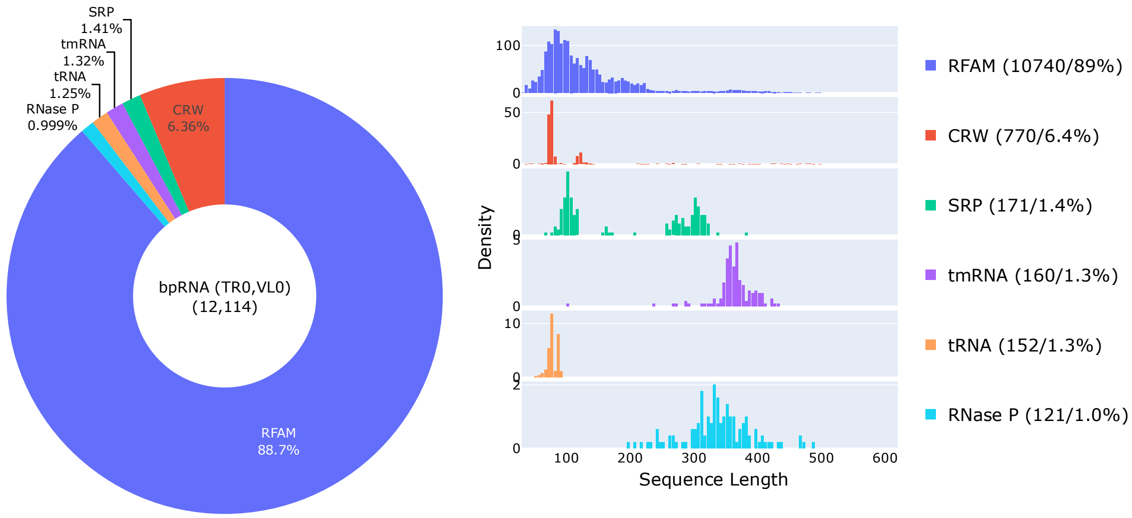

Fig F. The population distributions (left panel) and length distributions (right panel) of the sequences grouped by their sources in the bpRNA TR0 and VL0 datasets. The x-axis range is shown up to 600 nucleotides for easy comparisons with Figs B and D in S1 Text, while the actual sequences are all shorter than 500 nucleotides. The TS0 set has essentially the same source and length distributions and thus not shown separately.

The bpRNA-NEW dataset. The bpRNA-NEW dataset was compiled by the MXfold2 team (7) and it is based on the newly added ~1500 families to RFAM 14.2 since RFAM 12.2 used by the bpRNA-1m dataset. It has a total of 5401 sequences with lengths shorter than 500 nucleotides and sequence identities below 80% filtered by CD-HIT. However, both non-canonical base pairs and pseudoknots are removed from the database. The level of base pairing is relatively low with an average of ~45%. These secondary structures are more likely underestimates of the true levels of base pairing, particularly for these families without 3D RNA structures providing seeding secondary structures. We however did not present the results from this dataset, as it

typically leads to model performances between the cross-cluster and cross-family levels. For completeness, Fig G in S1 Text shows the length distribution of the bpRNA-NEW dataset.

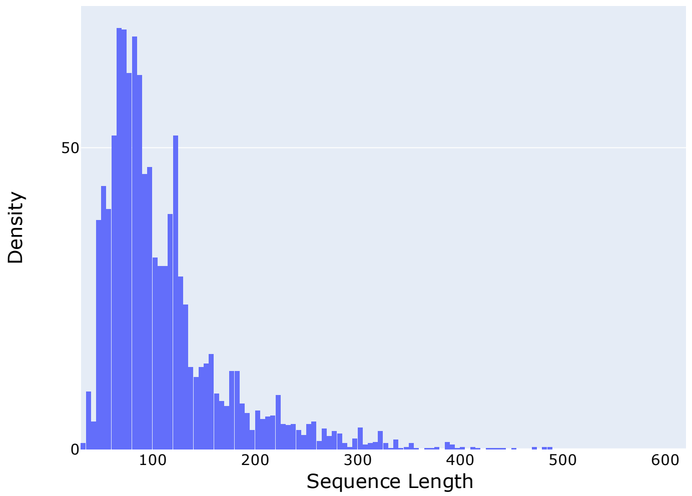

Fig G. The length distribution of the bpRNA-NEW dataset. It is qualitatively similar to the RFAM length distributions of the bpRNA TR0 and VL0 sets shown in Fig F in S1 Text.

2. Network architecture and training procedure

With 1D RNA sequences as inputs and 2D pairing probabilities as outputs, the overall network architecture has two main learning modules, as shown in Fig 1A in the main text. The first module consists of N1 stacked blocks of bidirectional Long-Short-Term-Memory (LSTM) or self-attention-based transformer encoders to learn richer 1D sequence representations, which are then transformed into 2D pair representations via outer-product. The second module consists of N2 stacked blocks of residual 2D convolutional layers to infer inter-nucleotide interactions. To reduce the design space of model hyperparameters, the numbers of blocks in both modules are kept the same, i.e., N1=N2=N. Layer normalization and dropout (0.2-0.42) layers are always applied after multiplications and additions with trainable weights and biases (except for the final output layer), where non-linear activations (LeakyReLU or Swish) are applied before. Note that the name Seqfold has been used for a method for reconstructing RNA structures from high-throughput sequencing data (8) and for another program (http://github.com/Lattice-Automation/seqfold), while we name our architecture SeqFold2D to emphasize the use of sequences as the only inputs and the output of 2D PPMs. Detailed description of each component is given below.

Sequence embedding. One-hot vectorization is used to digitize each nucleotide as a 1×4 vector, e.g., A as [1,0,0,0], C as [0,1,0,0], and an all-zero vector Z ([0,0,0,0]) for padding sequences to the same length. We further adopt a k-mer (k=3) representation for each base to include its neighbors. For example, ACGU is represented as four tokens of ZAC, ACG, CGU, and GUZ. Each RNA sequence of length L starts as a vector of shape $L\times12$, which then passes through one feed-forward layer to obtain its embedding vector of shape $L\times C$, where C is the channel size as a

model hyperparameter. We further keep the channel size C the same throughout the model. As a result, the model size in terms of the number of parameters is largely determined by two design variables, N and C.

Input block. Two feed-forward layers are used to further mix the different channels while keeping the tensor shape as L×C. It can be argued that these feed-forward layers are not absolutely necessary, though no ablation studies were conducted.

Module 1: 1D sequence encoding. Each block, repeated N times in the module comprises one LSTM or transformer encoder layer. In the case of LSTM blocks, normalization and dropout layers are added between blocks and no additional activation layers are used. In the case of transformer encoders, sinusoidal positional embedding is added before the first block. A constant head size of 16 is used for the multi-head self-attention, under the condition that the channel size C is a multiple of 16. Similar performances are observed with the use of either LSTM or transformer encoders for this module.

1D to 2D transformation. For the transformation from the L×C 1D representation to 2D L×L×C pair representation, we experimented with outer-concatenation and outer-product and found similar performances. Outer-product is the usual choice to maintain the same channel size. We have also experimented with the concatenation of 2D matrices of the all allowed canonical base pairs and the Turner-like energies of neighboring base pairs into the pair representation, similar to Ufold and DMfold. We found this leads to faster convergences in the beginning, particularly when self-attention-based transformer layers are used for the first module. However, this results in rather negligible improvements in the final model. Only the SeqFold2D-1.4M model developed with the Stral-NR100 dataset (shown in Fig 2B in the main text) uses such matrices in the inputs.

Module 2: residual 2D convolution. Each residual block comprises two 2D convolutional layers with kernel sizes of 5×5 and 3×3, respectively. The residual connection is done after activation and normalization layers to maintain a straight path for the so-called skip connection. It operates on the pair representation (L×L×C) and aims to facilitate the communication of each specific pairs with neighboring pairs.

Output block. The output block comprises of three fully connected layers that operate across the channel dimension only, i.e., no more communication between neighboring pairs. The dimension of the final layer is L×L×2 and Softmax is applied to get the matrix of unpaired probabilities and pairing probabilities, with the latter used for loss and metrics calculations.

Evaluation metrics. To compute the F1 score as defined in the main text, the continuous PPM is discretized as 0 or 1 with a threshold of 0.5 without grid search.

Loss functions. The F1 score cannot be directly used as the loss function because discretization renders it undifferentiable. A common surrogate is to compute the cross-entropy (CE) or square-error (SE) loss between PPM_{ij} and $Label_{ij}$ for every i-j pair before averaging, which shares

the same global optimum as the F1 score. Notably, the lopsided distribution of negative labels (i.e., 0s) creates an effortless slope towards the local minimum of predicting all zeros for PPM in the early phase of training and a weight bias of 300 for positive labels was used by E2Efold and Ufold to restore the balance. We however found this weight bias or the use of focal loss (9) as done in the image classification to artificially increase the false positives and chose not to apply such weight biases. Instead, we adopt a soft F1 score function as the surrogate loss to directly optimize the F1 score. The soft F1 score is straightforward to implement and was also used by E2Efold, as it simply bypasses the PPM discretization when calculating TP, TN, FP, and FN values, which makes it differentiable.

Staged training. Typical training starts with the CE loss till the F1 score for the VL set stops improving. Then, the loss function is switched to the soft F1 score. This two-stage procedure was found to give the best F1 scores compared with using only one type of loss function.

Hyperparameter tuning. To limit the number of searches, we tuned one hyperparameter at a time while also taking into considerations of the best practices in the literature. The SeqFold2D models of different sizes were tuned separately and the number of epochs for tuning is usually limited to 150 total for efficiency. For example, we fixed dropout to 0.25 and weight decay to 0.01 when tuning the learning rate between 1e-2 and 1e-6. After finalizing the learning rate (usually between 1e-3 and 1e-4), we proceeded to tune dropout between 0.1 and 0.6 and found optimal dropouts to be between 0.2 and 0.42 (usually larger rates for larger models). We did not tune batch size which is set to be the largest allowed by the GPU memory (usually between 8 and 16). The rubric for the best model is based on the F1 score on the validation set.

3. Results

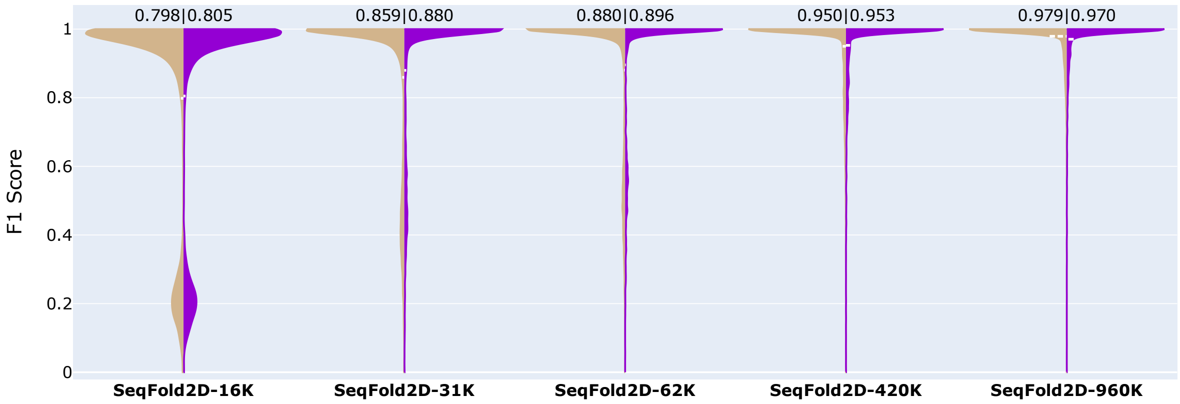

Fig H. The F1 scores of the training (left, tan) and validation (right, violet) sets for several SeqFold2D models developed with the Stralign NR100 (Stral-NR100) dataset randomly split into three subsets: training (TR), validation (VL), and test (TS). The averaged F1 scores are shown at the top and also as dashed lines (white) within the corresponding violin plots (often too narrow to be spotted). Very little TR-VL variances are observed, indicating that the SeqFold2D models are learning the distribution of the entire Stral-NR100 dataset while being trained on the TR subset of the distribution. Note that the F1 scores were saved during training

and all dropout layers were active for the TR set but not for the VL set. These make the F1 scores shown here slightly lower than the values computed without dropout.

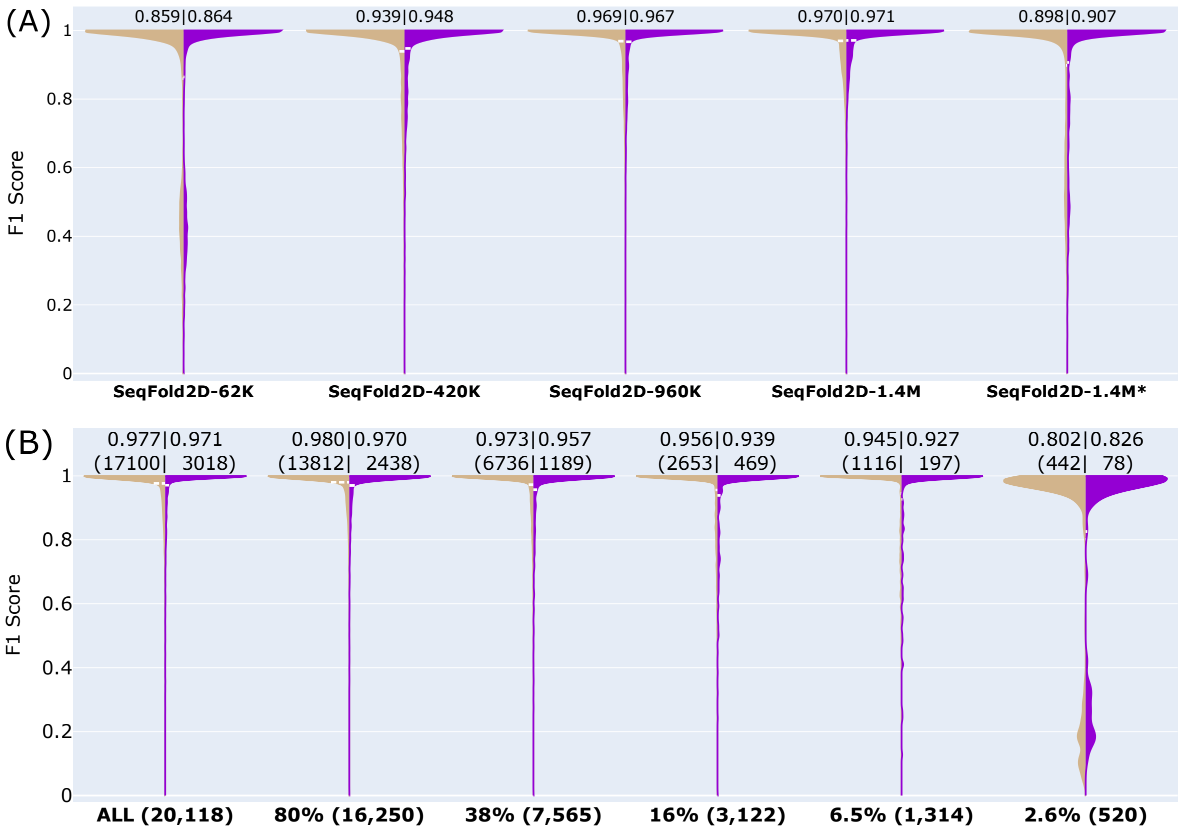

Fig I. The F1 scores of the training (left, tan) and validation (right, violet) sets for the SeqFold2D models developed with the Stralign NR100 (Stral-NR100) dataset randomly split into two subsets only: training (TR) and validation (VL). (A) The performances of SeqFold2D models of different sizes as labelled. Here the entire Stral-NR100 dataset (20,118 sequences) are used for TR and VL. The test set is the ArchiveII NR100 dataset as presented in the main text. The main difference between this set of SeqFold2D models and those in Fig H in S1 Text (with Stral-NR100 split into the TR, VL, and TS sets) is the slightly larger TR set used here, while the training hyperparameters are kept the same for models with the same size. Somewhat surprisingly, this set of models show slightly lower F1 scores for the TR set compared with those shown in Fig H in S1 Text. We do not have good explanations for the drops and did not further investigate the causes as the F1 scores for the VL set are very close. The SeqFold2D-1.4M* model was trained following the similar choices made by E2Efold and Ufold, specially with the cross-entropy loss function only and a weight of 300 for positive labels. As the shown TR and VL F1 scores were saved during training without post-processing, the scores from the SeqFold2D-1.4M* model are significantly lower than that after post-processing. For example, the averaged F1 score for the TR set increases from 0.898 to 0.981 with post processing for SeqFold2D-1.4M*. (B) The dependence of model performance (SeqFold2D-420K) on the size of the seen dataset (TR and VL) denoted in the x axis labels. Random sampling of the parent dataset (Stral-NR100) is used here, in contrast with the similarity-based de-redundancy method with CD-HIT-EST. A gradual decrease of model performance is observed as the data size decreases.

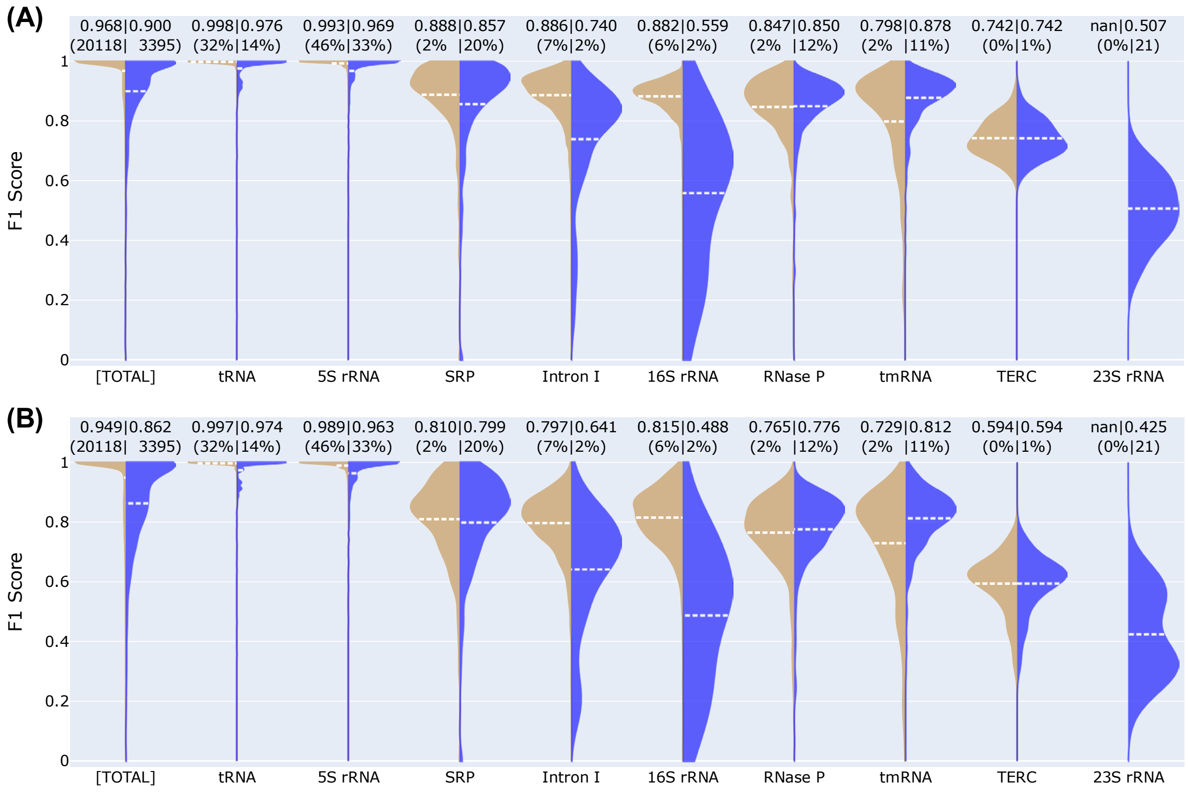

Fig J. The F1 scores of the TR+VL set (Stralign NR100, left in tan) and the TS set (ArchiveII NR100, right in blue) for the Ufold model with (A) and without (B) post-processing. The leftmost pair of violins show the F1 scores for the entire sets and the following violin pairs show each constituent RNA family. Averaged scores are shown at the very top and also as dashed lines (white) within the violins. The values in the parentheses above are the sequence counts in actual numbers (for the whole set or families with <1% shares) or in percentages (for families with >1% shares). Note that 23S rRNA only exists in ArchiveII NR100.

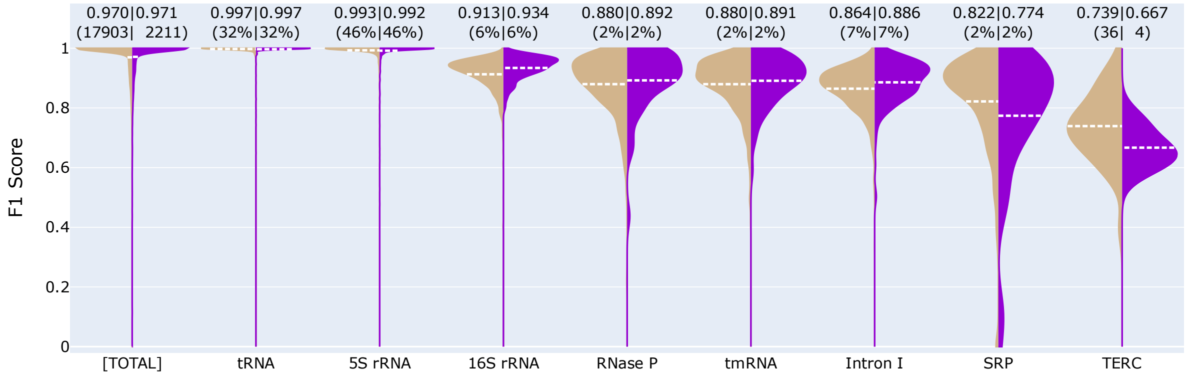

Fig K. The F1 scores of the TR (left, tan) and VL (right, violet) sets for SeqFold2D-1.4M developed with the Stralign NR100 (Stral-NR100) dataset randomly split into TR and VL sets. It is the same SeqFold2D-1.4M model shown in Fig I in S1 Text. No significant TR-VL variances (i.e., overfitting) are observed for the whole set or individual RNA families.

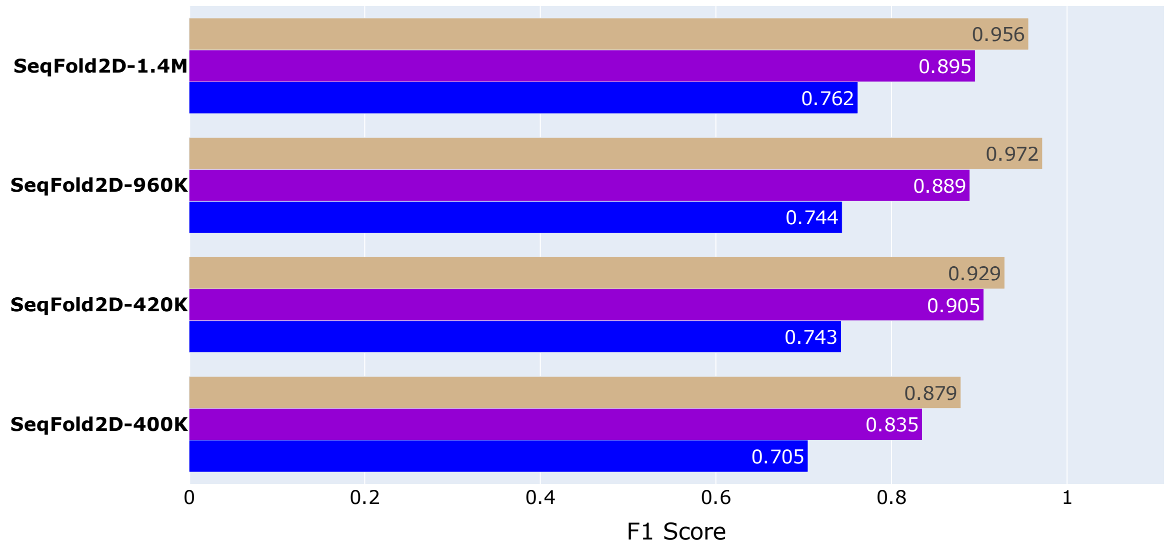

Fig L. The F1 scores of the TR (top, tan), VL (middle, violet), and TS (bottom, blue) sets for the SeqFold2D models developed with Stral-NR80 as TR and VL and Archi-Stral-NR80 as TS. All SeqFold2D models exhibit significant TR-VL variances (i.e., overfitting), while still attaining decent performances over the TS set. The two smallest models (400K and 420K) have design variables of (N=3, C=48) and (N=7, C=32), respectively. It is worth noting that increasing the number of parameters from 960K to 1.4M did not increase the performances on the TR and VL sets but resulted in slightly better performances on the TS set.

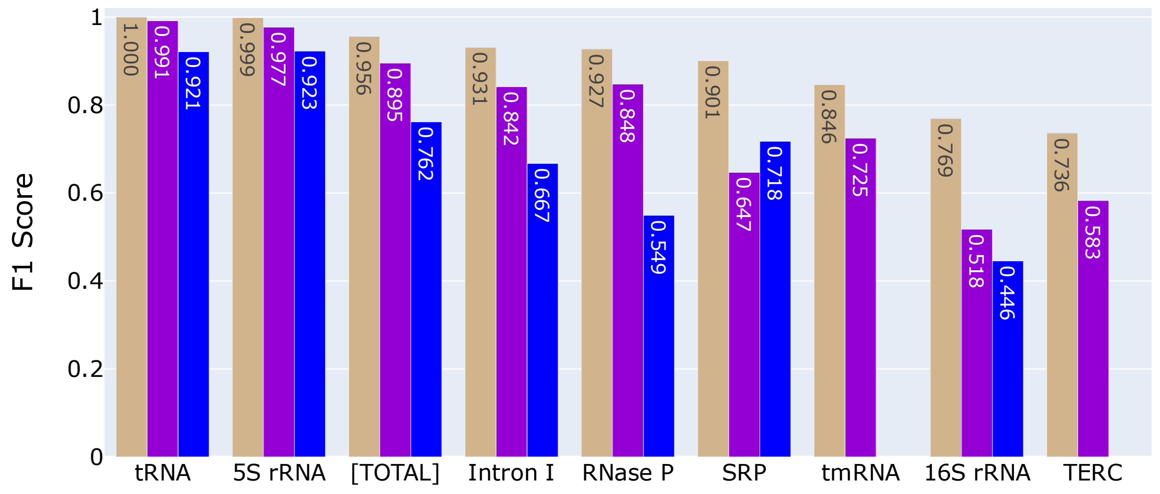

Fig M. The F1 scores of the TR (tan, left), VL (violet, middle), and TS (blue, right) sets on the entire ([TOTAL]) and individual RNA families for the same SeqFold2D-1.4M model as shown in Fig L in S1 Text. The order along the *x* axis follows the F1 scores of the TR set. Note that the TS set does not have tmRNA or TERC sequences after removing sequences with above 80% similarity with the Stral-NR80 dataset. The main observation is that large TR-VL and TR-TS variances are observed for all RNA families and that the TR-TS variance is usually much larger than the corresponding TR-VL variance except for the SRP family.

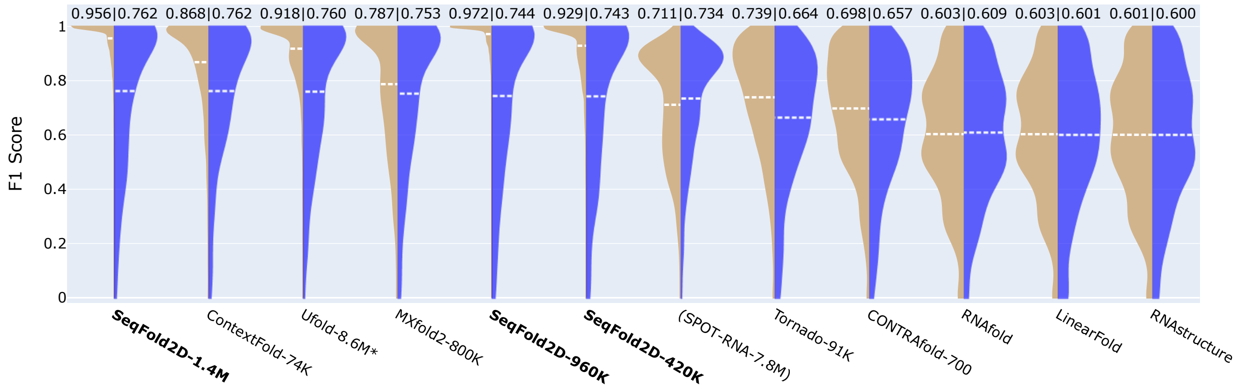

Fig N. Visualization of the TR (Stral-NR80) vs. TS (Archi-Stral-NR80) gaps for SeqFold2D and selected DL, ML, and physics-based models. The models are ordered by the TS F1 score. We retrained five models (Ufold, MXfold2, ContextFold, Tornado, and ContraFold) but failed to retrain SPOT-RNA. It should be noted that we were unable to reproduce the same levels of performance for the DL models (Ufold and MXfold2) as their published parameters when using the same datasets (Stral-NR100 or bpRNA). As such, the performances of the DL models shown here do not represent their true capabilities and should be considered as for reference only. Note that the physics-based LinearFold-C is used in this study, while the LinearFold-C is based on the ContraFold parameters and thus expected to perform similarly to Contrafold if retrained.

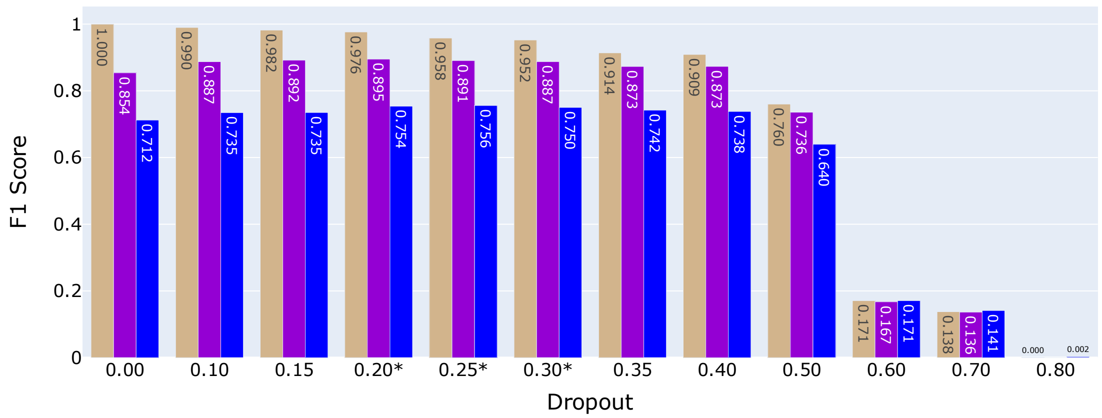

Fig O. Illustration of the effect of dropout rates on the performance and generalization of the SeqFold2D-960K model with the TR and VL sets derived from Stral-NR80 and Archi-Stral-NR80 as TS. Shown for each dropout rate are the F1 scores of the TR (left, tan), VL (middle, violet), and TS (right, blue) sets. In terms of performance, the TR F1 score steadily decreases with increasing dropout rate and, interestingly, the VL and TS F1 scores peak around the same rate between 0.2 and 0.3 (as adopted by the final SeqFold2D models). As for generalization, zero dropout leads to largest TR-VL and TR-TS variances and the dropout rates above 0.5 reduce both to zero. While regularization can indeed tune both performance and generalization, the two metrics are conflicting with each other and one has to balance them in accord to the needs. Note that the optimal dropout is expected to depend on the exact sequence distributions, as well as other model parameters.

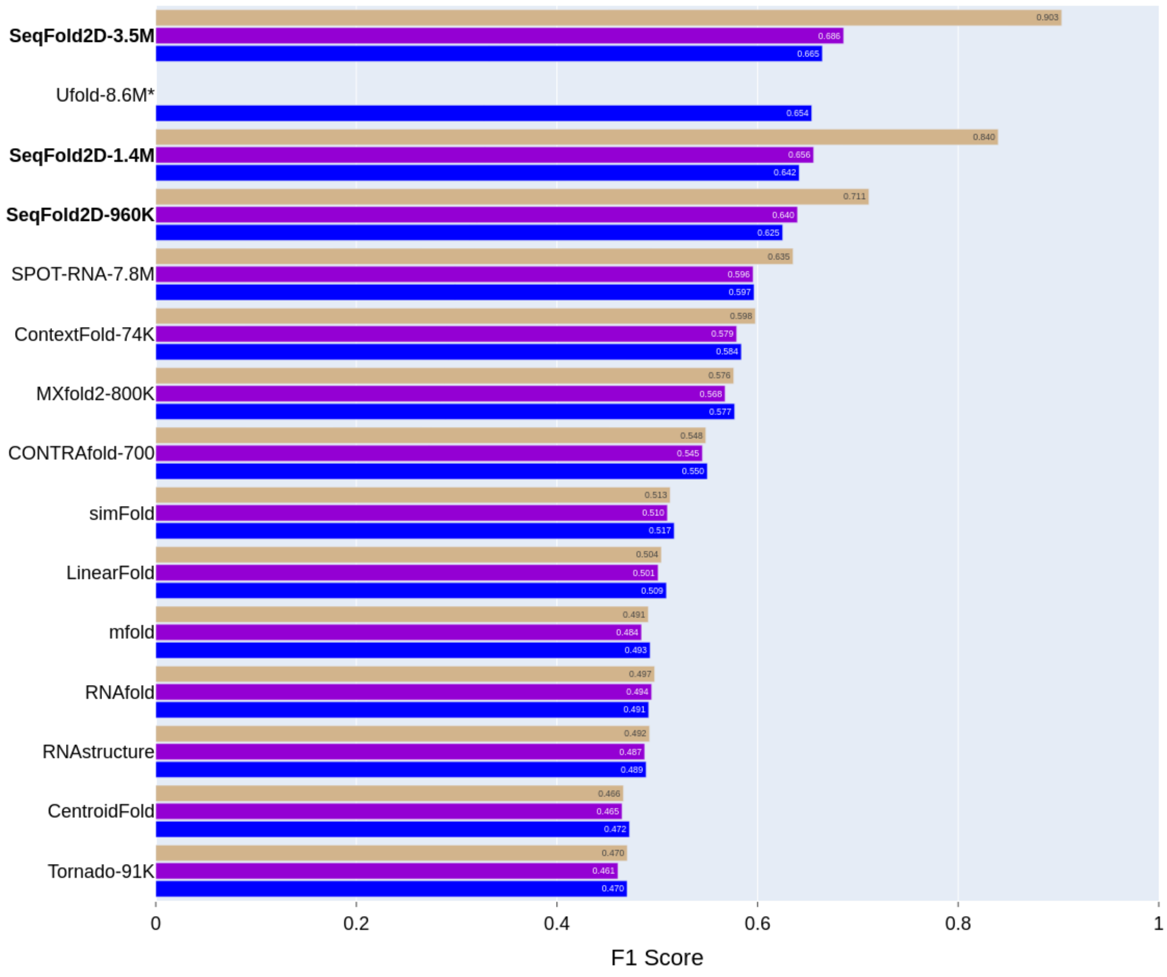

Fig P. The F1 scores of the training (top, tan), validation (middle, violet), and test (bottom, blue) sets for selected DL, ML, and physics-based models. Here the training, validation, and test sets are the bpRNA TR0, VL0, and TS0 datasets compiled by the SPOT-RNA team, respectively. The three datasets are expected to have independent, identical distributions, which are reflected by their comparable prediction performances by traditional algorithms. As discussed in the main text, the SeqFold2D models were trained to optimize the performance on the validation set, regardless of the magnitude of the train-validation variances. Ufold does not provide the saved model parameters trained with the bpRNA dataset, and thus only the value for the bpRNA TS0 set is available from the Ufold article (10). Notably, rather decent F1 scores can be achieved on the bpRNA TR0 set, rapidly improving from 0.711 to 0.840 to 0.903 for the SeqFold2D-960K, 1.4M, and 3.5M models, respectively, but this results in rather small gains on the TS0 set (0.625, 0.642, and 0.665, correspondingly). The generalization gap can be reduced by model regularization which again fails to achieve both performance and generalization as shown in Fig O in S1 Text for the case of dropout rate. We further note that the SeqFold2D models show even worse generalizability for the bpRNA-NEW dataset and we plan to use data augmentations techniques demonstrated by Ufold to improve generalizability in future work.

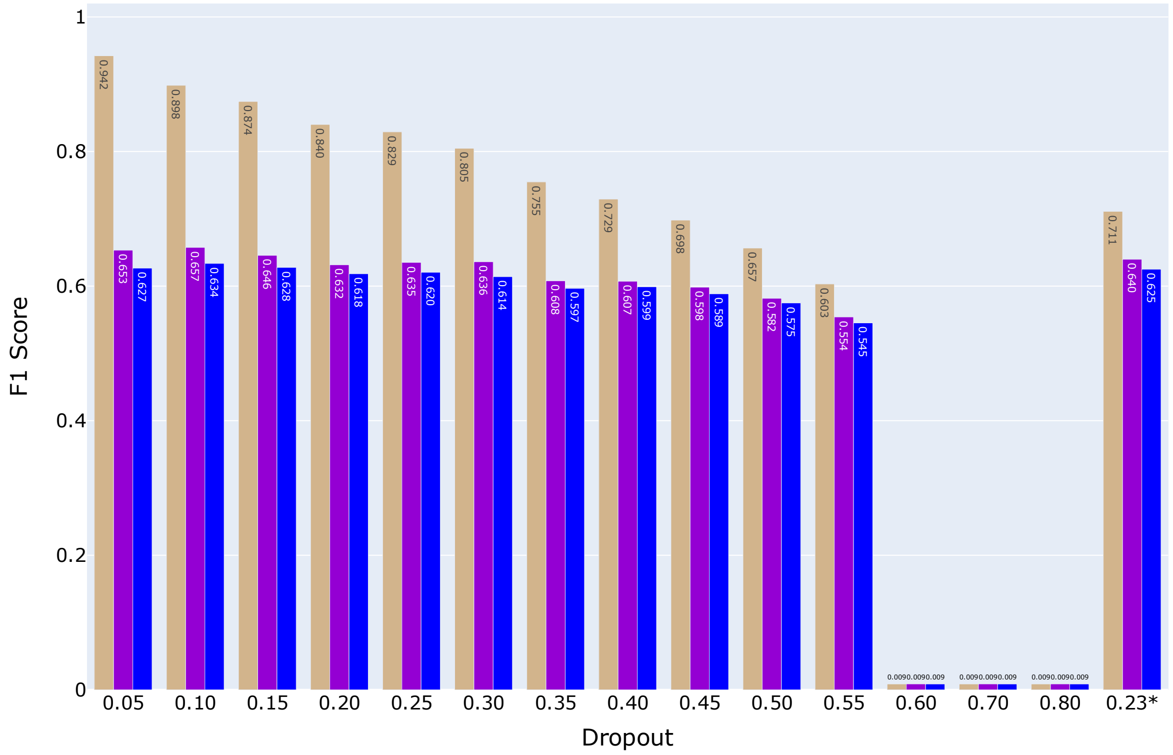

Fig Q. The scan of dropout rates for the SeqFold2D-960K model with the bpRNA TR0, VL0, and TS0 datasets. The observations are in qualitative agreement with the dropout scan with the Stral-NR80 and Archi-Stral-NR80 datasets shown in Fig N in S1 Text. The training set (TR0) F1 score decreases monotonically with the dropout rate; the validation and test scores peak around relatively low dropout rates ~0.10. The TR0-TS0 gap does decrease with the increase of dropout but high dropout rates lead to very low absolute performance. The rightmost set (0.23*) shows the final SeqFold2D-960K model after additional optimizations of performance and generalizability tradeoffs.

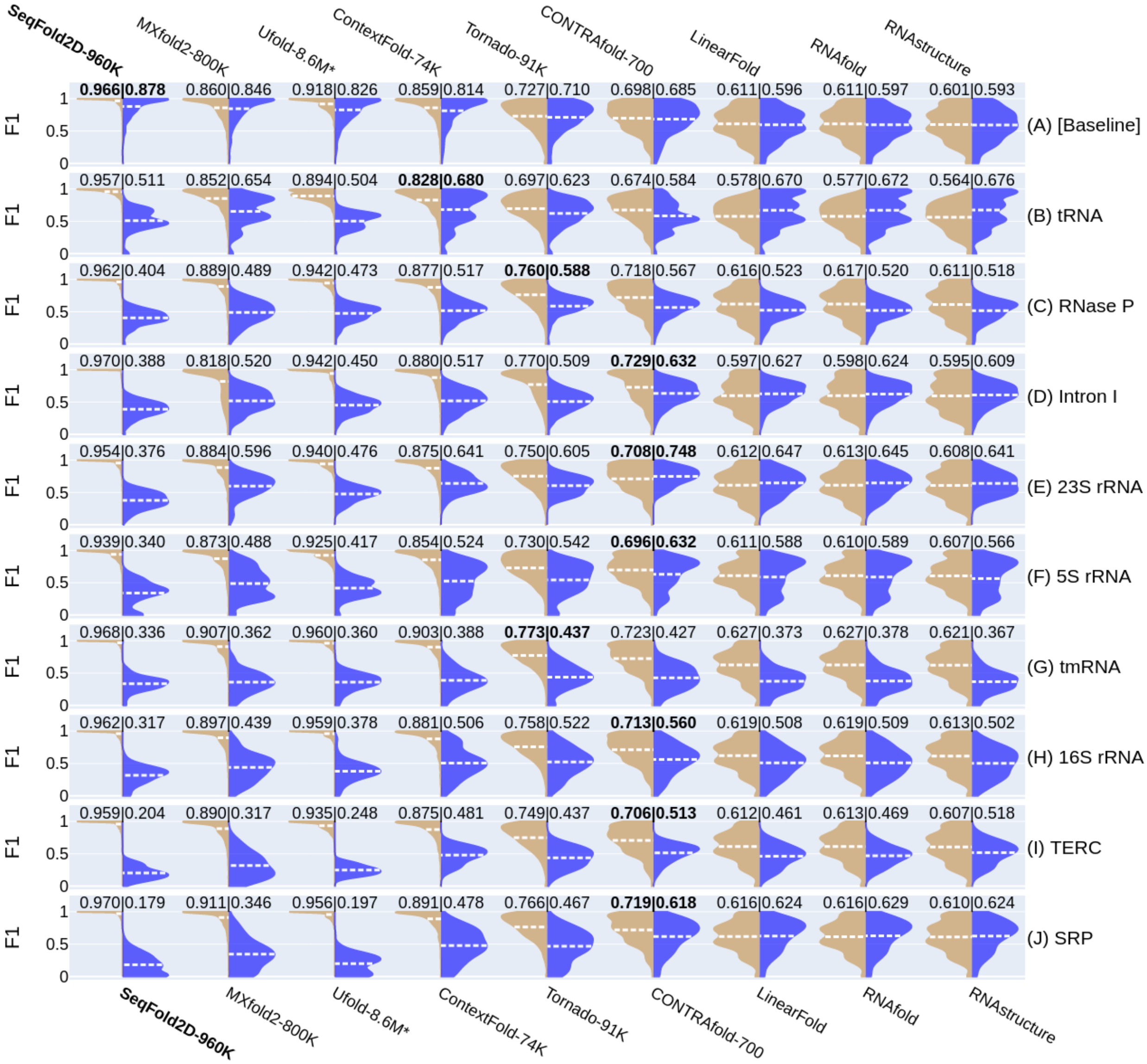

Fig R. Illustrations of the TR (left, tan) vs. TS (right, blue) performances at the cross-family level with the Strive-NR80 dataset. This is an extended plot of Fig 4 in the main text by showing all nine cross-family studies. Detailed captioning follows that of Fig 4 in the main text as well.

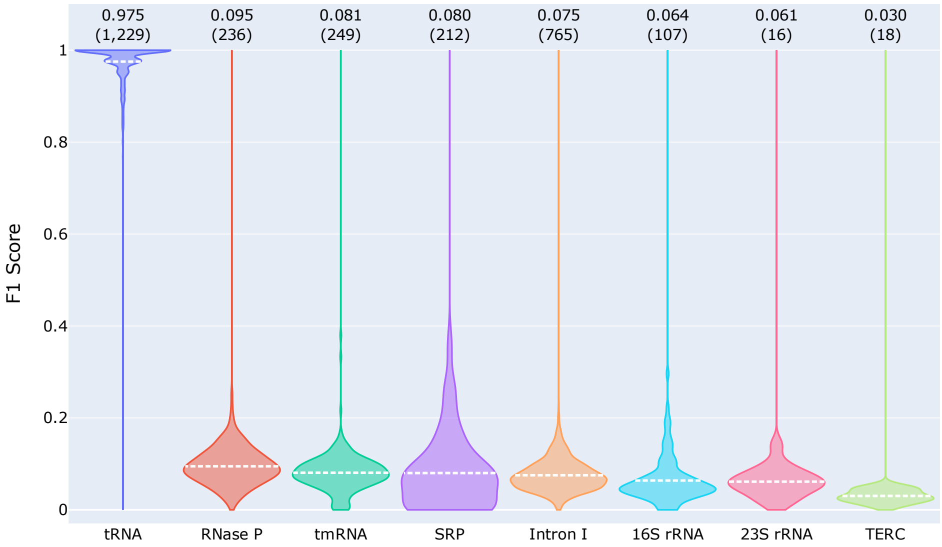

Fig S. The cross-family study with tRNA as the TR and VL sets and all other families as the TS set. The DL model is SeqFold2D-400K and the parent dataset is Strive-NR80. Note that model training was stopped when the TR-VL variance became significant for this study. While the model displays excellent performances over the seen sequences (the first violin), the performances over other family types fail completely.

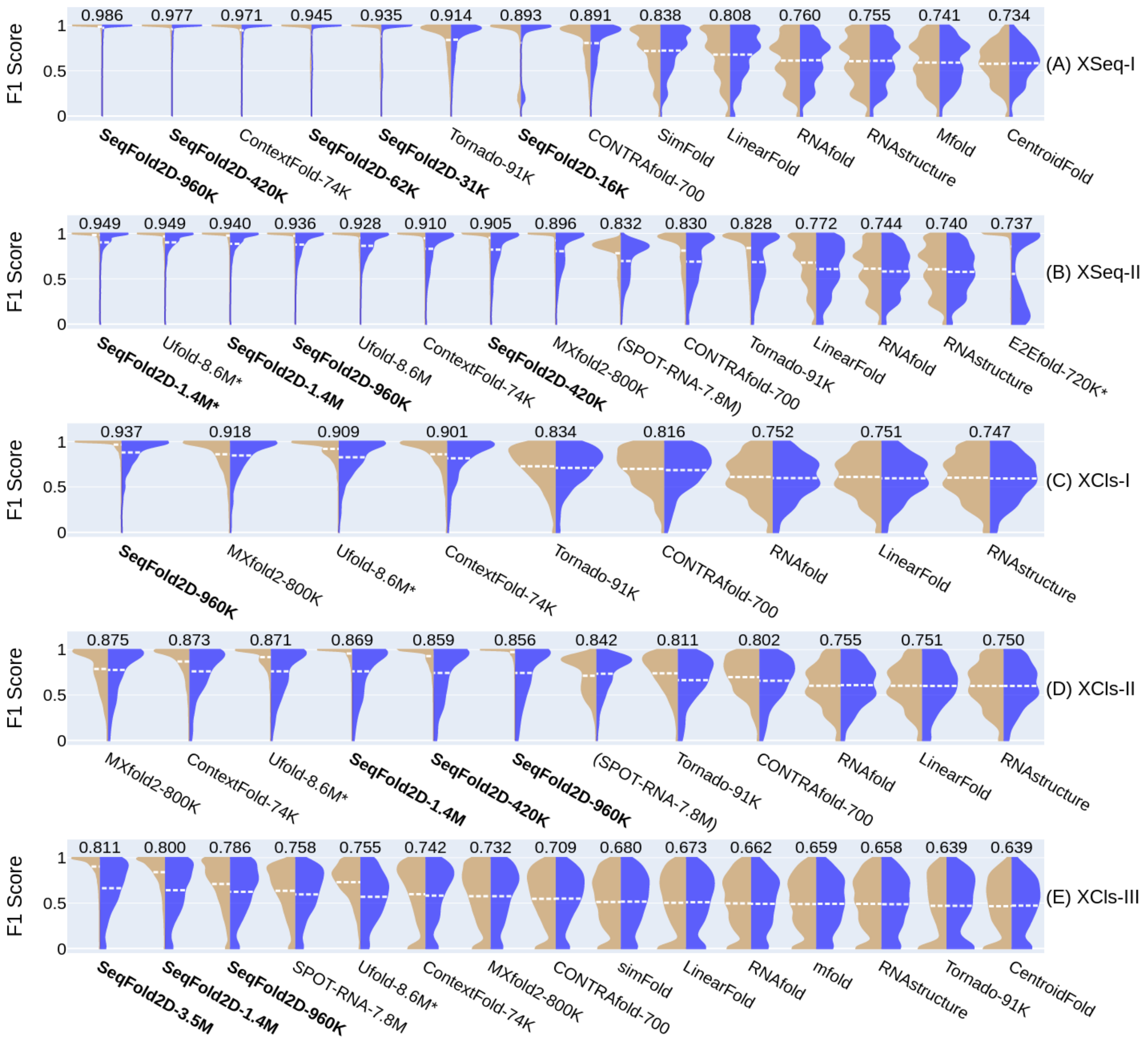

Fig T. Illustration of the PGscores of all cross-sequence and cross-cluster studies presented in this work. Each row shows one study as labelled to the right. The models are sorted by the PGscore in descending order from left to right. For each model, the pair of violins show the F1 score distributions of TR (left, tan) and TS (right, blue) with its PGscore shown above. The names of the studies follow that in Fig 5B in the main text. Specifically, (A) XSeq-I: the cross-sequence study with Stral-NR100 only, (B) XSeq-II: cross-sequence with Stral-NR100 and Archi-NR100, (C) XCls-I: cross-cluster with Strive-NR80 only, (D) XCls-II: cross-cluster with Stral-NR80 and Archi-Stral-NR80, (E) XCls-III: cross-cluster with bpRNA. The cross-family studies are shown in Fig U in S1 Text.

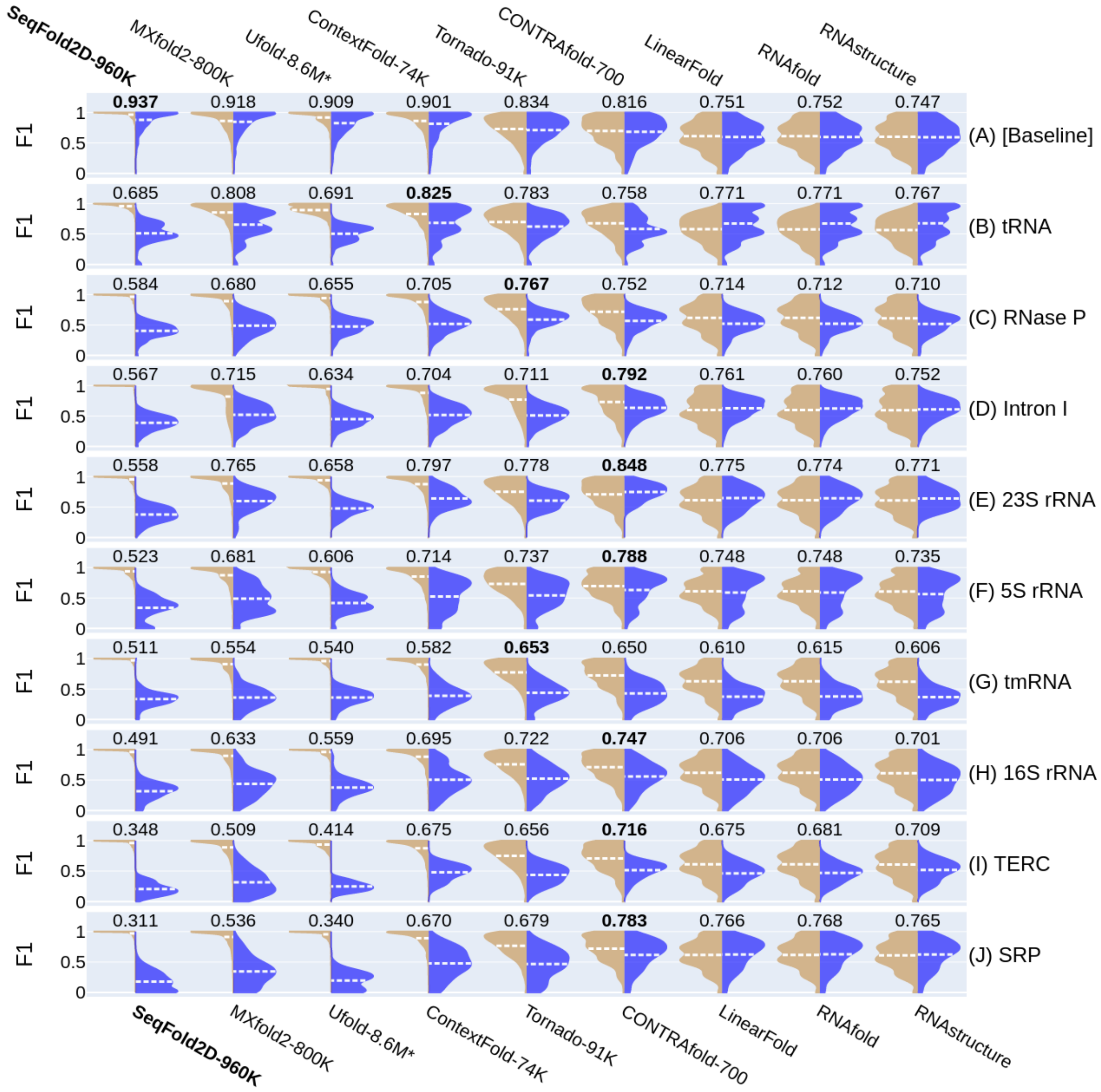

Fig U. Illustration of the PGscores of all cross-family studies presented in this work. Each row shows one study as labelled to the right. The first row is the base-line cross-cluster study with Strive-NR80 (the same as (C) XCls-I in Fig T in S1 Text). For each model, the pair of violins show the F1 score distributions of TR (left, tan) and TS (right, blue) with its PGscore shown above. The highest PGscore among the learning-based models (the first six models) is shown in bold.

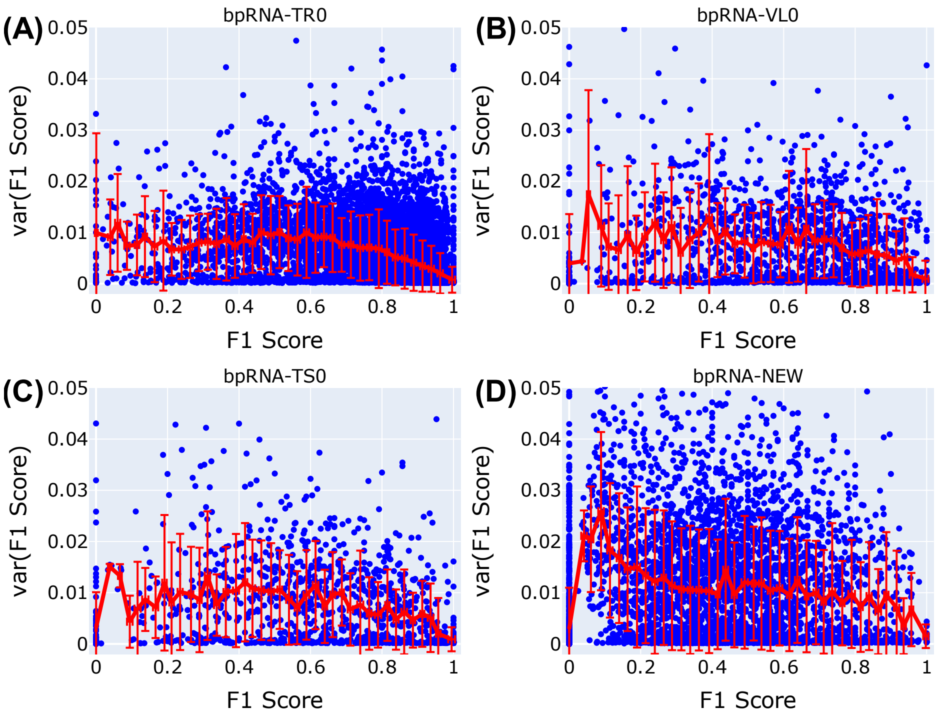

Fig V. The correlations between the estimated variances and the actual values of the F1 scores on the training (A, bpRNA TS0), validation (B, bpRNA VL0), test (C, bpRNA TS0), and another independent test (D, bpRNA-New) datasets for the SeqFold2D-960K model.

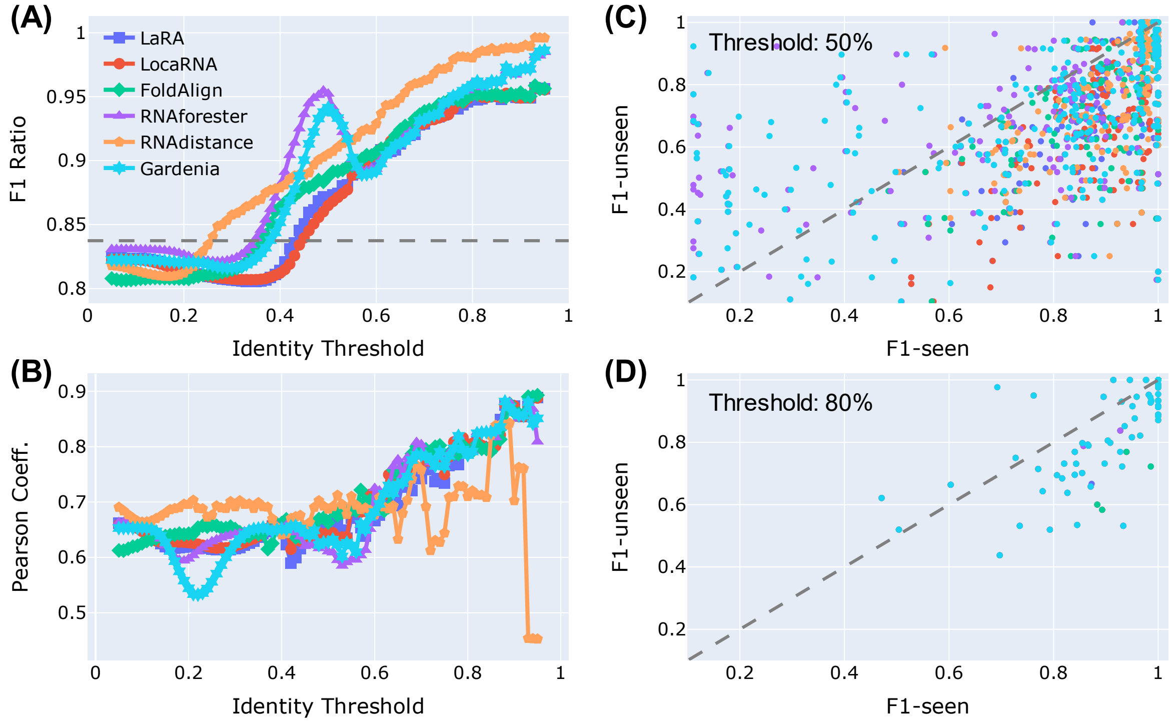

Fig W. Illustrations of the correlation between the F1-unseen and F1-seen scores of the Ufold-8.6M* model. Captioning follows that of Fig 6 in the main text.

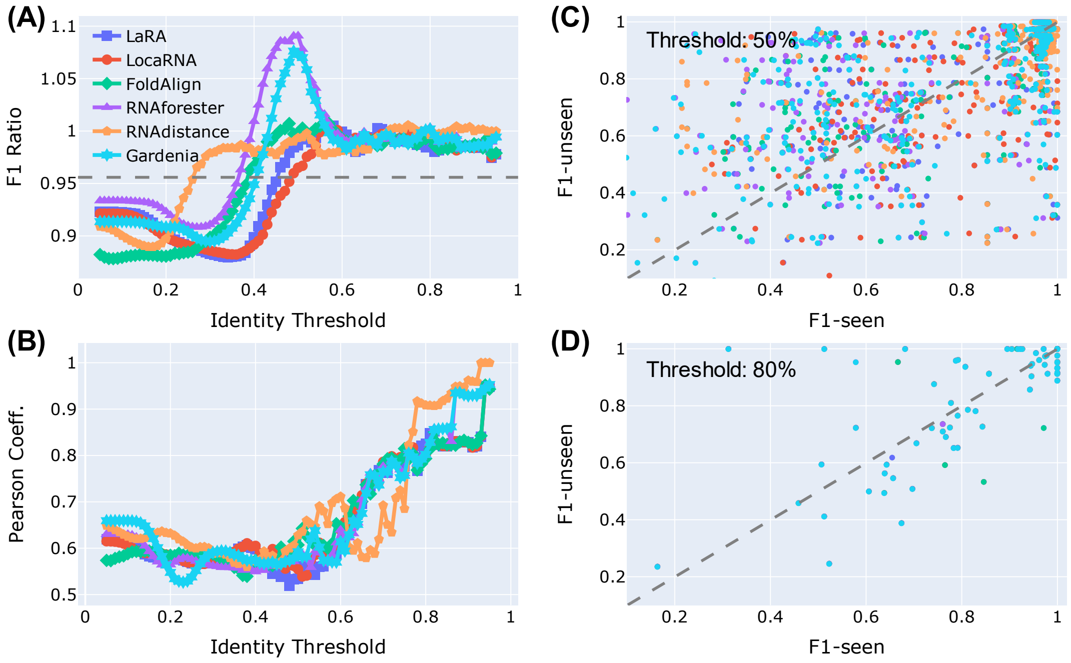

Fig X. Illustrations of the correlation between the F1-unseen and F1-seen scores of the MXfold2-800K model. Captioning follows that of Fig 6 in the main text. Note that we were only able to re-train MXfold2 on Stral-NR80 to attain the F1 score of 0.797, far below the F1~0.922 for Stral-NR100 attained by the published model (Fig 2B in the main text). Thus the shown MXfold2 model appears under-retrained, leading to poor performance and excellent generalization.

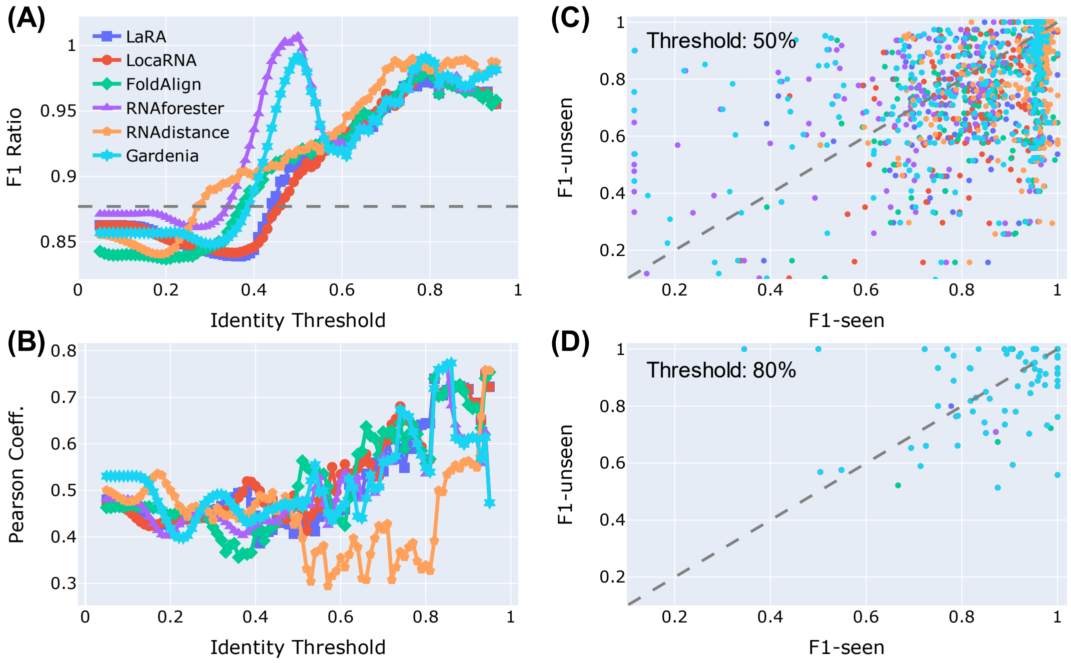

Fig Y. Illustrations of the correlation between the F1-unseen and F1-seen scores of the ContextFold-74K model. Captioning follows that of Fig 6 in the main text.

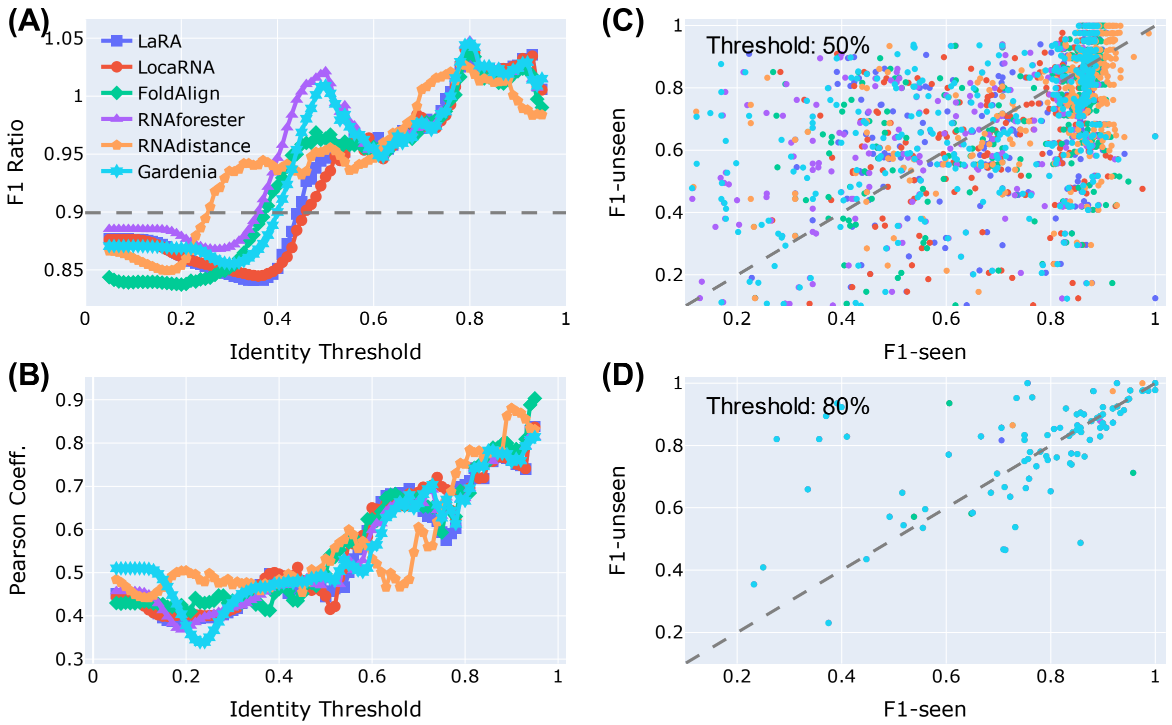

Fig Z. Illustrations of the correlation between the F1-unseen and F1-seen scores of the Tornado-91K model. Captioning follows that of Fig 6 in the main text.

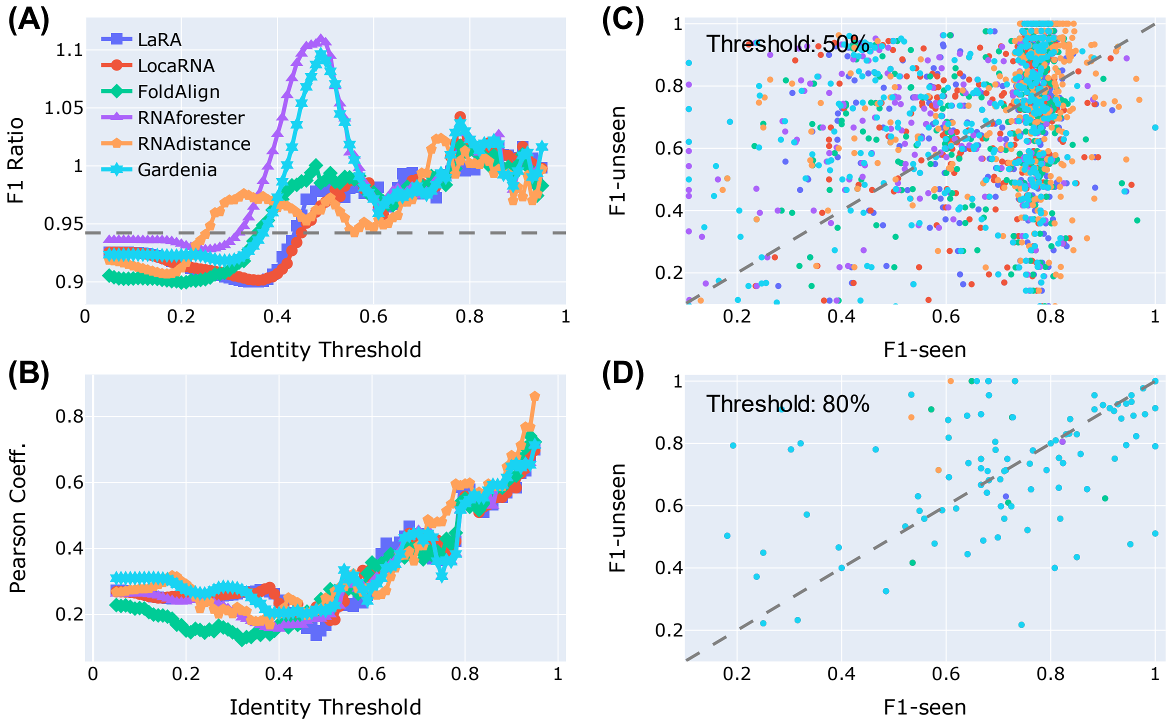

Fig AA. Illustrations of the correlation between the F1-unseen and F1-seen scores of the CONTRAfold-700 model. Captioning follows that of Fig 6 in the main text.

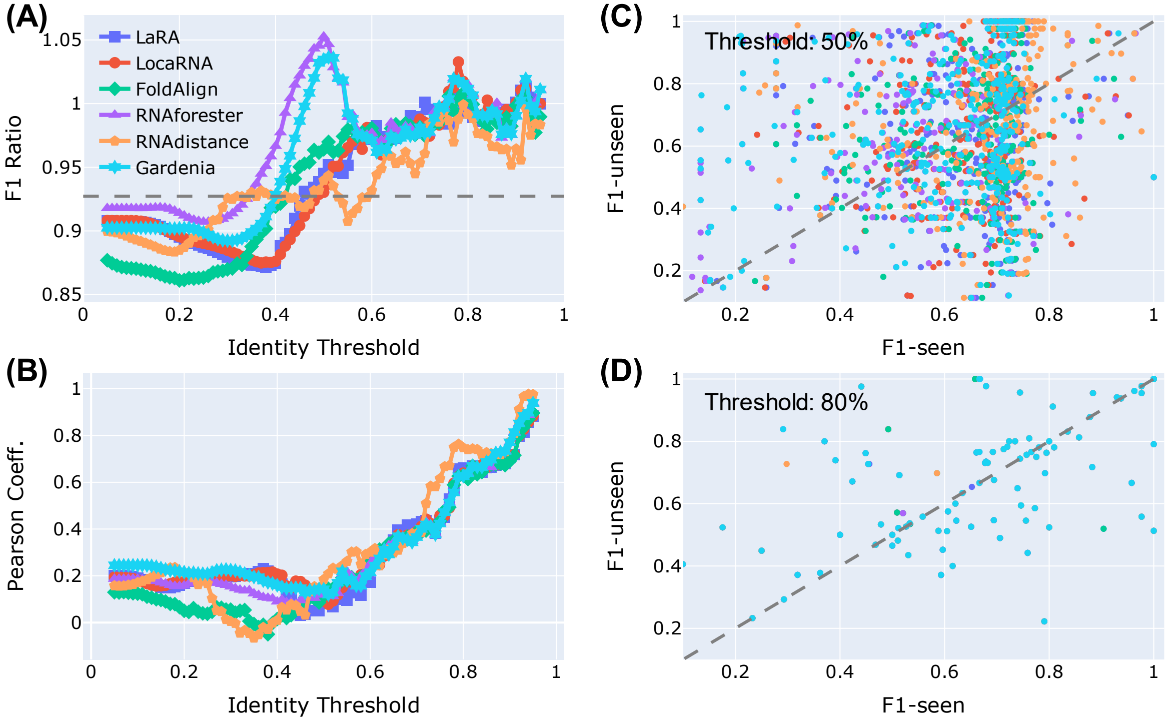

Fig BB. Illustrations of the correlation between the F1-unseen and F1-seen scores of the LinearFold model. Captioning follows that of Fig 6 in the main text.

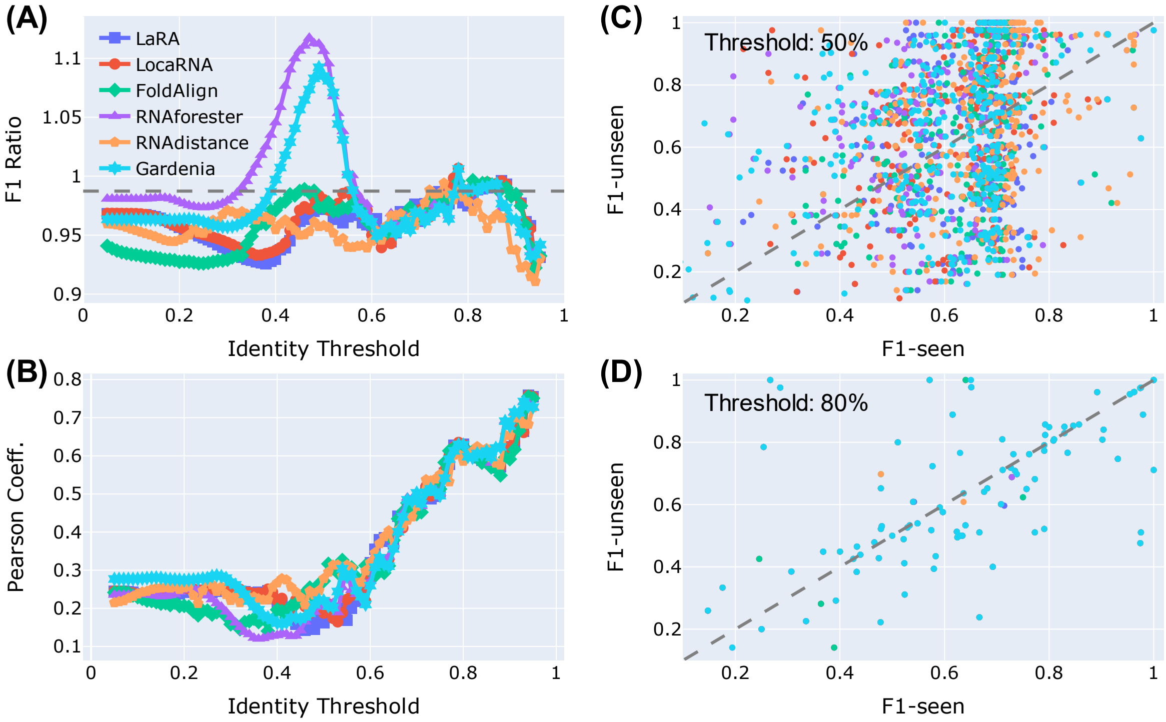

Fig CC. Illustrations of the correlation between the F1-unseen and F1-seen scores of the RNAstructure model. Captioning follows that of Fig 6 in the main text.

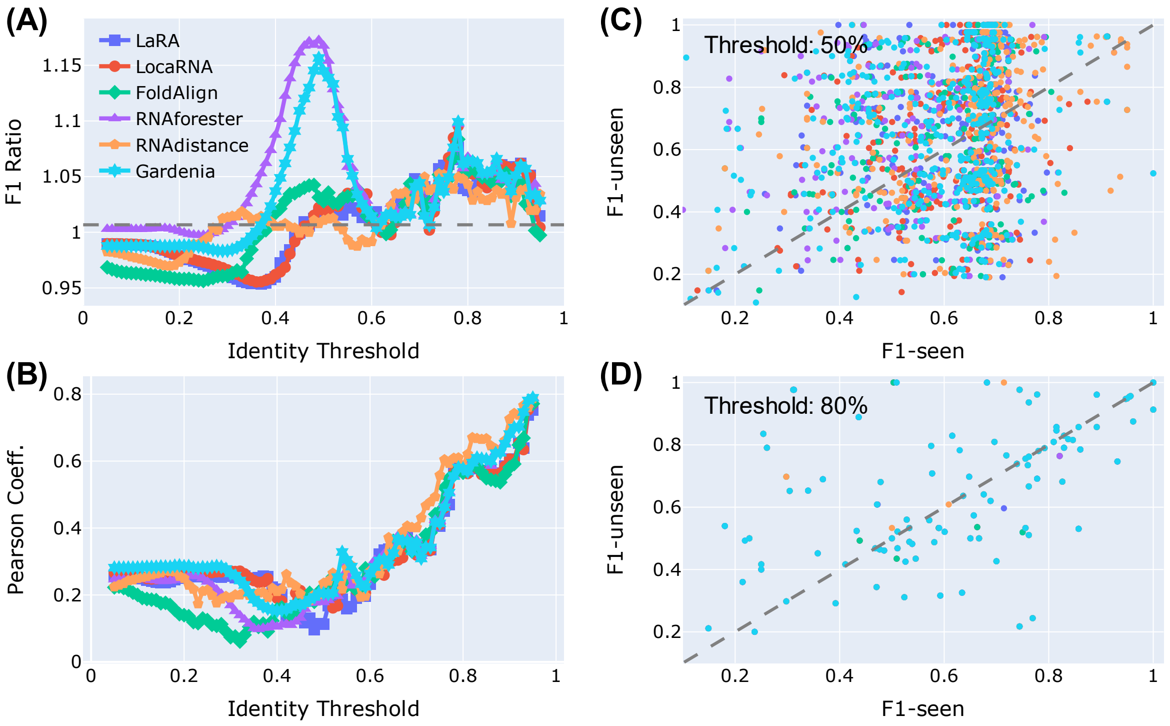

Fig DD. Illustrations of the correlation between the F1-unseen and F1-seen scores of the RNAfold model. Captioning follows that of Fig 6 in the main text.

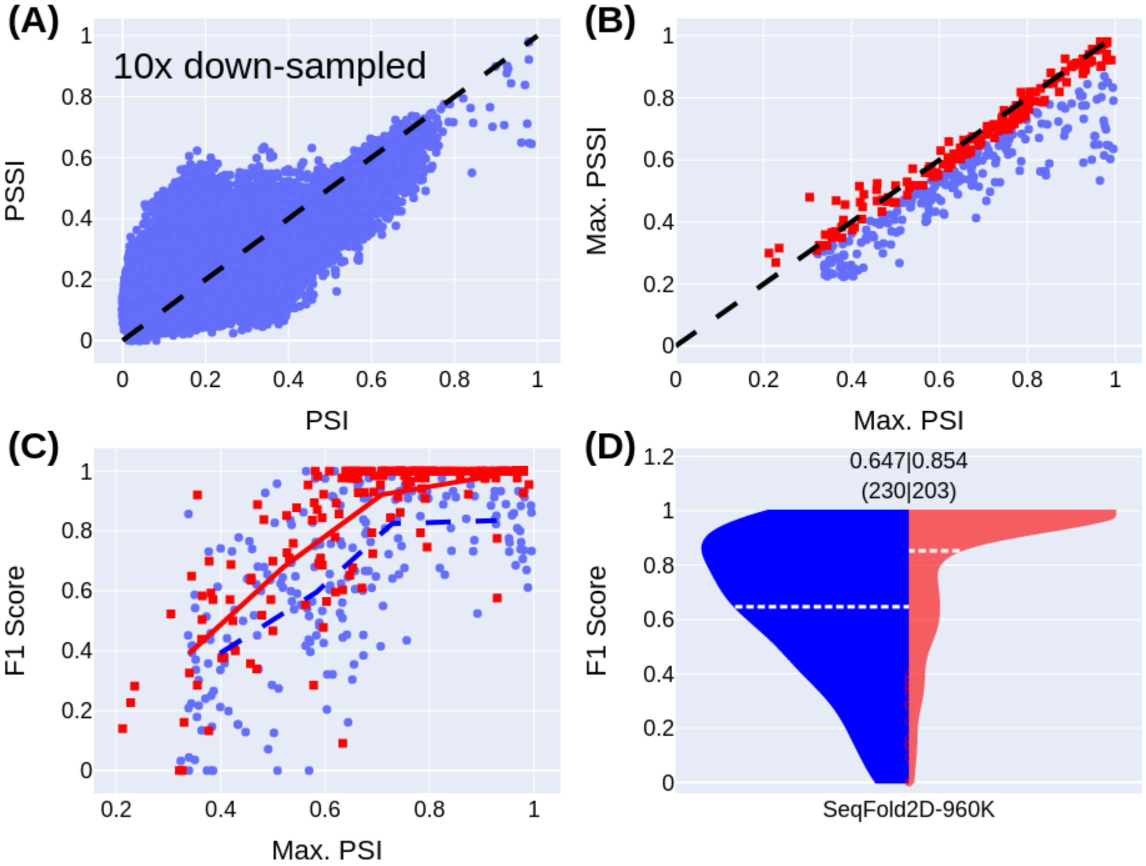

Fig EE. Comparisons of the pairwise PSI (FoldAlign) and PSSI (RNAdistance) scores and the dependences of SeqFold2D-960K performances on the similarities in RNA sequence and structure. Both PSI and PSSI scores are from the pairwise alignments between the unseen set (Archi-Stral-NR80, 433 RNAs) and the seen set (Stral-NR80, 3122 RNAs). (A) Scatter plot of the PSI vs. PSSI score for each unseen-seen pair (1,351,826 total, down-sampled by a factor of 10). (B) Scatter plot of the maximum PSI vs. maximum PSSI score for each unseen RNA molecule. Note that the maximum PSI and PSSI scores may be obtained from a different seen sequence/structure. The unseen sequences are divided into two groups (blue and red) of comparable sizes by the PSI/PSSI score ratio. One group (blue circles) has PSI/PSSI ratios > 1.08, representing the low structure similarity population, while the other (red squares) has ratios < 1.08, representing the high structure similarity population. (C) The F1 score of the unseen sequence shown against its maximum PSI score, grouped by low (blue circles) and high (red squares) structure similarities as in (B). The blue dashed line and the red solid line show the average F1 scores as a function of the maximum PSI score for the low and high structure similarity groups, respectively. (D) Violin plot of the F1 score distribution of the low (blue, left) and high (red, right) structure similarity groups. The average F1 score for each group is shown at the top with the number of sequences shown beneath.

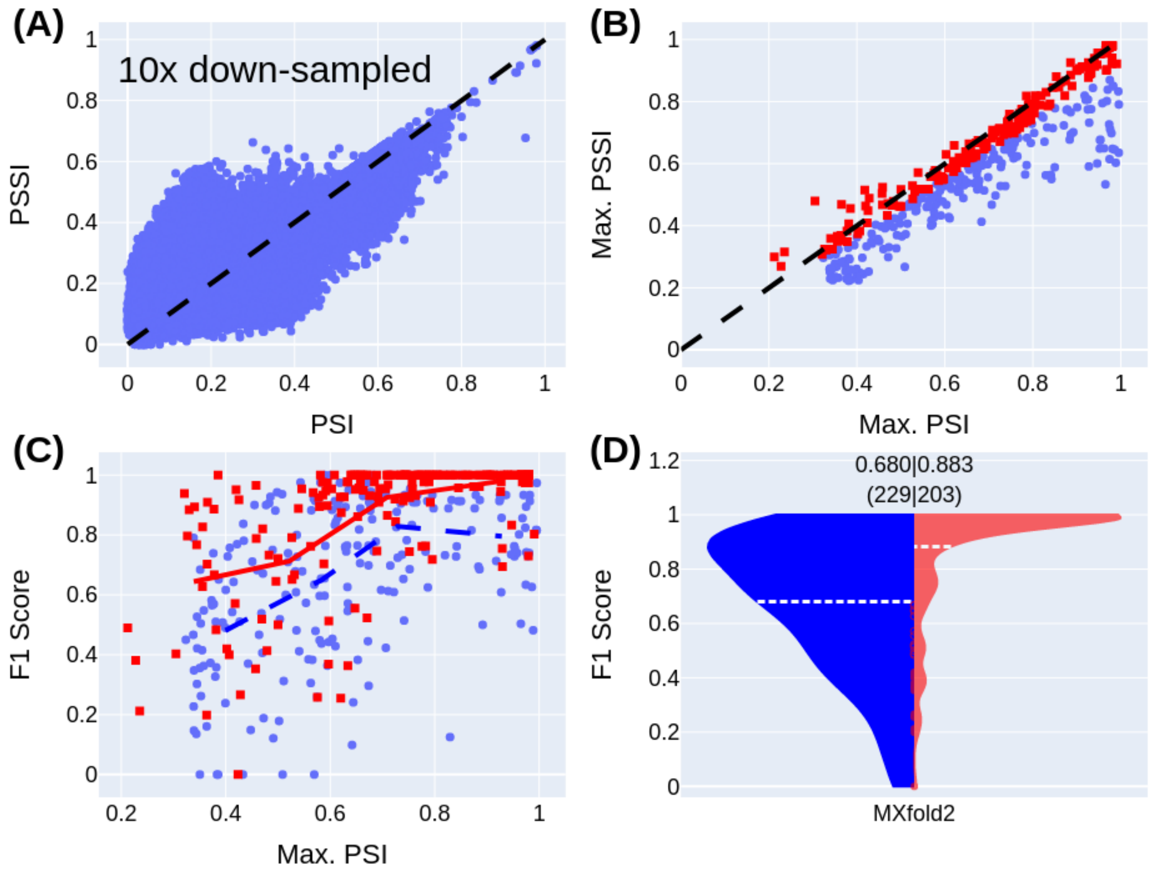

Fig FF. Comparisons of the pairwise PSI (FoldAlign) and PSSI (RNAdistance) scores and the dependences of MXfold-800K performances on the similarities in RNA sequence and structure. Description of each panel follows that of Fig EE in S1 Text. Note that the model is retrained with the Stral-NR80 dataset by us.

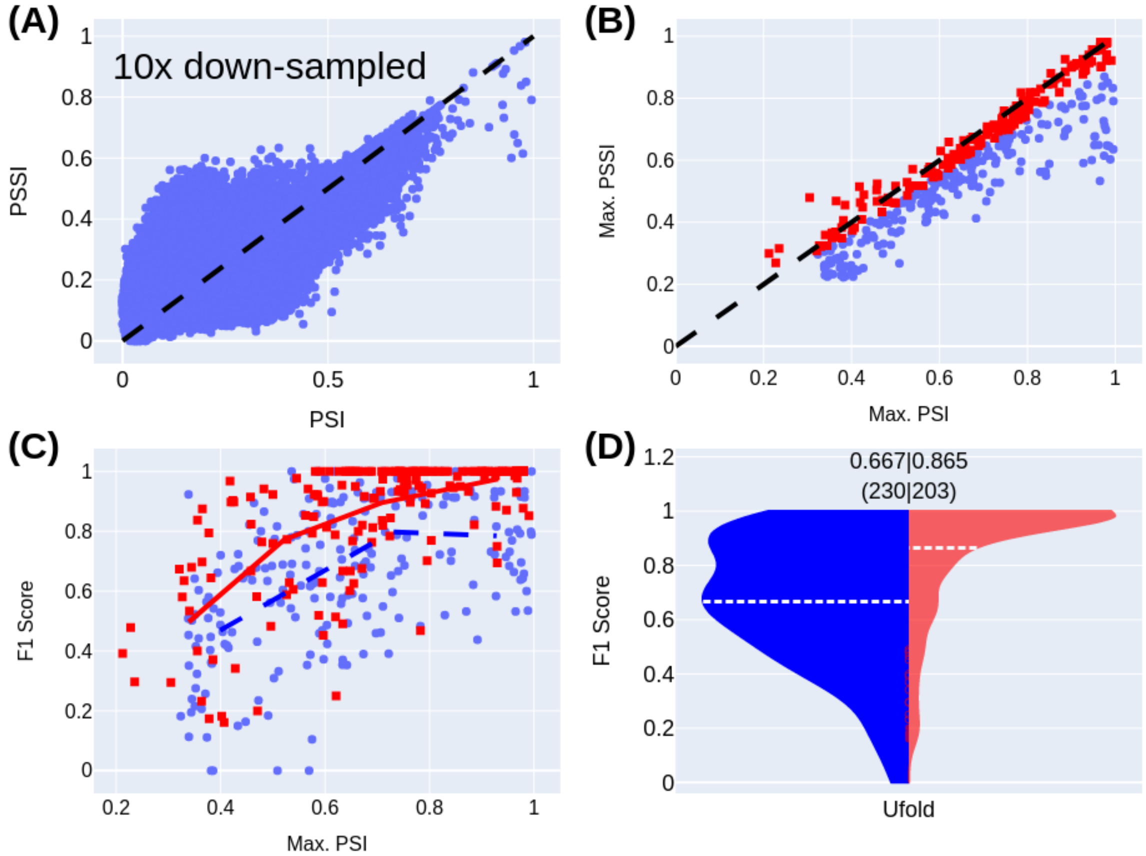

Fig GG. Comparisons of the pairwise PSI (FoldAlign) and PSSI (RNAdistance) scores and the dependences of Ufold-8.6M performances on the similarities in RNA sequence and structure. Description of each panel follows that of Fig EE in S1 Text. Note that the model is retrained with the Stral-NR80 dataset by us.

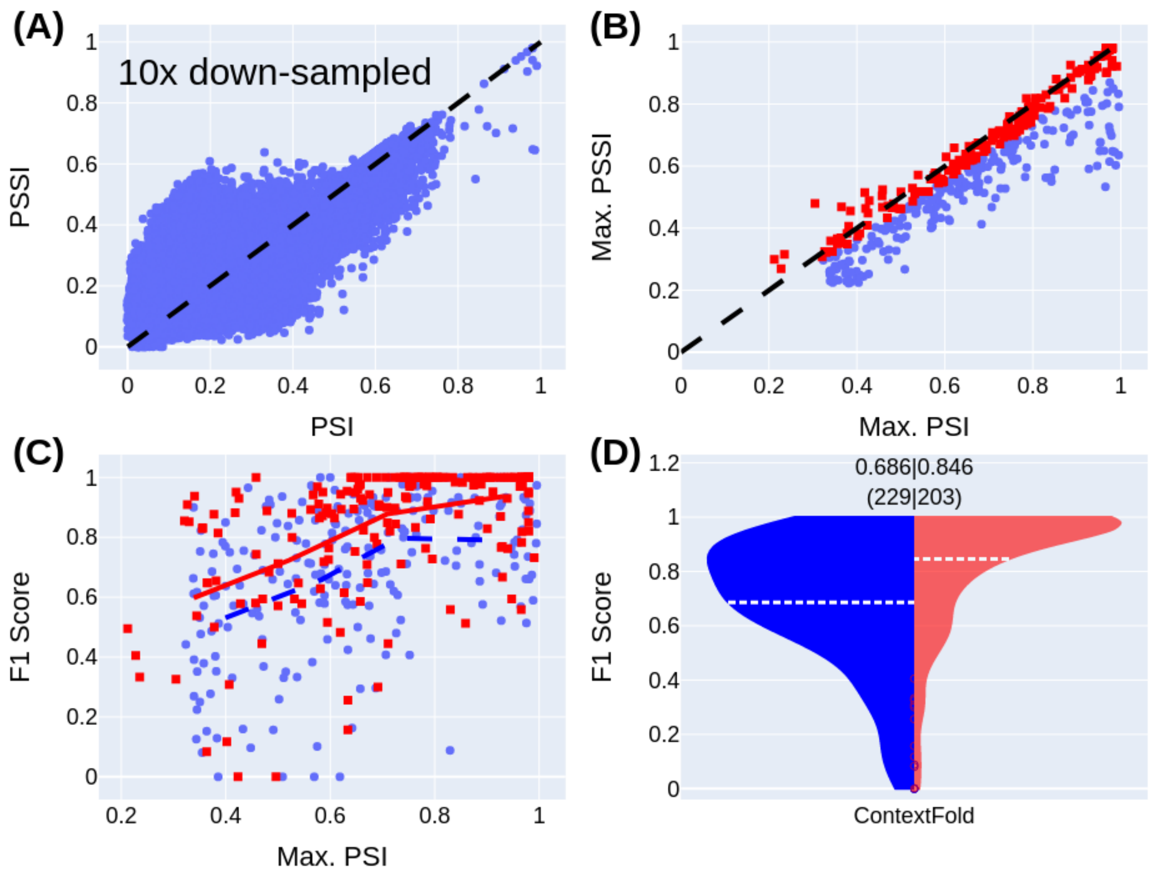

Fig HH. Comparisons of the pairwise PSI (FoldAlign) and PSSI (RNAdistance) scores and the dependences of ContextFold-74K performances on the similarities in RNA sequence and structure. Description of each panel follows that of Fig EE in S1 Text. Note that the model is retrained with the Stral-NR80 dataset by us.

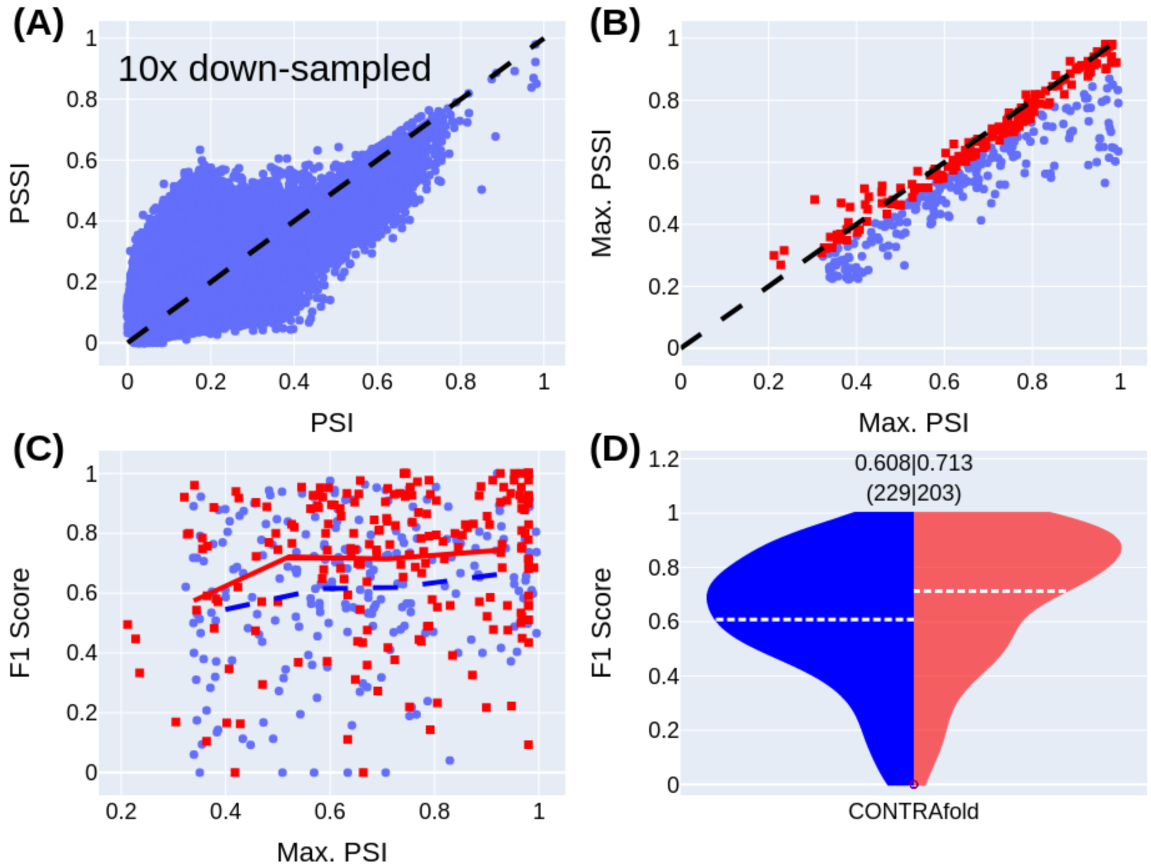

Fig II. Comparisons of the pairwise PSI (FoldAlign) and PSSI (RNAdistance) scores and the dependences of CONTRAfold-700 performances on the similarities in RNA sequence and structure. Description of each panel follows that of Fig EE in S1 Text. Note that the model is retrained with the Stral-NR80 dataset by us.

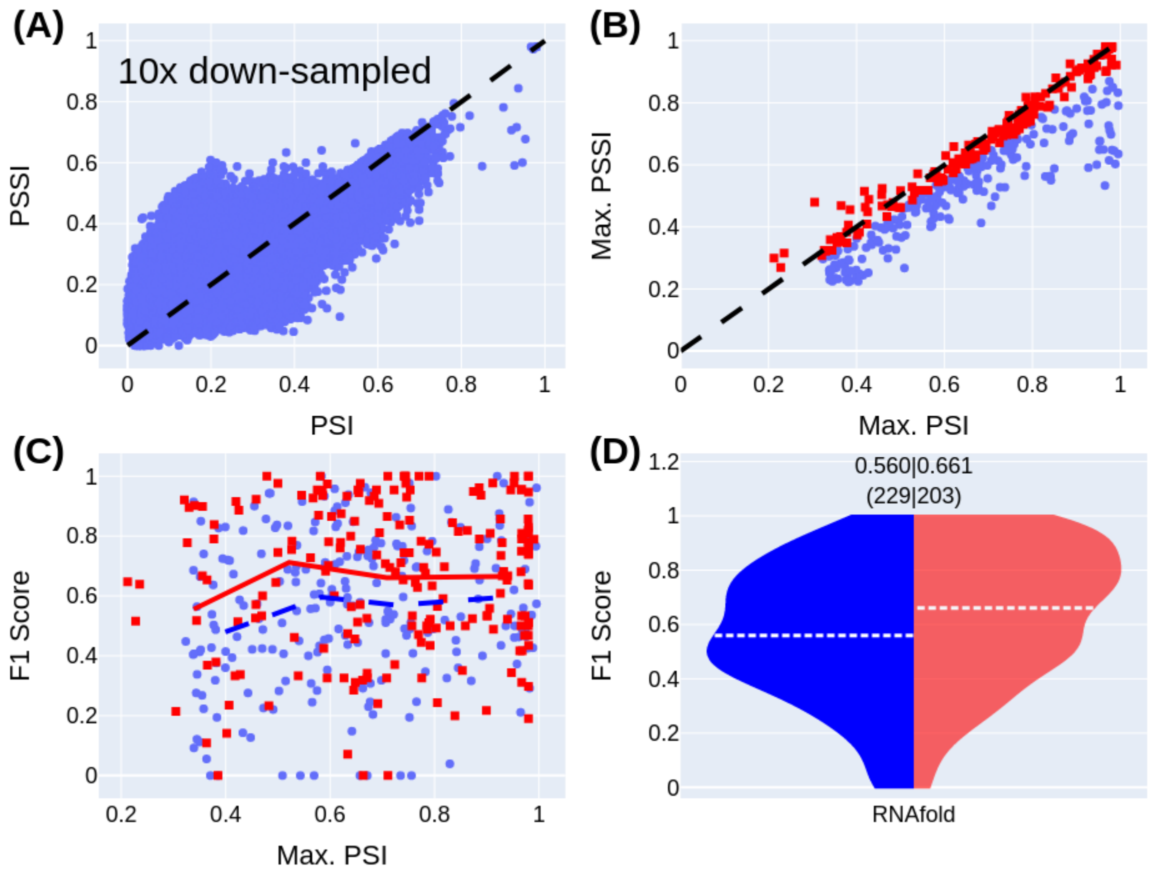

Fig JJ. Comparisons of the pairwise PSI (FoldAlign) and PSSI (RNAdistance) scores and the dependences of RNAfold performances on the similarities in RNA sequence and structure. Description of each panel follows that of Fig EE in S1 Text.

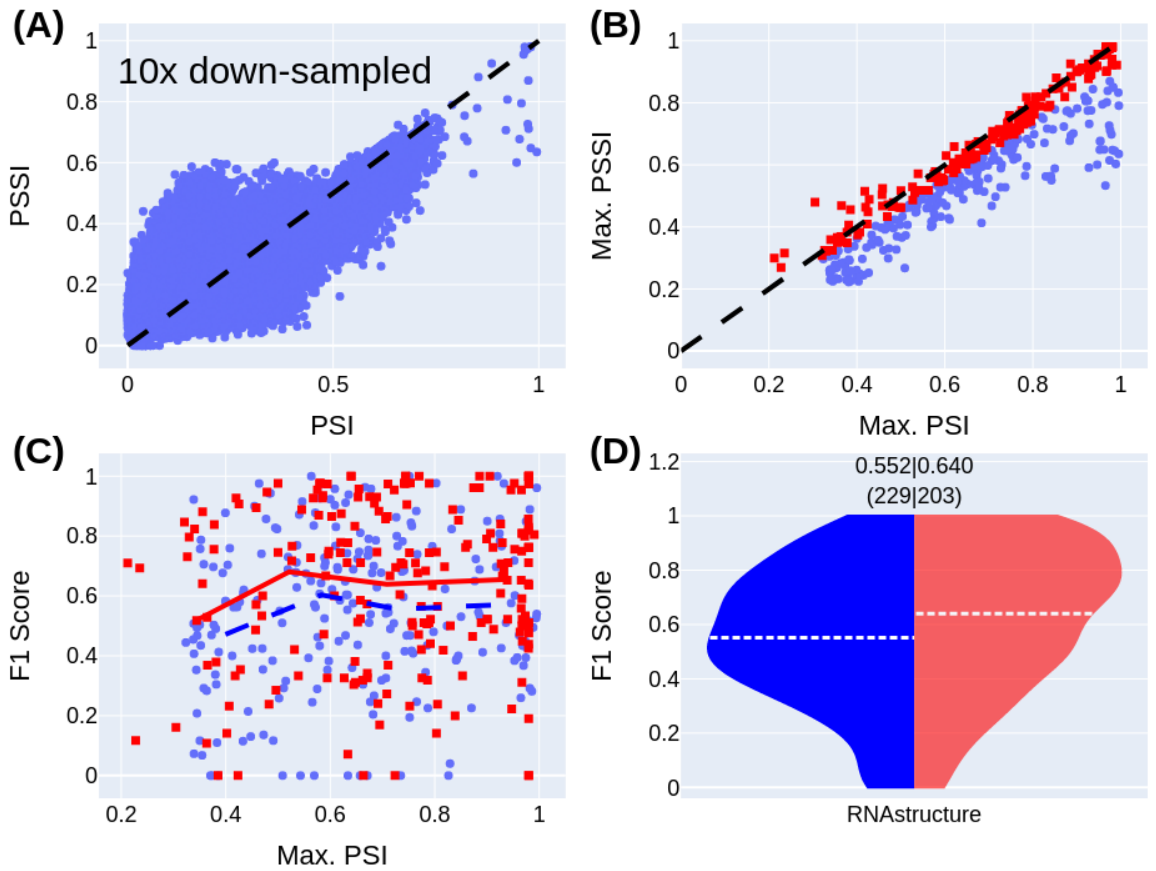

Fig KK. Comparisons of the pairwise PSI (FoldAlign) and PSSI (RNAdistance) scores and the dependences of RNAstructure performances on the similarities in RNA sequence and structure. Description of each panel follows that of Fig EE in S1 Text.

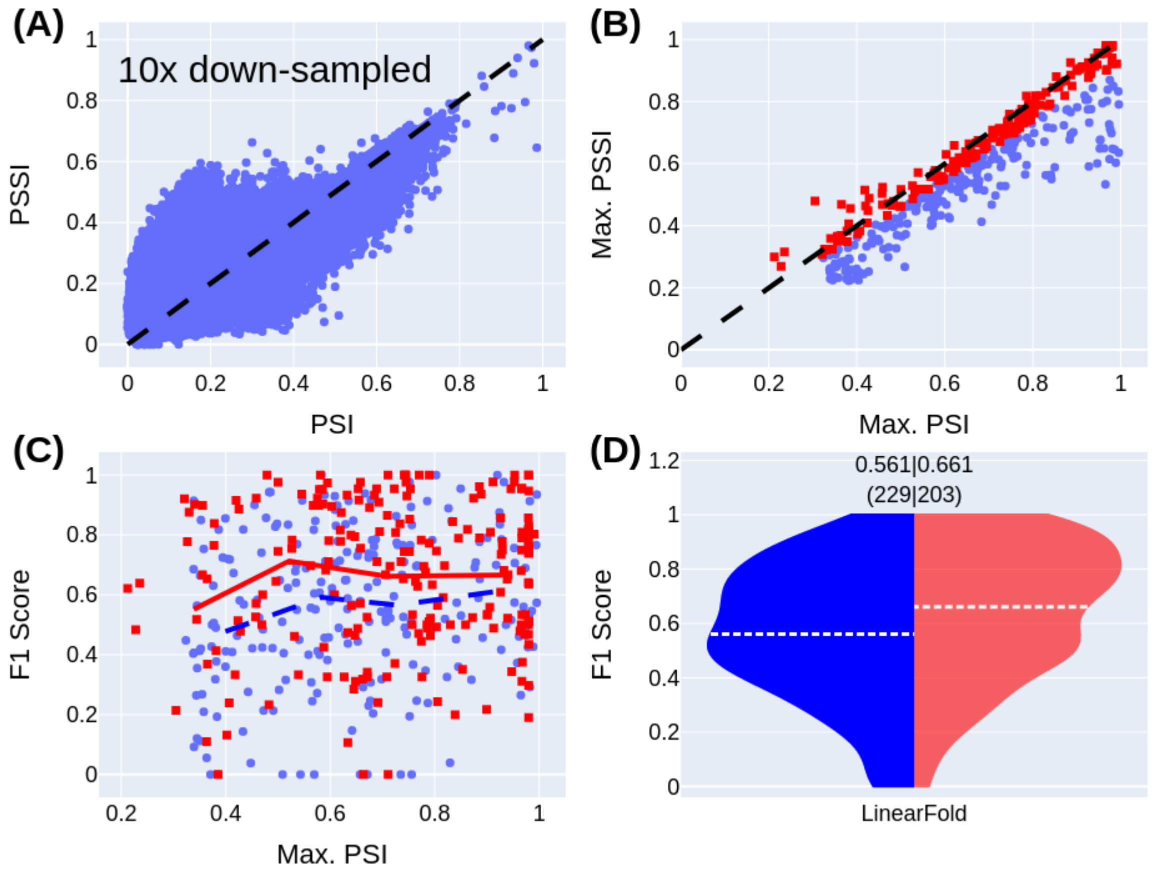

Fig LL. Comparisons of the pairwise PSI (FoldAlign) and PSSI (RNAdistance) scores and the dependences of LinearFold performances on the similarities in RNA sequence and structure. Description of each panel follows that of Fig EE in S1 Text.

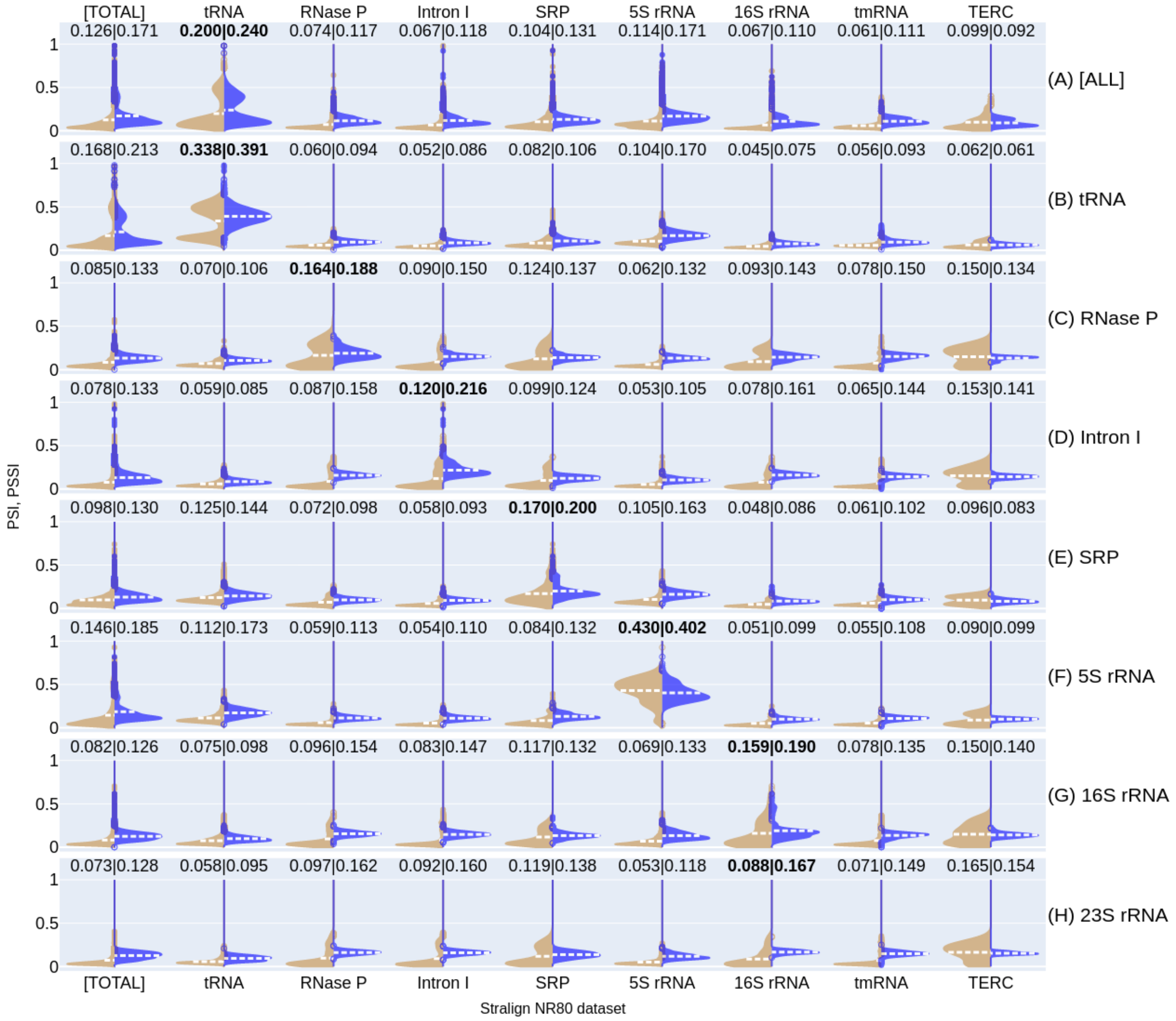

Fig MM. Comparisons of the PSI (by FoldAlign, left, tan) vs. PSSI (by RNAforester, right, blue) score distributions. Each distribution is generated from pairwise alignments between two datasets, the unseen and seen datasets. Each row/panel shows the results from one unseen set given by the label to the right (A-H). The unseen set for (A) [ALL] is the entire Archi-Stral-NR80 dataset (433 sequences) and the unseen sets for the other panels (B-H) are the labelled RNA families in the Archi-Stral-NR80 dataset. The seen dataset is the entire Stralign NR80 dataset ([TOTAL]) or the specific RNA family in Stralign NR80 given in the x axis label. The average PSI and PSSI values are shown above the violins and the largest PSSI value for each panel is shown in bold.

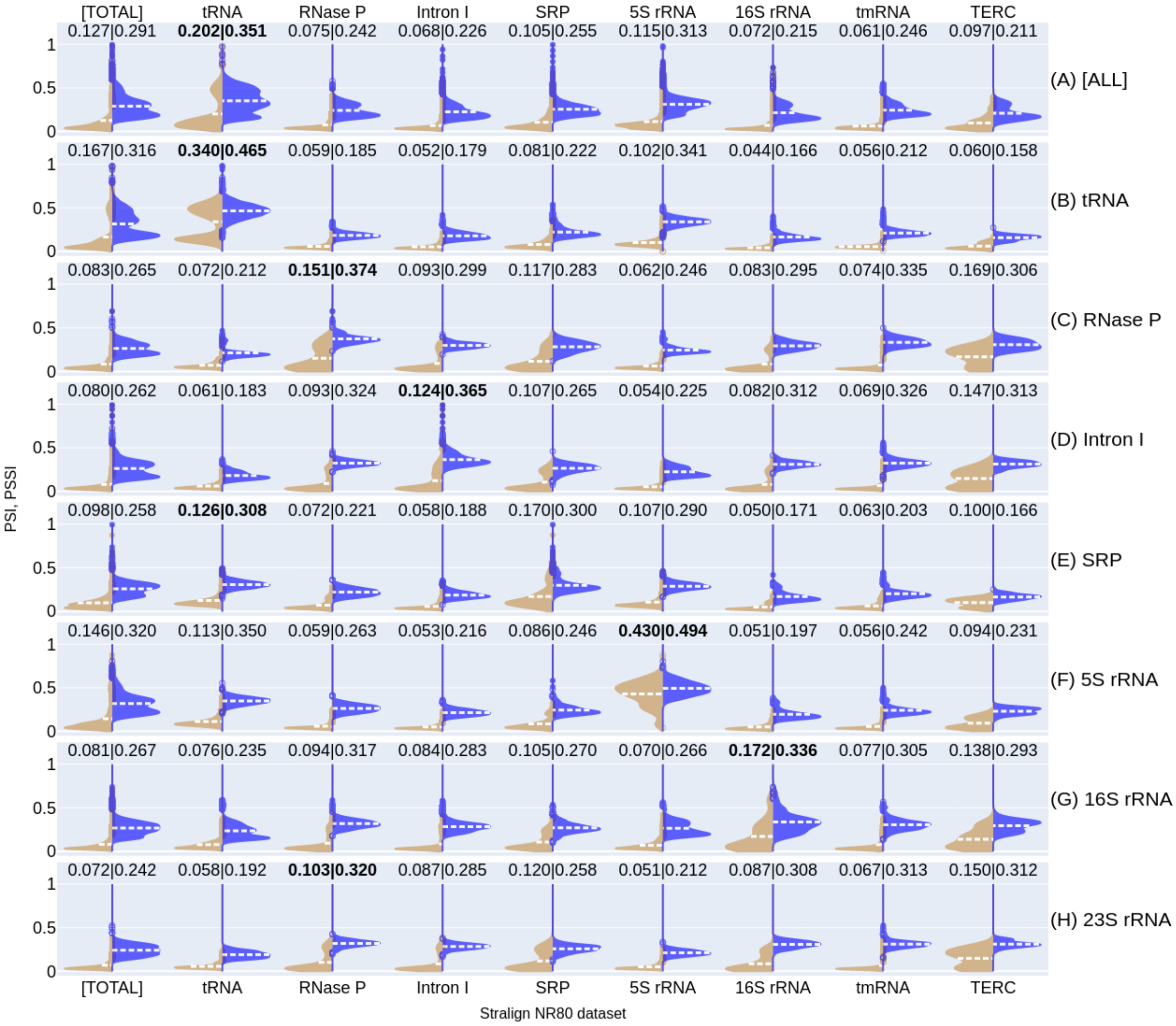

Fig NN. Comparisons of the PSI (by FoldAlign, left, tan) vs. PSSI (by RNAdistance, right, blue) score distributions. Captioning follows that of Fig MM in S1 Text.

Bibliography

1. Tan, Z., Fu, Y.H., Sharma, G. and Mathews, D.H. (2017) TurboFold II: RNA structural alignment and secondary structure prediction informed by multiple homologs. *Nucleic Acids Research*, **45**, 11570-11581.
2. Gardner, P.P., Wilm, A. and Washietl, S. (2005) A benchmark of multiple sequence alignment programs upon structural RNAs. *Nucleic Acids Res.*, **33**, 2433-2439.
3. Capriotti, E. and Marti-Renom, M.A. (2010) Quantifying the relationship between sequence and three-dimensional structure conservation in RNA. *BMC Bioinformatics*, **11**, 322.

4. Sloma, M.F. and Mathews, D.H. (2016) Exact calculation of loop formation probability identifies folding motifs in RNA secondary structures. *RNA*, **22**, 1808-1818.
5. Danaee, P., Rouches, M., Wiley, M., Deng, D., Huang, L. and Hendrix, D. (2018) bpRNA: large-scale automated annotation and analysis of RNA secondary structure. *Nucleic Acids Res.*, **46**, 5381-5394.
6. Singh, J., Hanson, J., Paliwal, K. and Zhou, Y. (2019) RNA secondary structure prediction using an ensemble of two-dimensional deep neural networks and transfer learning. *Nat Commun*, **10**, 5407.
7. Sato, K., Akiyama, M. and Sakakibara, Y. (2021) RNA secondary structure prediction using deep learning with thermodynamic integration. *Nat Commun*, **12**, 941.
8. Ouyang, Z., Snyder, M.P. and Chang, H.Y. (2013) SeqFold: genome-scale reconstruction of RNA secondary structure integrating high-throughput sequencing data. *Genome Res.*, **23**, 377-387.
9. Lin, T.-Y., Goyal, P., Girshick, R., He, K. and Dollár, P. (2017), *Proceedings of the IEEE international conference on computer vision*, pp. 2980-2988.
10. Fu, L., Cao, Y., Wu, J., Peng, Q., Nie, Q. and Xie, X. (2022) UFold: fast and accurate RNA secondary structure prediction with deep learning. *Nucleic Acids Res.*, **50**, e14.
